# A genomic survey of transposable elements in the choanoflagellate *Salpingoeca rosetta* reveals selection on codon usage

**DOI:** 10.1186/s13100-019-0189-9

**Published:** 2019-11-23

**Authors:** Jade Southworth, C. Alastair Grace, Alan O. Marron, Nazeefa Fatima, Martin Carr

**Affiliations:** 1 Department of Biological & Geographical Sciences, University of Huddersfield, Huddersfield, HD1 3DH UK; 2 Department of Plant Sciences, University of Cambridge, Cambridge, CB2 3EA UK; 3 Science for Life Laboratory, Department of Immunology, Genetics and Pathology, Uppsala University, Uppsala, Sweden

**Keywords:** Mutation pressure, Natural selection, Opisthokonta, Optimal codons, Pol, Tnpase

## Abstract

**Background:**

Unicellular species make up the majority of eukaryotic diversity, however most studies on transposable elements (TEs) have centred on multicellular host species. Such studies may have therefore provided a limited picture of how transposable elements evolve across eukaryotes. The choanoflagellates, as the sister group to Metazoa, are an important study group for investigating unicellular to multicellular transitions. A previous survey of the choanoflagellate *Monosiga brevicollis* revealed the presence of only three families of LTR retrotransposons, all of which appeared to be active. *Salpingoeca rosetta* is the second choanoflagellate to have its whole genome sequenced and provides further insight into the evolution and population biology of transposable elements in the closest relative of metazoans.

**Results:**

Screening the genome revealed the presence of a minimum of 20 TE families. Seven of the annotated families are DNA transposons and the remaining 13 families are LTR retrotransposons. Evidence for two putative non-LTR retrotransposons was also uncovered, but full-length sequences could not be determined. Superfamily phylogenetic trees indicate that vertical inheritance and, in the case of one family, horizontal transfer have been involved in the evolution of the choanoflagellates TEs. Phylogenetic analyses of individual families highlight recent element activity in the genome, however six families did not show evidence of current transposition. The majority of families possess young insertions and the expression levels of TE genes vary by four orders of magnitude across families. In contrast to previous studies on TEs, the families present in *S. rosetta* show the signature of selection on codon usage, with families favouring codons that are adapted to the host translational machinery. Selection is stronger in LTR retrotransposons than DNA transposons, with highly expressed families showing stronger codon usage bias. Mutation pressure towards guanosine and cytosine also appears to contribute to TE codon usage.

**Conclusions:**

*S. rosetta* increases the known diversity of choanoflagellate TEs and the complement further highlights the role of horizontal gene transfer from prey species in choanoflagellate genome evolution. Unlike previously studied TEs, the *S. rosetta* families show evidence for selection on their codon usage, which is shown to act via translational efficiency and translational accuracy.

## Background

Transposable elements (TEs) are repetitive mobile DNA sequences that are components of the majority of eukaryotic genomes. TEs can be categorised into two classes, distinguished by their method of transposition. Class I elements are retrotransposons, which transpose via an RNA intermediate; the retrotransposons can be further categorised by the presence or absence of long terminal repeats (LTRs), which flank the 5’and 3′ end of the element. Autonomous retrotransposons contain *gag* and *pol* open reading frames (ORFs) which respectively encode structural and enzymatic proteins that facilitate transposition. LTR retrotransposons may also exist as solo LTRs, due to recombination between the two LTR sequences of an individual element which results in the removal of an LTR sequence and the internal, coding DNA. Class II TEs are DNA transposons which transpose as a DNA copy, often by a “cut and paste” mechanism that results in the entire element being removed from the host chromosome and reinserted into a new position in the genome. This process is facilitated by a Transposase (Tnpase) protein for most DNA transposons, which binds to flanking inverted terminal repeats (ITRs) creating double-stranded breaks and allowing the integration of the transposon at a new genomic location.

TEs are major drivers of mutation within their host populations and, as such, individual copies may be subject to negative selection. Selection may operate against insertion mutations or ectopic recombination rearrangements [1, 2]. In addition, as a result of the metabolic burden of TE activity, selection may also operate against the transposition process itself [3].

To date TEs have predominantly been studied in multicellular organisms, with comparatively limited research into unicellular eukaryotes. A number of studies have investigated aspects of TE evolution within species across known eukaryotic diversity, including unicellular opisthokonts, amoebozoans, alveolates, stramenopiles and excavates [4–10]. Such studies have concentrated on the diversity of TEs present in the host genome and the use of phylogenetics to determine the evolutionary origin of the families. The population biology of the TEs in unicellular eukaryotes has received less attention. However phylogenetic evidence for recent transposition has been shown in the filasterean *Capsaspora owczarzaki* [4] and purifying selection on the amino acid sequences of TE proteins has been reported in the parabasalid *Trichomonas vaginalis* [9] as well as the the choanoflagellate *Monosiga brevicollis* [10]. In addition, gene expression studies have also shown that TE proteins are transcribed in the latter two species.

### TEs in holozoan protists

The eukaryotic supergroup Opisthokonta is composed of two major lineages in the Holozoa and Holomycota [11, 12]. The holozoans include Metazoa, as well as their unicellular relatives Choanoflagellatea, Filasteria, Ichthyosporea and Pluriformea [13]; Fungi and the nuclearioid amoebae make up the Holomycota [14]. Within Holozoa, choanoflagellates are the closest living known relatives to the metazoans, and provide insight into the origin of animals [15]. All choanoflagellates have unicellular stages in their lifecycle, however many species can develop ephemeral multicellular colonies [see 16 for a thorough review of the group].

*M. brevicollis* was the first unicellular holozoan to have its genome sequenced [17], allowing a study of its TEs. Only three families were identified, all of which were LTR retrotransposons [10]. The same study also screened available EST sequences from a second choanoflagellate, *Mylnosiga fluctuans* (erroneously described as *Monosiga ovata* [18]), uncovering LTR and non-LTR retrotransposons in addition to DNA transposons, suggesting that *M. brevicollis* may be atypical in having a limited diversity of TE families [10]. Further insight into the evolution of TEs in holozoan protists was provided by the genome analysis of *C. owczarzaki* [4]. The genome harboured 23 TE families, all of which were identified as having orthologous families in Metazoa and Fungi, indicating that the common ancestor of the opisthokonts had a diverse repertoire of mobile elements. Despite the difference in the number of families identified within the genomes of *M. brevicollis* and *C. owczarzaki*, copy numbers for individual families were similar across both species with all families being reported as possessing fewer than 100 copies. One notable difference between *M. brevicollis* and *C. owczarzaki* was the finding that all families in the former are active, whereas the latter contains at least one family that is no longer functional.

### Codon usage bias in *S. rosetta*

The degenerate nature of the genetic code results in all amino acids, with the exceptions of methionine and tryptophan, being encoded by more than one synonymous codon. Clark [19] predicted that a variety of forces may interact to dictate the non-random usage of codons and subsequent studies in a broad range of eukaryotes, prokaryotes and viruses have shown that codon usage tends to show a bias in most genes [20, 21]. The degree of bias in any given gene can be calculated using the “effective number of codons” (*N*_*c*_) statistic [22]. Values range from 20, where all amino acids only use a single synonymous codon, to 61 where each amino acid uses all synonymous codons equally. Any direction of bias may then be determined using GC3s, which is the measure of the proportion of guanosine and cytosine at synonymous third positions.

Codon usage bias may be determined by genetic drift, mutational pressure, selection for translationally optimal codons [23, 24], or a combination of the three forces. In species with a large effective population size (*N*_*e*_), selection tends to be the dominant force; however, in species with smaller effective population sizes, mutational pressure and drift may act to swamp any selective advantage for optimal codons [25]. Selection for translationally optimal codons may operate through efficiency, allowing the rapid synthesis of polypeptides [26], and accuracy, which reduces the likelihood of misincorporated amino acids [27]. The degree to which the codon usage of a gene shows adaptation to the host optimal codons can be calculated using the frequency of optimal codons (F_op_), which is the total number of optimal codons divided by the total number of codons in the sequence [23].

Patterns of relative synonymous codon usage for genes in *S. rosetta* and *M. brevicollis*, both of which are craspedid choanoflagellates [28], were established by Southworth et al. [29]. This study identified the suite of 23 translationally optimal codons for *S. rosetta* and 24 optimal codons for *M. brevicollis*, which are predominantly GC-ending. Selection was shown to operate at the level of both translational accuracy and efficiency.

### Codon usage bias in TEs

Past studies on TE codon usage bias have mainly failed to detect the action of natural selection; rather transposon and retrotransposon families, as well as LTR retroviruses, in a range of organisms show codon usage patterns similar to weakly expressed host genes and a slight excess of AT-ending codons [30–33]. One reported exception is the LTR retrotransposons of the stramenopile genus *Phytophthora* [34]. TE families within this group of unicellular eukaryotes were shown to have a preference for GC-ending codons that mirrors host genes; furthermore, high copy number families tended to show stronger codon usage bias than families with lower copy number. The study did not determine which, if any, of the *Phytophthora* codons were optimal codons or if the higher frequency of GC-ending codons was a result of the use of optimal codons. Due to the emphasis on multicellular eukaryotes within TE research, it is unknown if the putative selection observed on codon usage in *Phytophthora* families is commonplace or highly unusual within unicellular eukaryotes.

As the translationally optimal codons in *S. rosetta* mainly show guanosine or cytosine at synonymous sites, any selection for codon usage in the TE families should contrast sharply with the weak bias towards AT-ending codons previously observed in the majority of eukaryotic TEs. *S. rosetta* therefore provides an opportunity to determine if selection for codon usage is present in the TE families of unicellular eukaryotes other than those previously recorded in *Phytophthora*.

### Experimental overview

The presented study screened the genome of *S. rosetta* in order to identify the species’ TE complement. The TE families were phylogenetically analysed in an attempt to determine their evolutionary history. Evidence for recent element activity was gained through phylogenetic analyses of individual element insertions within the *S. rosetta* genome. The forces controlling codon usage within the TE genes were analysed to determine if selection for optimal codons was operating and if there was a contribution due to mutation pressure.

## Results

### *S. rosetta* harbours a greater diversity of TE families than *M. brevicollis*

The *S. rosetta* (ATCC 50818) genome was found to have a minimum of 20 full-length TE families when screened through both RepeatMasker and BLAST searches. Both methodologies identified the same families. The *S. rosetta* TEs were classified into 10 superfamilies (Tables 1 and 2; Additional files 1 and 2). The LTR retrotransposon families were named *Salpingoeca rosetta chromovirus*-*1* to *Salpingoeca rosetta chromovirus-5* (*Sroscv1*–*5*)*, Salpingoeca rosetta gypsy-like element-1* and *Salpingoeca rosetta gypsy-like element-2* (*Srosgyp1*–*2*) and *Salpingoeca rosetta pseudovirus-1* to *Salpingoeca rosetta pseudovirus-6* (*Srospv1*–*6*). The DNA transposon families were *Salpingoeca rosetta helitron* (*SrosH*), *Salpingoeca rosetta Harbinger Element* (*SrosHar*), *Salpingoeca rosetta MULE-like element* (*SrosM*), *Salpingoeca rosetta Sola1 Element* (*SrosS*), *Salpingoeca rosetta Tigger Element-1*, *Salpingoeca rosetta Tigger Element-2* (*SrosTig1*–*2*) and *Salpingoeca rosetta Tc1*/*mariner Element* (*SrosTm*). Partial *pol* sequences from two putative families of SLACS non-LTR retrotransposon were identified (Supercont1.5, NW_004754929.1, nucleotides 598,557–600,491; Supercont1.6, NW_004754928.1, nucleotides 201,841–202,980), however complete full-length consensus sequences could not be reconstructed from Trace Archive sequencing reads due to poor coverage. As the two families remain to be fully sequenced, they have not been considered in the remainder of the presented work. Both MITE-Hunter [35] and MITE Tracker [36] failed to identify any MITE families in the *S. rosetta* genome.

**Table 1:** Characterisation of the LTR retrotransposon families identified within the *S. rosetta* genome

Family	Length (bp)	LTR Size	Copy Number (FLE/Solo LTR/ Truncated)	No. of Identical Paralogous Copies	Number of Sequence Reads	Intra-element LTR Identity	LTR Nucleotide Diversity ( π - Total/FLE/Solo)		
*Sroscv1*	5290	243	16 (8/5/3)	10	457	100	0.022	0.006	0.048
*Sroscv2*	4813	190	7 (4/0/3)	2	243^a^	100	0.122	0.122	–
*Sroscv3*	5165	394	21 (10/10/1)	12	1288	99.2–100	0.019	0.006	0.030
*Sroscv4*	5965	580	15 (12/1/2)	2	92	99.6	0.039	0.039	–
*Sroscv5*	6071	408	9 (6/2/1)	2	1591^a^	99.5–100	0.026	0.025	–
*Srosgyp1*	5460	373	9 (6/2/1)	4	197^a^	100	0.017	0.003	–
*Srosgyp2*	5169	385	6 (6/0/0)	4	856	100	0.000	0.000	–
*Srospv1*	4943	387	14 (11/0/3)	2	327	99.7–100	0.016	0.016	–
*Srospv2*	5452	554	71 (41/4/26)	17	4211	99.6–100	0.044	0.041	0.077
*Srospv3*	5680	445	121 (59/14/48)	18	5182	94.2–100	0.083	0.084	0.079
*Srospv4*	5148	359	4 (1/3/0)	2	48	100	0.001	–	0.002
*Srospv5*	5088	362	3 (2/0/1)	2	1082	100	0.000	–	–
*Srospv6*	11,222	168	1 (1/0/0)	0	n/a^b^	100	–	–	–

^a^ Read number based upon edited ORF sequences. ^b^ n/a: Expression level is not shown for *Srospv6*, as it is a pseudogene

**Table 2:** Characterisation of the DNA transposon families identified in the *S. rosetta* genome

Family	Length	ITR Size	TSD Length^a^	Copy Number (5′ ITR/3′ ITR)	No. of Identical Paralogous Copies^b^	Number of Sequence Reads^c^	ITR Nucleotide Diversity (π)^d^
*SrosH*	3761	n/a	n/a	6–9 (6/3)	n/a	1402	–
*SrosHar*	3112	27	3	1–2 (1/1)	n/a	1	–
*SrosM*	8326	28	9	23 (13/16)	9	19,480	0.054
*SrosS*	3270	32	4	2 (2/2)	0	7946	–
*SrosTig1*	2122	22	2	7–11 (7/4)	0	6941	0.005
*SrosTig2*	2164	23	2	4–7 (4/3)	0	78	0.050
*SrosTm*	2071	28	n/a	8–14 (7/8)	4	15,354	0.008

^a^ Target site duplications could not be identified for *SrosH* and *SrosTm*. ^b^ The number of identical paralogous copies could not be determined for *SrosH* and *SrosHar*. ^c^ Read number based upon edited gene regions for *SrosS*. ^d^ Nucleotide diversity could not be determined for *SrosH* due to low sequencing reads at the element termini; diversity could not be determined for *SrosS* and *SrosHar* due to their low copy numbers

The predicted full-length LTR retrotransposons elements range from 4.8 to 11.2 kb in length (Table 1, Additional files 1 and 2). *Sroscv4* and *Sroscv5* both encode *gag* and *pol* in separate frames, with 6 bp slippage motifs facilitating the ribosomal frameshift (Additional file 2); the other LTR retrotransposon families all encode *gag* and *pol* within the same reading frame. *Srospv6* possesses a *gag*-*pol* pseudogene and appears to be the only clearly non-functional family within the sequenced strain (Additional files 1 and 2). The *gag*-*pol* pseudogene contains five regions of trinucleotide repeats (Additional file 1) which contribute in part to the much greater length of this family (11.2 kb) compared the other identified TE families in the sequenced genome. Upon integration, all *copia*-like and chromoviral families create a 5 bp target site duplication (TSD), whereas the two non-chromoviral *gypsy*-like families generate 4 bp TSDs. Of the 13 LTR retrotransposon families, eight were present as both full-length elements (FLEs) and solo LTRs; *Sroscv2*, *Srosgyp2*, *Srospv1*, *Srospv5* and *Srospv6* all appeared to lack solo LTRs within the sequenced strain of *S. rosetta*. Ten of the families also showed truncated insertions, with deletions disrupting their sequences (Table 1).

The DNA transposon families ranged from 2.1 to 8.3 kb in length (Additional files 1 and 2). The transposon families each possessed a single gene, which encoded a putative Tnpase. Four of the seven genes contained introns, with *SrosTm* and the two *Tigger*-like families lacking introns. The helitron transposon family, *SrosH*, did not possess terminal repeats, whilst all other DNA transposon families had flanking ITRs. TSD length varied between 2 and 9 bp in length; however two families, *SrosH* and *SrosTm*, which were both multicopy, did not generate identifiable TSDs upon integration (Table 2).

Although *S. rosetta* exhibits a far greater diversity of TE families than *M. brevicollis*, the copy numbers of individual families were broadly similar within the two choanoflagellates. With the exception of two high-copy number families of *copia*-like LTR retrotransposon (*Srospv2* and *Srospv3*), all families in both species have less than 50 copies within the sequenced genomes (Tables 1 and 2, [10]). Similar copy numbers were also reported for the filasterean *C. owczarzaki* [4], raising the possibility that low TE copy numbers are prevalent in holozoan protist genomes.

### *S. rosetta* TE families have diverse evolutionary origins

**Fig. 1:**
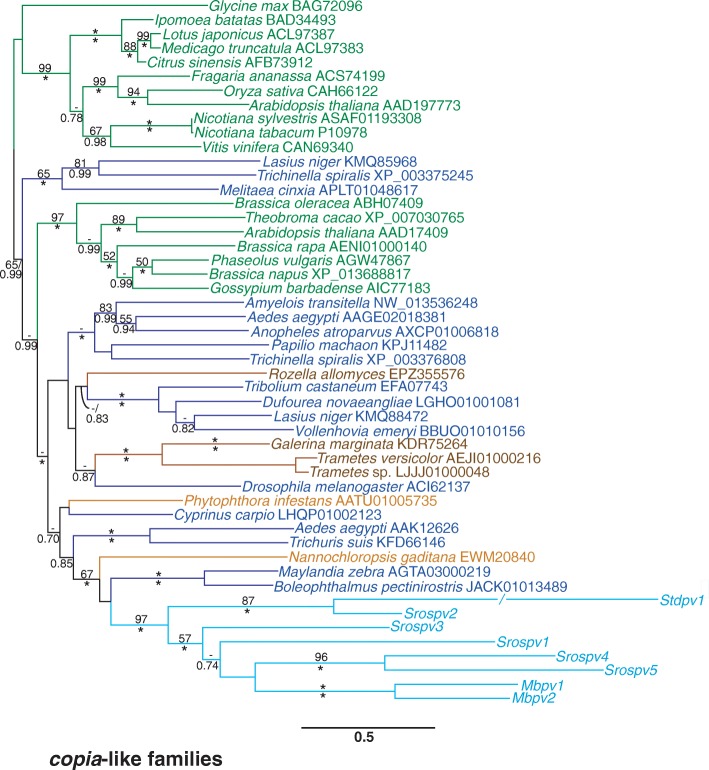
Maximum likelihood phylogeny of the *copia*-like superfamily. The phylogeny was constructed from 493 aligned amino acid positions using the PROTCAT model, and estimated amino acid frequencies, with the RTREV substitution matrix. Values for mlBP and biPP are shown above and below the branches respectively. 100% mlBP and 1.00 biPP are both denoted by “*”. Values < 50% mlBP and < 0.70 biPP are denoted by “-”. Choanoflagellate proteins are written in *light blue* font. Metazoan proteins are written in *dark blue*, fungal proteins in *brown*, stramenopile proteins in orange and archaeplastid proteins in green

**Fig. 2:**
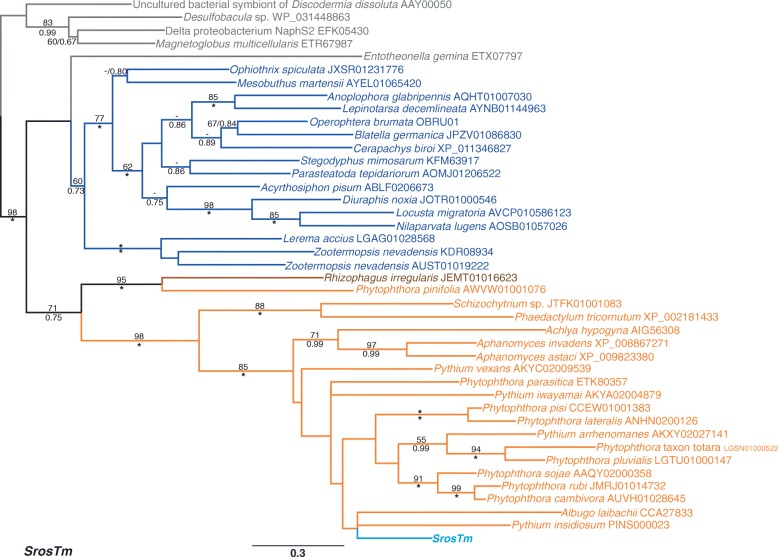
Maximum likelihood phylogeny of the *Tc1*/*mariner* superfamily containing *SrosTm*. The phylogeny was constructed from 258 aligned amino acid positions using the PROTCAT model and estimated amino acid frequencies with the WAG substitution matrix. Bacterial proteins are written in grey, the layout is otherwise in the same format as Fig. 1

Protein phylogenies were created for all TE superfamilies present in the *S. rosetta* genome, using Pol for LTR retrotransposons and Tnpase for DNA transposons (Figs. 1 and 2; Additional file 3). The *copia*-like Pol sequences from the craspedid choanoflagellates *M. brevicollis* and *S. rosetta*, as well as *Stephanoeca diplocostata*, a choanoflagellate from the Acanthoecida order [28], cluster together with strong support (97% maximum likelihood bootstrap percentage (mlBP) and 1.00 Bayesian Inference posterior probability (biPP), Fig. 1). The two *M. brevicollis* families formed a strongly supported group (100% mlBP/1.00 biPP). However, the monophyly of the *S. rosetta* families was rejected (87% mlBP/1.00 biPP), as *Srospv2* was recovered as a closer relative of *Stdpv1* from *St. diplocostata* than the *copia*-like familes from the craspedids *S. rosetta* and *M. brevicollis*. The closest relatives of the choanoflagellate *copia*-like families are predominantly from other opisthokont taxa, consistent with vertical inheritance since the opisthokont last common ancestor (LCA); however, this grouping is recovered with weak to strong support (< 50% mlBP/1.00 biPP) and also contains two Pol sequences from the stramenopiles *Nannochloropsis gaditana* and *Phytophthora infestans*.

In contrast to the *copia*-like phylogeny, the tree for the *Tc1*/*mariner* transposon *SrosTm* did not cluster the *S. rosetta* protein with those from other opisthokonts (Fig. 2). BLAST screening uncovered putative Tnpase sequences from metazoans, fungi, stramenopiles and bacteria, with the *SrosTm* Tnpase protein nested with strong support within the stramenopile proteins (85% ML/1.00 biPP). Furthermore, the *SrosTm* Tnpase is separated from the metazoan Tnpase sequences by two strongly supported branches (98% mlBP/1.00 biPP and 85% mlBP /1.00 biPP).

A predicted protein (XP_001743358) was also uncovered from *M. brevicollis* through BLAST, however an inspection of the annotated sequence indicates the predicted gene may in fact be a pseudogene. The gene annotation predicts an in-frame intron between nucleotides 984–1049, between the predicted second and third exons; however the translation of the putative intron sequence possesses 45.5% identity and 68.2% similarity to the homologous *SrosTm* amino acids if it is translated as part of the second exon (Additional file 4). Furthermore, the predicted intron sequence contains an in-frame premature stop codon. If the intron is genuine, then ten conserved residues would be absent from the truncated Tnpase. However, if the intron is a mis-annotation, then the Tnpase would be shortened by 315 amino acid residues due to the in-frame stop codon. Including the putative *M. brevicollis* protein in the *Tc1*/*mariner* superfamily phylogeny has limited effect on the support for the choanoflagellate Tnpase being nested within the stramenopile proteins (84% mlBP/1.00 biPP, Additional file 3). The presence of *tnpase* sequences from two craspedid choanoflagellates nested with strong support within stramenopile sequences is consistent with the horizontal transfer of a transposon from the latter group into the former.

Neither the chromoviral nor the non-chromoviral *gypsy*-like family phylogenies were robustly resolved, however in both trees the choanoflagellate Pol sequences were recovered as monophyletic (Additional file 3). In the chromoviral phylogeny the *S. rosetta* families clustered with *Mbcv*, previously identified in *M. brevicollis* [10], however the grouping lacks phylogenetic support (< 50% mlBP/< 0.70 biPP). As with the *copia*-like families, the closest relatives of the *S. rosetta gypsy*-like families are predominantly present in the genomes of other opisthokont species, consistent with their vertical inheritance since the opisthokonts last shared a common ancestor.

With the exception of *SrosTm*, the transposon family phylogenies could not robustly place the *S. rosetta* Tnpase sequences (Additional file 3). The Tnpase proteins of *SrosH*, *SrosM*, *SrosS*, *SrosHar* and *SrosTig2* clustered with weak to moderate support (< 70%mlBP, < 0.70biPP) with families mainly uncovered from other opisthokont genomes. *SrosTig1* also clustered with Tnpases recovered from opisthokont genomes, however sequences from stramenopiles were the closest proteins in the phylogeny (< 50%mlBP, 0.79biPP). The inheritance of *SrosTig1* is therefore unresolved.

### TE activity and expression in the *S. rosetta* genome

All three TE families identified in the *M. brevicollis* genome appeared to be active [10], however TEs are considered to have their own life cycles within their host genome [37] resulting in some families eventually being extinct relics. Each family in the *S. rosetta* genome was therefore examined for evidence of recent activity.

LTR sequences are identical upon the integration of a daughter element, and subsequently accumulate mutations over time [38]. Intra-element LTR identity ranged from 94.2 to 100% across the retrotransposon families (Table 1), highlighting their recent transposition. *Srospv3* was the only family where insertions showed lower than 99% intra-element LTR identity. The majority of mismatches between LTRs from the same insertions within *Srospv3* are due to indels in and around a highly repetitive region of CA repeats (bases 303–406 in the annotated sequence in Additional file 2). When the indels are excluded, intra-element LTR nucleotide identity is over 99% for 6 out of the 7 elements where comparisons could be made (data not shown). The lower nucleotide identity between LTRs from the same insertions of *Srospv3* may therefore not reflect their greater age, but perhaps is a result of slippage mutations occurring during reverse transcription. *Srospv6* lacks a functional *gag-pol* ORF, however it appears that the single copy in the genome has integrated recently as it possesses both 100% identical LTRs and target site duplications.

Intra-element LTR identity as a method for detecting recent transposition is restricted to LTR retrotransposons, however phylogenies of individual copies within a family can also provide insights into the recent evolutionary history of a family [4, 39]. In particular, identical sequences at different locations within the genome highlight on-going transposition, whilst sequences placed on short terminal branches indicate recent transposition events. Phylogenies were generated for 16 of the 20 families where full-length sequences have been reconstructed (Additional file 5). Due to either low copy number or poor sequencing quality phylogenies could not be created for *SrosH*, *SrosHar*, *Srospv6* and *SrosS* .

All of the *gypsy*-like families showed multiple identical sequences at different genomic locations, as do four of the *copia*-like families (Table 1, Additional file 5). *Srospv6*, as mentioned above, is a single copy, non-functional family. *Srospv1* does not possess identical paralogous copies; the greatest LTR identity between paralogous copies is 98.97% (Additional files 5 and 6), indicating no copies in the genome show very recent common ancestry.

In contrast to the retrotransposons, only two DNA transposon families, *SrosM* and *SrosTm*, were found to possess identical paralogous copies (Table 2). Phylogenies could not be created however for *SrosH*, *SrosS* and *SrosHar*. The quality of sequencing reads for the flanking DNA was inadequate for the creation of reliable phylogenies in the case of *SrosH* whilst *SrosHar* appears to be a single copy family. *SrosS* only has two copies in the genome and the terminal 300 bp 5′ ITR/UTRs share 98.3% nucleotide identity (Additional file 6), suggesting that the family has not transposed recently within the sequenced strain of *S. rosetta*.

**Fig. 3:**
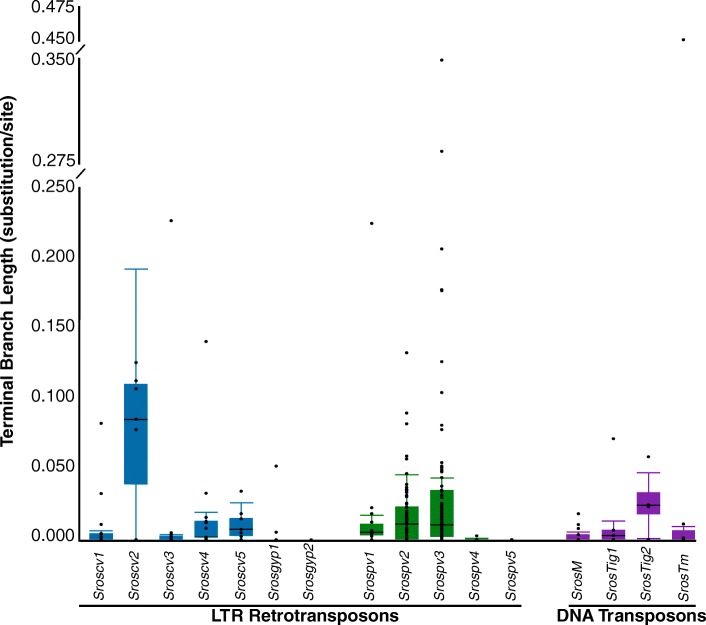
Terminal branch lengths of the 16 multicopy transposable element families in the *S. rosetta* genome. *Gypsy*-like retrotransposon, *copia*-like retrotransposon, and DNA transposon families are represented by *blue*, *green* and *purple boxes* respectively. Branch lengths for full-length LTR retrotransposons were taken from the 5′ LTR when this was present in the phylogeny; in its absence, the 3′ LTR was used. The filled *boxes* denote the interquartile range and the *horizontal dark line* represents the median branch length. The whiskers highlight 1.5 times the interquartile range from the median

All generated nucleotide phylogenies were composed of short branched, presumably young elements, with few ancient copies (as defined in Carr and Suga [4] as having a terminal branch length ≥ 0.05 substitutions per site) present in the genome (Fig. 3, Additional file 5). Within the LTR retrotransposon families *Srospv1–3*, *Sroscv1–4* and *Srosgyp1* all contained ancient copies that appear to have existed in the *S. rosetta* genome for long periods of time. With the exception of two copies of *Sroscv2* all of the long branched insertions were either solo LTRs or truncated elements (Additional file 5). Of the four DNA transposon family phylogenies, *SrosTm*, *SrosTig1* and *SrosTig2* all exhibited ancient elements, with only *SrosM* presenting entirely young copies.

Nucleotide diversity values, based upon Nei’s π [40] were generated for all LTR retrotransposon families other than the single copy *Srospv6*. Values were produced for all insertions, using only a single LTR from FLEs, as well as the FLEs and the solo LTRs (Table 1). Diversity was lower for the LTRs sequences from full-length elements when compared to solo LTRs in *Sroscv1*, *Sroscv3* and *Srospv2*, however in *Srospv3* the FLE LTRs harboured greater diversity than the solo LTRs. The *Srospv3* phylogeny shows deep population structure, with full-length elements falling into two distinct subgroups (labelled *Srospv3A* and *Srospv3B*), explaining the high pairwise nucleotide identity within the family (Additional file 5). The two subfamilies harbour identical copies at different genomic locations, indicating that both are currently transposing within the *S. rosetta* population. Nucleotide identity between the two subfamilies varies sharply across the length of their LTRs. Representative LTRs from full-length elements share 98.8% nucleotide identity over the terminal 166 bp at the 3′ end of the *Srospv3A* LTR, however the same LTRs only share 40.7% identity at the upstream 167 bp of the 5′ end (Additional file 6). The observed abrupt change in nucleotide identity across an LTR sequence is similar to the situation observed between *Ty1* and the hybrid *Ty1*/*2* elements in *Saccharomyces cerevisiae* [41] and is consistent with one of the subfamilies being composed of chimeric, recombinant elements. Within the DNA transposons, *SrosTm* and *SrosTig1* have low nucleotide diversity (Table 2), consistent with their populations being composed of mainly of short-branched, presumably young, insertions (Additional file 5). *SrosM* and *SrosTig2* both exhibit higher levels of nucleotide diversity, due to the presence of a greater proportion of long-branched copies that have accumulated unique mutations.

*S. rosetta* RNA-Seq reads (SRX042046–54, SRX3432761–2), with a combined total dataset of 74,845,386 reads, were screened for the 19 TE families with full-length consensus *pol* or *tnpase* sequences. *Srospv6* was also shown to be expressed (data not shown), however the single copy is a pseudogene so it was not analysed further. Sequencing reads could be mapped onto all families in the *S. rosetta* genome (Tables 1 and 2). Absolute numbers of mapped reads for all *pol* and *tnpase* could not be determined, due to the presence of highly repetitive sequences in the families *Srosgyp1*, *Sroscv2*, *Sroscv5* and *SrosS*. The repetitive regions mapped to a much greater number of sequencing reads in comparison to the remaining regions of the same families, presumably due to their additional presence in non-TE transcripts. The presented number of sequencing reads in Tables 1 and 2 are those generated once the repetitive regions had been omitted from the CDS regions. As the edited sequences are incomplete, they can therefore only be considered to provide an approximation of the genuine expression levels. Within the LTR retrotransposons, gene expression varied by two orders of magnitude, with *Srospv4* exhibiting less than 50 reads across the 11 SRA transcriptome runs (Table 1) and *Srospv3* mapping to over 5000 reads (Table 1). An even greater range of expression was observed within the DNA transposon families, with only single read mapping onto *SrosHar* whilst 19,480 reads were mapped to *SrosM* (Table 2).

### *S. rosetta* transposable element families show a similar pattern of codon usage bias to host genes

**Fig. 4:**
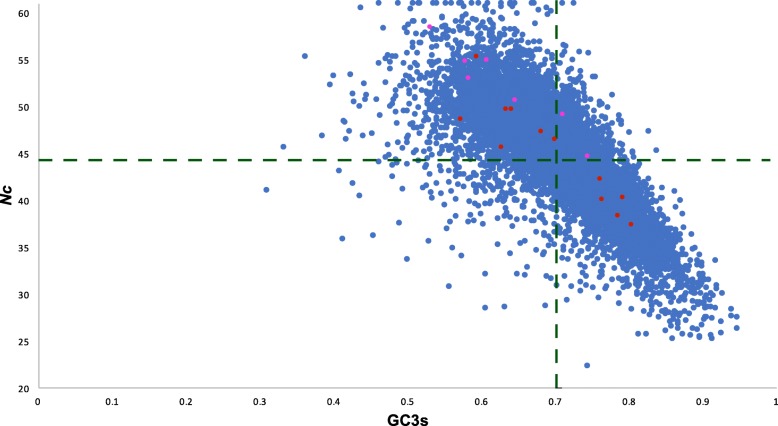
*S. rosetta Nc* plot highlighting the TE families present in the genome. *Blue dots* represent host genes, red dots show values for LTR retrotransposons and pink dots DNA transposons. *Dotted lines* show the genome average values (taken from Southworth et al. [29])

Host gene codon usage bias in *S. rosetta* is driven by natural selection at the levels of translational accuracy and efficiency [29]. Host genes show a strong relationship between codon usage bias and GC-content at synonymous sites and a remarkably similar association between GC3s and *N*_*c*_ was observed for the *S. rosetta* transposable element genes (Fig. 4). The TE genes show a stronger relationship between GC3s and *N*_*c*_ than the *S. rosetta* host genes (*R*^2^ = 0.804 and *R*^2^ = 0.610 respectively, Additional file 7). Earlier findings on TE codon usage in a broad range of eukaryotes have reported a weak AT-preference, however the *S. rosetta* families all exhibit an excess of GC-ending codons (Table 3). A higher GC3s content is observed in the LTR retrotransposons than in the transposons (Fig. 4; Table 3). In contrast to the GC bias at synonymous 3rd positions, mean non-coding GC content (LTR along with UTR sequences for LTR retrotransposons; ITR, UTR and introns for DNA transposons) is close to 0.5 for both classes of TE (Table 3). The higher GC3s in LTR retrotransposons is reflected in their level of codon usage bias. The mean *N*_*c*_ for LTR retrotransposon families (45.40 ± 5.58) is similar to the genome-wide mean *N*_*c*_ of 44.79 ± 5.37 [29]; however, the mean *N*_*c*_ for transposon families (52.37 ± 4.47) is considerably higher (Table 3).

**Table 3:** Codon usage statistics for the TE families identified in the *S. rosetta* genome

	*N*_*c*_	F_op_	GC3s	Non-Coding GC Content
LTR Retrotransposon Families				
*Sroscv1*	49.12	0.569	0.634	0.486
*Sroscv2*	45.22	0.598	0.627	0.450
*Sroscv3*	55.48	0.526	0.594	0.494
*Sroscv4*	42.06	0.694	0.763	0.552
*Sroscv5*	46.35	0.620	0.701	0.485
*Srosgyp1*	40.27	0.698	0.791	0.492
*Srosgyp2*	40.10	0.672	0.768	0.541
*Srospv1*	46.84	0.603	0.682	0.491
*Srospv2*	38.05	0.729	0.784	0.497
*Srospv3*	37.03	0.746	0.805	0.489
*Srospv4*	48.09	0.450	0.570	0.483
*Srospv5*	49.28	0.550	0.641	0.466
Mean	44.82 ± 5.45	0.621 ± 0.089	0.697 ± 0.083	0.494 ± 0.028
Transposon Families				
*SrosH*	45.17	0.615	0.740	0.555
*SrosHar*	58.55	0.448	0.530	0.514
*SrosM*	55.00	0.522	0.620	0.540
*SrosS*	50.80	0.565	0.654	0.529
*SrosTig1*	54.90	0.505	0.578	0.483
*SrosTig2*	52.47	0.488	0.578	0.517
*SrosTm*	49.61	0.612	0.709	0.486
Mean	52.14 ± 4.47	0.581 ± 0.077	0.63 ± 0.08	0.518 ± 0.027

### Mutation pressure influences codon usage bias in *S. rosetta* LTR retrotransposon families

The codon usage of TE families in *S. rosetta* is likely to be driven by one, or a combination, of genetic drift, mutation pressure, or selection for translationally optimal codons. Genetic drift cannot be ruled out, but appears unlikely as it is a random process and the 19 examined TE genes show an excess of GC-ending codons.

**Fig. 5:**
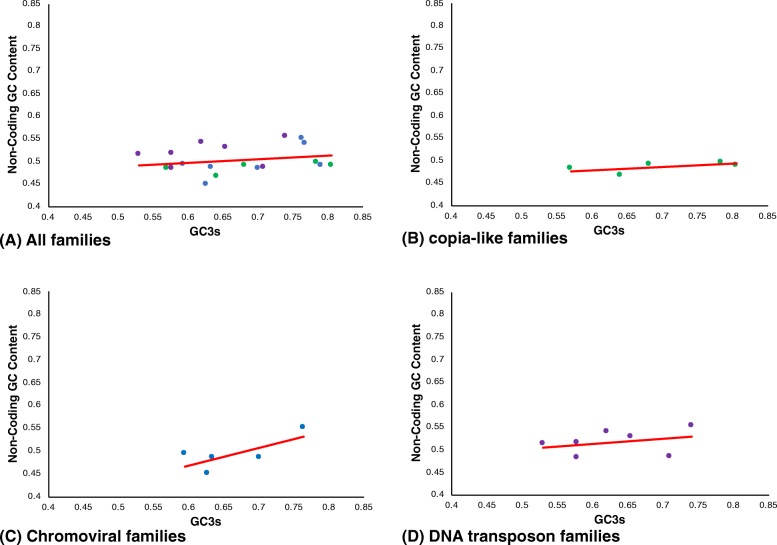
Values of non-coding GC content plotted against GC3s for TE families in the *S. rosetta* genome. **a** All TE families. **b** *copia-*like families. **c** Chromoviral families. **d** DNA transposon families. *Green dots* represent *copia*-like families, *blue dots* represent *gypsy*-like families and *purple dots* represent DNA transposons. The linear line of best fit is shown in *red*

Mutation pressure from AT to GC would be expected to affect non-coding DNA, in addition to synonymous 3rd codon positions. There is no clear relationship between the non-coding GC-content and GC3s across all families (Fig. 5, *R*^2^ = 0.050). The *copia*-like families showed a very weak positive relationship between GC3s and non-coding GC-content (*R*^2^ = 0.337), however the range in GC3s values across families is more than sevenfold greater than the range in non-coding GC-content. The chromovirus families show a stronger positive relationship between non-coding GC-content and GC3s (*R*^2^ = 0.530), therefore mutation pressure towards guanosine and cytosine may make a greater contribution towards codon usage bias in chromoviruses than *copia*-like families. The positive relationship for the chromoviruses is heavily dependent upon a single outlier, *Sroscv4*, and no relationship is recovered when the other families are considered alone and *Sroscv4* omitted (*R*^2^ = 1e^− 5^). The two non-chromoviral *gypsy*-like families show a negative relationship between non-coding GC-content and GC3s (data not shown); furthermore, the transposon families also failed to recover any relationship (*R*^2^ = 0.077); indicating that mutational pressure is not a major driver of codon usage bias towards their GC-ending codons.

### Natural selection influences codon usage bias in *S. rosetta* TE families

The most abundant codon for each amino acid was determined for *pol* in LTR retrotransposons and *tnpase* in DNA transposons (Additional file 8). The identified codon was then compared to the *S. rosetta* major tRNA genes and optimal codons, identified in Southworth et al. [29]. In all LTR retrotransposon families the majority of amino acids preferred codons complementary to the major tRNA genes for the *pol* ORFs. When host optimal codons were also considered, 17 out of 18 amino acids in the *pol* ORFs of *Srospv2* and *Srospv3*, as well as the two non-chromoviral *gypsy*-like families, were found to prefer codons that should result in efficient translation.

*SrosHar* was the only DNA transposon that did not have a majority of amino acids where the most abundant codon complemented the major tRNA genes. As noted above, *SrosHar* appears to be transcriptionally silent, as only a single read, spanning 20 bp, was mapped to the *tnpase* gene in 11 transcriptome runs. However when the *S. rosetta* optimal codons were also considered, the majority of amino acids preferred translationally efficient codons in all *tnpase* genes. The TE families’ preferences for codons employed by highly expressed host genes are reflected by their F_op_ values (Table 3). Across the 19 families, F_op_ ranges from 0.448 to 0.746, with LTR retrotransposons showing higher mean F_op_ values (0.621 ± 0.089) than the DNA transposons (0.581 ± 0.077).

The strength of codon usage bias (using F_op_) and family copy number show a very weak positive relationship for the LTR retrotransposons (*R*^2^ = 0.391, Additional file 9), however no such relationship was recovered for the DNA transposon families (*R*^2^ = 0.210). Investigating families within the two classes of TE individually shows greater heterogeneity within the relationships between F_op_ and copy number. The *copia*-like families show a strong positive relationship (*R*^2^ = 0.879) between copy number and use of optimal codons, whereas no relationship is recovered when the chromovirus families are considered in isolation (*R*^2^ = 0.295). Within the two non-chromoviral *gypsy*-like families, the family with the higher copy number (*Srosgyp1*) exhibits the higher value of F_op_. The higher use of translationally optimal codons therefore is consistent with them conferring an advantage in transposition for some families over others within the *S. rosetta* genome.

**Fig. 6:**
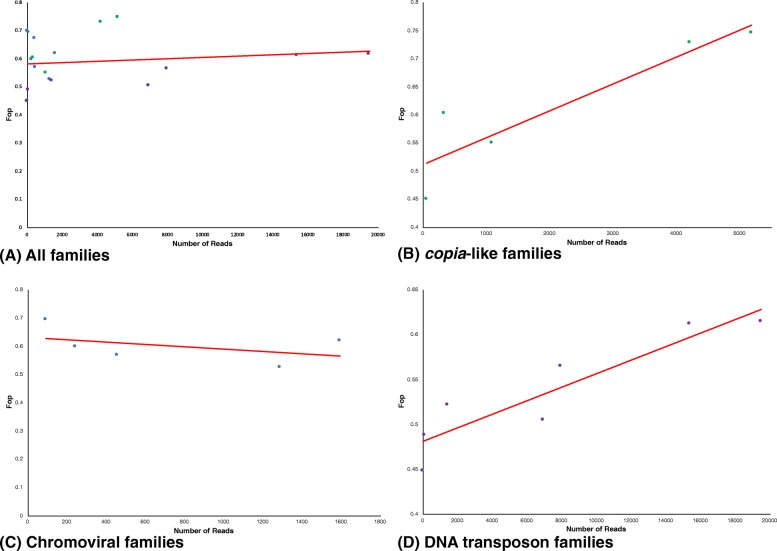
Values of F_op_ plotted against TE expression for TE families in the *S. rosetta* genome. **a** All TE families. **b** *copia-*like families. **c** Chromoviral families. **d** DNA transposon families. *Green dots* represent *copia*-like families, *blue dots* represent *gypsy*-like families and *purple dots* represent DNA transposons. The linear line of best fit is shown in red

The host genes in the *S. rosetta* genome show a clear, positive relationship between the use of optimal codons (F_op_) and gene expression, consistent with selection operating at the level of translational efficiency [29]. No relationship was recovered between Fop and the number of mapped SRA reads when all TE families were analysed together (*R*^2^ = 0.021, Fig. 6). However, when individually examined the *copia*-like and transposon families show a positive relationship between F_op_ and expression (*R*^2^ = 0.831 and *R*^2^ = 0.841 respectively), indicating that selection for translational efficiency operates on the TE genes. However no relationship was observed between expression level and F_op_ for the chromoviral families (*R*^2^ = 0.20). Furthermore, when the *copia*-like families and the transposon families, which individually showed evidence for selection at the level of translational efficiency, were analysed together no relationship was observed between F_op_ and expression level (*R*^2^ = 0.14, data not shown). This indicates that if there is competition between TE families, it does not operate between families of different TE classes.

Selection for rapid translation (translational efficiency) is likely to affect codons across entire transposable element genes; however, when selection is operating at the level of translational accuracy it can be expected that functional domains will show stronger selection than other regions of proteins [42]. Southworth et al. [29] showed that the signature of translational accuracy can be detected even in some of the most weakly biased genes within the *S. rosetta* genome. No functional domain could be identified in the ORF of *SrosS*, so investigating the signature of translational accuracy was not conducted in this family. F_op_ values were elevated in domain codons over non-domain codons in eight of the twelve LTR retrotransposon families and four out of the six investigated DNA transposon families (Table 4), consistent with selection on codons for translational accuracy. However, optimal codons were only significantly enriched in the domain regions of three families, *Srospv2*–*3* and *Srospv5*, all of which were *copia*-like LTR retrotransposons (*P* < 0.05, Fisher’s exact test, Table 4).

**Table 4:** Frequency of optimal codons (F_op_) in non-domain and domain regions of *S. rosetta* TE genes

Family	Non-domain F_op_	Domain F_op_	Significance (Fisher’s Exact Test)^a^
LTR Retrotransposon Families			
*Sroscv1*	0.565	0.576	NS
*Sroscv2*	0.591	0.609	NS
*Sroscv3*	0.559	0.472	0.002
*Sroscv4*	0.700	0.684	NS
*Sroscv5*	0.621	0.619	NS
*Srosgyp1*	0.684	0.725	NS
*Srosgyp2*	0.663	0.688	NS
*Srospv1*	0.593	0.619	NS
*Srospv2*	0.698	0.783	**< 0.001**
*Srospv3*	0.711	0.820	**< 0.0001**
*Srospv4*	0.457	0.438	NS
*Srospv5*	0.531	0.596	**< 0.03**
Transposon Families			
*SrosH*	0.608	0.636	NS
*SrosHar*	0.459	0.415	NS
*SrosM*	0.530	0.456	NS
*SrosTig1*	0.477	0.548	NS
*SrosTig2*	0.466	0.519	NS
*SrosTm*	0.590	0.649	NS

^a^ *P* values showing significantly elevated values of F_op_ in domain regions are highlighted in bold

### GC-codon usage bias is present in the *M. brevicollis* LTR retrotransposon families

The three LTR retrotransposon families of *M. brevicollis* [10] also exhibit a positive association between F_op_ and copy number (*R*^2^ = 0.663, Additional file 10), raising the possibility that selection on codon usage may be a phenomenon common to many choanoflagellates TEs.

Mean F_op_ values for the three *pol* ORFs were similar, albeit lower, than those of the LTR retrotransposon families of *S. rosetta* (0.579 ± 0.008), mirroring the weaker codon usage bias also observed in the host genes in *M. brevicollis* [29]. All three families possessed a majority of amino acids which preferred to use codons that either complemented the *M. brevicollis* major tRNA genes or were designated optimal codons [29] (Additional file 10). The current lack of expression data from *M. brevicollis* means that it is not possible to determine if selection for translational efficiency contributes to the observed relationship between copy number and F_op_.

The three LTR retrotransposon families in *M. brevicollis* show elevated, significantly so in the case of *Mbpv1*, values of F_op_ in functional domain codons compared to non-domain codons (Additional file 10), consistent with selection operating on translational accuracy. Mutation pressure towards guanosine and cytosine appears to play a role in codon usage bias in the LTR retrotransposons, as non-coding GC content shows a positive relationship with GC3s (*R*^2^ = 0.958, Additional file 10). It therefore appears that the codon usage of the *pol* ORFs of *M. brevicollis* LTR retrotransposons is determined by a combination of selection for optimal codons and mutation pressure.

Much of our current understanding of TE biology comes from *Drosophila melanogaster* and *S. cerevisiae* and the families from both species have been reported to show no evidence for selection on codon usage [31]. Mean F_op_ values, generated using species-specific optimal codons, of a sample of 26 LTR retrotransposon *pol* ORFs from *D. melanogaster* was 0.298 ± 0.067, whilst the four active families from *S. cerevisiae* had a mean F_op_ value of 0.444 ± 0.014 (Additional file 11). Accordingly, values of *N*_*c*_ for TE ORFs were also higher in both organisms when compared to those from *S. rosetta* (Table 3, Additional file 11). The data presented here show that the TEs of *S. rosetta* and *M. brevicollis* have evolved to utilize their host translation machinery more efficiently than either of the model organisms, *D. melanogaster* and *S. cerevisiae*, previously used extensively to study TE biology.

## Discussion

### TE family diversity in the *S. rosetta* genome

The genome of *S. rosetta* contains a far wider repertoire of elements than *M. brevicollis*, with a minimum of 20 TE families, from both Class I and Class II uncovered. The diversity of families is similar to that in *C. owczarzaki*, which harbours 23 families from both TE classes [4]. It is clear that unicellular eukaryotes are highly variable in terms of the diversity of TE families present. Species such as *S. cerevisiae* [39], *M. brevicollis* [10] and the alveolate *Plasmodium falciparum* [5] harbour a limited number of families, with fewer than five active families present in each species, whereas *S. rosetta*, *C. owczarzaki* [4] and *T. vaginalis* [43] all possess in excess of 20 families. Changes in TE diversity may evolve rapidly within eukaryotic lineages. In addition to the contrasting TE complements of the two craspedid choanoflagellates presented here, previous studies have highlighted large variations in TE family diversity in *Plasmodium* species and microsporidian fungi [5, 44].

The TEs in *C. owczarzaki* all had closely related orthologous families in other opisthokonts, highlighting a putatively diverse TE complement present in the last common ancestor of Opisthokonta. The TEs of *S. rosetta* show a more complex evolutionary history however, with most families appearing to be vertically inherited since the origin of opisthokonts whilst *SrosTm* appears to have been acquired horizontally from a stramenopile donor. Horizontal transfer is a well documented phenomenon in choanoflagellates [45, 46], with donor species, such as bacteria and small unicellular eukaryotes, being prey items of choanoflagellates [16]. The close proximity of the nucleus to food vacuoles within the cell may facilitate the passage of foreign DNA between organelles and its integration into the choanoflagellate chromosomes. Transferred genes must confer a selective advantage to their recipient in order to be retained, however the replicative, selfish nature of TEs may allow invading families to proliferate in a naïve genome in the face of negative selection. The presence of a *SrosTm* orthologue, albeit a putatively non-functional one, in the *M. brevicollis* genome indicates that the transfer of this transposon family was an ancient one. The family has continued to successfully transpose in *S. rosetta*, however the *M. brevicollis* family now appears to have lost function.

The *copia*-like family from *St. diplocostata*, which clusters phylogenetically with homologous families in both *S. rosetta* and *M. brevicollis*, highlights the antiquity of *copia*-like retrotransposons within choanoflagellates. The monophyly of choanoflagellate *copia*-like elements, from both craspedid and acanthoecid species, strongly points to this form of TE being present in the ancestor of the choanoflagellates. The vertical inheritance of *copia*-like elements since the LCA of opisthokonts is also suggested here as the closest relatives of the choanoflagellate elements are predominantly present in opisthokont genomes.

### TE activity in the *S. rosetta* genome

Similar to the TE families found in *C. owczaraki* [4], as well as the more distantly related *S. cerevisiae* and *T. vaginalis* [39, 43], the families in the *S. rosetta* genome show evidence for both current or recent activity. With the exception of *SrosHar*, which only had a single 20 bp SRA read mapped to it, all of the families, including the non-functional *Srospv6*, are expressed. Family phylogenies of individual insertions present sequences on short terminal branches, consistent with recent transposition events. The LTR sequences of both the *copia*-like and *gypsy*-like families show high (> 99%) intra-element identity (Table 1), further highlighting the recent activity of all retrotransposon families. One notable difference between the Class I and Class II families was the abundance of identical paralogous sequences in the former compared to the latter (Tables 1 and 2), with only *SrosM* and *SrosTm* of the transposons possessing identical copies at different locations. As identical copies are generated through transposition, this finding is consistent with a lower transposition rate for transposons in comparison to LTR retrotransposons, however this cannot be confirmed due to the current lack of information on direct transposition rates.

Further similarity can be drawn with *C. owczarzaki* and *S. cerevisiae*, in that all full-length LTR retrotransposons are presented on short branch lengths within phylogenies, and therefore can be presumed to be young elements [4, 39]. This finding supports the rapid elimination of full-length LTR retrotransposons, either through LTR recombination or their loss as a result of selection. In both the DNA transposon and retrotransposon families the ancient copies are mainly non-functional, as they are truncated and partial sequences, which can no longer transpose.

The TEs found in *S. rosetta*, *M. brevicollis* and *C. owczarzaki* all have low copy number (Tables 1 and 2, [4, 10]), with only one family, *Srospv3*, found to harbour over 100 copies. Many protist species possess large effective population sizes [47] and this may facilitate the efficient elimination of individual deleterious TE insertions. An alternative, although not mutually exclusive, explanation may be that the holozoan protists are under strong selection pressure for compact genomes, to enable rapid cell division and therefore reproduction, which would favour individuals with lower numbers of TE insertions.

### The TE families of *S. rosetta* show evidence for selection on their codon usage

Past studies on codon usage within TE ORFs have highlighted a consistent pattern of a weak bias toward AT-ending codons. This trend has been observed within both classes of TE in a broad range of host species ([30–33], Additional file 11). None of the TE families within the *S. rosetta* genome follow the commonly reported TE trend of AT-bias within their codon usage. In contrast, all families are GC-rich at synonymous third positions, with mean GC3s being greater than 0.60 for both Class I and Class II families (Table 3). The mean non-coding GC content of both classes is close to 0.50 (Table 3), which does not point to mutation pressure driving neutral base composition to guanosine and cytosine. Furthermore, each family shows a preference for translationally optimal codons, as defined by the *S. rosetta* host genes [29], in the majority of their encoded amino acids, with a preference for codons which complement the products of the major tRNA genes observed for most amino acids in most families (Table 3 and Additional file 8).

Unlike the *S. rosetta* host genes, where no clear evidence for mutation pressure was uncovered by Southworth et al. [29], the forces controlling codon usage within the TE families appear to be more complex. Both chromoviruses and the *copia*-like families show a positive relationship between GC content at non-coding DNA and synonymous third codon positions, indicating that mutation pressure towards GC makes a contribution to their codon usage bias. The non-chromoviral *gypsy*-like families and transposon families however failed to show a positive relationship between GC3s and non-coding GC content. In sharp contrast to the findings of past studies, the *copia*-like and transposon families of *S. rosetta* show clear evidence for selection for translational efficiency, with the *pol* and *tnpase* genes of highly expressed families being enriched for optimal codons in comparison to weakly expressed families. In addition to selection for translational efficiency, three of the *copia*-like families also exhibited significantly elevated use of optimal codons in functional domain regions of their *pol* ORFs when compared to the inter-domain codons, indicating that selection for translational accuracy also drives their codon usage. Non-significant enrichment of optimal codons in functional domain regions of genes was also observed in five other LTR retrotransposon families as well as four of the six tested transposon families, raising the possibility that this is a mechanism that influences codon usage patterns in the majority of TE families with the *S. rosetta* genome.

The LTR retrotransposon families have, on average, higher values of both GC3s and F_op_ than the DNA transposon families (Table 3). As noted earlier, the evidence from identical paralogous sequences indicates that the retrotransposons may be more active than the transposons. Selection is mainly expected to occur on TE sequences during transposition, since individual copies are, in effect, pseudogenes with respect to their host and expected to evolve neutrally as they accumulate random mutations. The higher use of optimal codons observed in retrotransposons may therefore be a product of their putatively higher transposition rate.

The presented evidence strongly points toward selection, for translationally optimal codons, playing an important role in the codon usage of the identified TE families in two species of choanoflagellate. The possession of a higher enrichment of translationally optimal codons is likely to provide a selective advantage between copies of the same family. Following Brookfield’s analogy of viewing the genome as an ecological community [37], it can also be speculated that increased use of optimal codons will allow some families to outcompete other families within a species’ genome. If “resources”, such as integration sites in a compact genome or interactions with host proteins to facilitate transposition, are limited, then families enriched for optimal codons may be able to synthesize Pol and Tnpase enzymes more rapidly and have an increased transposition rate and therefore a competitive advantage. Consistent with this, *Srospv2* and *Srospv3* have the highest F_op_ values of all TE families in the *S. rosetta* genome and also have the highest overall copy number, as well as the highest number of identical paralogous copies (Tables 1, 2 and 3).

The seven *gypsy*-like families (*Sroscv1–5* and *Srosgyp1–2*) exhibit similar values of F_op_ to the five *copia-*like families (Table 3); the *gypsy*-like families also exhibit a preference for codons that complement the products of major tRNA genes (Additional file 8). However, unlike the *copia-*like families, they do not show any strong signature for selection operating upon codon usage at the level of translational accuracy or efficiency. One possibility is that the presence of highly repetitive regions in some *gypsy*-like families has resulted in inaccurate read values, thereby obscuring any putative evidence for selection at the level of translational efficiency. It may also be possible that the *gypsy*-like families are no longer evolving under selection for their codon usage and the use of optimal codons observed reflects the signature of past selection that has not had time to be erased from extant element copies. Long range sequencing reads, which span entire TE insertions, would allow patterns of nucleotide variation at synonymous and non-synonymous sites to be determined within copies of the same family, potentially highlighting the presence or absence of on-going selection on codon usage. However, choanoflagellate genome sequencing has yet to be undertaken with long read technologies, preventing a more in-depth exploration of on-going selection on codon usage.

Further work, on a broader range of unicellular eukaryotes, is required to determine if selection on TE codon usage is present outside of the choanoflagellates. The work of Jiang and Govers [34] on *Phytophthora gypsy*-like families mirrors the results here, with evidence consistent with selection on codon usage and stronger bias observed in high copy number families. The *Phytopthora* study did not however determine whether the codons preferentially utilized by high copy TE families were host optimal codons and also did not assess the role of mutation bias in TE codon usage. As unicellular species make up the majority of eukaryotic diversity [48], the past emphasis on multicellular species for TE research may have resulted in a clouded picture of how TE codon usage evolves within most eukaryotes. It is however clear that not all unicellular eukaryotes possess TEs that show selection on their codon usage, as no evidence for selection was found in the ORFs of the *Ty* elements of *S. cerevisiae* [31]. The forces and host population biology that promote selection on TE codon usage may only be identified once the range of eukaryotes that exhibit such selection is identified. The transition to multicellularity in Metazoa may have resulted in a considerable reduction in *N*_*e*_ [49] and potentially also reduced the *N*_*e*_ of the TE families present in metazoan populations to a level where selection could no longer efficiently operate upon TE codon usage. Investigating TE codon usage at other unicellular/multicellular boundaries may allow the impact of host *N*_*e*_ upon TE codon usage to be evaluated.

## Conclusions

The choanoflagellate *S. rosetta* harbours a diverse complement of TEs, which are mainly orthologous to families present within metazoan genomes. One DNA transposon family however appears to have been acquired by choanoflagellates via an ancient horizontal transfer event from a stramenopile. All identified families appear to have been active recently, however the sequenced strain of *S. rosetta* possesses one family no longer capable of transposition. The TEs of *S. rosetta* and a second choanoflagellate *M. brevicollis* show the clear signature of natural selection operating upon their codon usage. In contrast to previous findings in multicellular organisms, which have failed to find evidence for selection acting upon TE codon usage, the choanoflagellate TEs appear to be evolving under selection for translational efficiency and accuracy. Patterns of codon usage bias differ between DNA transposon, *copia*-like and *gypsy*-like families, with transposons showing weaker bias than retrotransposons. The use of host gene optimal codons is greater in highly expressed families and appears to provide a competitive advantage for some families enriched for optimal codons.

## Methods

### Identification of TE families in the *S. rosetta* genome

The 154 genomic supercontigs of *S. rosetta* deposited in the Origins of Multicellularity Project at the Broad Institute (https://www.broadinstitute.org/scientific-community/data/origins-multicellularity) were screened for TE sequences using two methodologies. The supercontigs were screened with the Protein Based RepeatMasker server hosted by the Institute for Systems Biology (http://www.repeatmasker.org/cgi-bin/RepeatProteinMaskRequest). Successful hits (≤E-05) for *copia*-like and *gypsy*-like LTR retrotransposon families; non-LTR retrotransposon families and DNA transposon families were recovered. Successful nucleotide hits were then translated into conceptual amino acid sequences and subjected to BLASTp analysis in order to confirm their identity as TE proteins. A second approach used the BLAST protocol and query sequences of Carr et al. [10]. To identify putative MITE derivatives of DNA tranposon families, the supercontigs were screened with MITE-Hunter [35] and MITE Tracker [36]. Both programs were used with default parameters.

The RepeatMasker and BLAST hits failed to recover full-length TE sequences. The Carr et al. [10] protocol of increasing consensus sequence coverage using overlapping sequencing reads from the NCBI Trace Archive, in this case for “Proterospongia sp. Atcc 50818” allowed the generation of full-length consensus sequences for all families, with the exception of two non-LTR retrotransposons. Consensus sequences for all full-length families are provided in Additional Data File 1. Individual families were defined on the basis of the 80–80-80 rule [50], showing less than 80% similarity over 80% of an aligned sequence covering a minimum of 80 bp. For retrotransposons, similarity was determined between LTRs, whilst for DNA transposons similarity was calculated between 5’ITR/UTRs. *Srospv3A* and *Srospv3B* were classified as subfamilies, rather than distinct families, as their LTRs share over 98% identity at the terminal 160 bp of the 3′ ends.

The *St. diplocostata* transcriptome assembly of Marron et al. [51] was screened with the Protein Based RepeatMasker server to identify putative TE sequences. Six contigs were recovered as potential *copia*-like sequences (Additional file 6) and aligned in MAFFT on the EMBI EBI server with default parameters [52] to produce the amino acid sequence used in the phylogenetic analyses. The *St. diplocostata copia*-like nucleotide sequences were translated using EMBOSS Transeq tool [53].

### Phylogenetic analyses

Datasets for superfamily phylogenies were created for all of the *S. rosetta* families by using translated amino acid query sequences of Transposase for DNA transposons, and Pol for LTR retrotransposons. The *S. rosetta* TE nucleotide consensus sequences were translated using EMBOSS Transeq. Sequence similarity searches of whole genome sequences were performed with BLAST (tBLASTn and BLASTp) through NCBI to identify closely related TE families in a diverse taxonomic range of eukaryotes (Alveolata, Amoebozoa, Apusozoa, Breviatea, Centroheliozoa, Cryptophyta, Rhodophyta, Stramenopiles, Choanoflagellatea, Ichthyosporea; Metazoa (screens divided into Deuterostomia, Gnathostomulida, Platyhelminthes, Protostomia, Cnidaria, Ctenophora, Mesozoa, Placozoa, Porifera); Fungi (screens divided into Blastocladiomycota, Chytridiomycota, Cryptomycota, Ascomycota, Basidiomycota, Entomophthoromycota, Glomeromycota, Microsporida, Neocallimastigomycota); Archaeplastida: (screens divided into Chlorophyta, Mesostigmata).

Recovered amino acid sequences were aligned using MAFFT, along with the choanoflagellate TE sequences. Alignments were manually edited by eye to reduce indel regions. Bayesian inference phylogenies were constructed using a mixed amino acid model with MrBayes 3.2.6 on XSEDE [54] using the CIPRES Science Gateway server [55]. The MCMC analyses consisted of 5,000,000 generations, a sampling frequency of 1000, with a burnin value of 1250.

Maximum likelihood phylogenies were ran using raxmlGUI 1.5 beta [56]. The ML and thorough bootstrap analysis were performed, using 100 parsimony starting trees and 1000 bootstrap replicates, using the PROTCAT model with estimated amino acid frequencies. The amino acid substitution matrix used for each family was taken from the output of the mixed model analysis from MrBayes.

LTR and ITR insertions for each TE family were identified using *S. rosetta* 5′ query sequences. Terminal sequences were downloaded from NCBI Trace Archive against ‘Proterospongia’ with a threshold of e-05. LTR sequences were used as queries for the retrotransposon families; for DNA transposons, 300 bp of the ITR and UTR sequences were used as query sequences. TSDs were identified to distinguish individual insertions; if identical 5′ and 3′ TSDs from the Trace Archive were present, the two termini of the same element could be identified. Partially sequenced LTRs were excluded from further analyses, as their category of element could not be determined. LTRs adjacent to internal TE DNA were labelled as being from full-length elements and LTRs flanked only by genomic DNA were labelled as solo LTRs.

TE family ML trees were generated using the raxmlGUI from 100 starting parsimony trees, using the GTRCAT model and supported with 1000 bootstrap replicates. MrBayes analysis was employed using the protocol stated for the amino acid datasets, although the GTR + I + Γ nucleotide substitution model was used.

Levels of nucleotide diversity for each TE family were calculated, using values of Nei’s π [40], with DnaSP version 5. 10. 01 [57]. Values of π were calculated DNA transposon families by the analysis of 5′ ITR/UTR alignments, with a maximum length of 300 bp aligned in MAFFT. Values of π for LTR retrotransposon families were determined with individual alignments, generated with MAFFT, containing solo elements, individual FLEs and combined FLE and solo elements to produce All, FLE and Solo Phylip files.

### Determining TE family expression levels

Raw Illumina RNA-Seq transcriptome reads (NCBI SRA files SRX042046-SRX042054 and SRX3432761-SRX3432762–74.8 million reads) were downloaded from NCBI and mapped to the TE gene sequences through SMALT v. 0.2.6 (https://www.sanger.ac.uk/science/tools/smalt-0). The families *Srosgyp1*, *Sroscv2*, *Sroscv5* and *SrosS* contained repetitive regions in their full-length CDS, which were over-represented in comparison to other regions of the CDS in the SMALT output. As it is unclear if the repetitive regions are also present in non-TE regions of the *S. rosetta* genome they were excluded from the final estimation of TE gene expression (the edited sequences are shown in Additional file 12). The total number of reads for each family was calculated from the SMALT output SAM files in Tablet v.1.17.08.06 [58].

### Analysis of codon usage bias in transposable element families

For each TE family in the *S. rosetta* genome values of *Nc*, F_op_ and GC3s were calculated for all coding sequences using CodonW 1.4.4 [59]. The fop.coa file for *S. rosetta* was taken from Southworth et al. [29]. Codons overlapping multiple regions of the TE, e.g. with LTR sequences as well as the overlapping *gag pol* regions of *Sroscv4* and *Sroscv5*, were excluded from analyses. The GC content of non-coding DNA from each TE family was also determined in CodonW.

In order to assess any contribution of selection on translational accuracy, the codons which encode amino acids in functional domains were separated from those that encode non-domain amino acids into individual FASTA sequences. Domain regions were identified by analysing each Pol and Tnpase protein sequence in BLASTp. F_op_ values were then determined for the concatenated domain and concatenated non-domain codons in each family.

## Supplementary information

**Additional file 1. **Genomic organization of the 20 families of transposable element characterized in the *S. rosetta* genome. (A) *gypsy*-like LTR retrotransposons: Red boxes represent long terminal repeat sequences, dark green boxes represent *gag* open-reading frames (ORFs), dark blue boxes represent *pol* ORFs and light green boxes represent *gag* + *pol* polyprotein ORFs. Protein coding domains are indicated as follows: CCHC, RNA binding motif; CD, chromodomain; IN, Integrase; P, Protease; RT, Reverse Transcriptase. (B) *copia*-like LTR retrotransposons: The format follows that of Additional file 1A. (C) Transposons: Red boxes represent inverted terminal repeat sequences, green boxes represent *tnpase* exon sequences, and light blue boxes represent *tnpase* intron sequences. Protein coding domains are indicated as follows: D,D,E, aspartic acid and glutamic acid catalytic domain; HHLD, helix-turn-helix like domain; MULE, *Mutator-like element* Tnpase domain.

**Additional file 2. **Annotated sequences of the 20 families of transposable element characterized in the *S. rosetta* genome. The full-length sequences for each identified family are presented, along with putative open-reading frames, untranslated regions and flanking repeats. Conceptual translations of encoded proteins are provided.

**Additional file 3. **TE superfamily maximum likelihood protein phylogenies. All trees were constructed with RAxML using the PROTCAT model and estimated amino acid frequencies. Unless specified the WAG substitution matrix was used to generate the trees. A) Chromovirus phylogeny constructed with the RTREV substitution matrix, B) Non-chromoviral *gypsy*-like phylogeny, C) *SrosH* phylogeny constructed with the RTREV substitution matrix, D) *SrosHar*, E) *SrosM* phylogeny constructed with the substitution Blosum matrix, F) *SrosS* phylogeny, G) *SrosTig1* phylogeny, H) *SrosTig2* phylogeny, I) *SrosTm* phylogeny, including the conceptual translation of the *M. brevicollis* putative *tnpase* pseudogene. Values for mlBP and biPP are shown above and below the branches respectively. 100% mlBP and 1.00 biPP are both denoted by “*”. Values < 50% mlBP and < 0.70 biPP are denoted by “-”. Choanoflagellate proteins are written in light blue font. Metazoan proteins are written in dark blue, fungal proteins in brown, stramenopile proteins in orange, archaeplastid proteins in green, amoebozoan proteins in purple, bacterial proteins in grey and viral proteins in black font. The alignment used to create each phylogeny is presented in Additional file 6.

**Additional file 4. **Alignment of the *SrosTm* Tnpase with a putative Tnpase encoded in the *M. brevicollis* genome. MAFFT alignment of the *SrosTm* Tnpase and *M. brevicollis* XP_001743358 with the *M. brevicollis* predicted in-frame first intron is translated. “*” conserved amino acids, “:” conservative substitution, “.” semiconservative substitution, “” non-conservative substitution.

**Additional file 5. **Maximum likelihood nucleotide phylogenies of TE copies identified in the *S. rosetta* genome. A) *Sroscv1* LTR phylogeny, B) *Sroscv2* LTR phylogeny, C) *Sroscv3* LTR phylogeny, D) *Sroscv4* LTR phylogeny, E) *Sroscv5* LTR phylogeny, F) *Srosgyp1* LTR phylogeny, G) *Srosgyp2* LTR phylogeny, H) *Srospv1* LTR phylogeny, I) *Srospv2* LTR phylogeny, J) *Srospv3* LTR phylogeny, K) *Srospv4* LTR phylogeny, L) *Srospv5* LTR phylogeny, M) *SrosM* ITR/UTR phylogeny, N) *SrosTig1* ITR/UTR phylogeny, O) *SrosTig2* ITR/UTR phylogeny and P) *SrosTm* ITR/UTR phylogeny. OTU labels are the immediate flanking DNA of the insertion. In the retrotransposon phylogenies LTR sequences from putatively intact insertions are shown in blue and sequences from non-functional, truncated insertions are shown in red. All families were created with the GTRCAT model, using empirical base frequencies. The alignment used to create each phylogeny is presented in Additional file 6.

**Additional file 6. ***gypsy*-like, *copia*-like and DNA transposon alignments. All alignments used to create phylogenies and nucleotide diversity values are provided.

**Additional file 7. ***Nc* plots for *S. rosetta* host genes and TE genes. A) *S. rosetta* host genes. B) TE genes. The linear line of best fit is shown in red.

**Additional file 8. **Preferred codons for each amino acid in the *S. rosetta* TE families. Green font denotes a favoured codon which complements the product of the major tRNA gene for the amino acid [29]. Blue denotes a favoured codon which does not complements the product of the major tRNA gene for the amino acid, but is a host defined optimal codon [29]. Codons written in red do not complement major tRNA gene products and are not host optimal codons. Black font is used when there is no single favoured codon for the stated amino acid.

**Additional file 9. **Values of F_op_ plotted against copy number for TE families in the *S. rosetta* genome. A) All TE families, B) *copia*-like families, C) chromoviral families and D) DNA transposon families. The linear line of best fit is shown in red.

**Additional file 10. ***Monosiga brevicollis* TE codon usage data. Table 1. Preferred codons for each amino acid in the *M. brevicollis* TE families. Table 2. Frequency of optimal codons (F_op_) in non-domain and domain regions of *M. brevicollis* TE ORFs. Chart 1. Copy number plotted against F_op_ for the three *M. brevicollis* LTR retrotransposons families (copy numbers taken from Carr et al. [10]). Chart 2. GC3s plotted against non-coding GC-content for the three *M. brevicollis* LTR retrotransposons families. The linear lines of best fit are shown in red.

**Additional file 11.** Codon usage statistics for LTR retrotransposons ORFs in *Drosophila melanogaster* and *Saccharomyces cerevisiae*. Values of *Nc*, GC3s and F_op_ (determined using host-specific optimal codons) for 26 families of LTR retrotransposons from *D. melanogaster* and four families from *S. cerevisiae*.

**Additional file 12.** Edited TE ORFs used in SMALT analyses. The ORF sequences of the four families presented contained repetitive regions that were excluded when determining gene expression levels.

## Data Availability

The datasets used and/or analysed during the current study are available in Additional file 6 and from the corresponding author on reasonable request.

## Abbreviations

ATCC: American Type Culture Collection
biPP: Bayesian inference posterior probability
cds: coding sequence
FLE: Full-length element
F_op_: Frequency of optimal codons
GC3s: Guanine+Cytosine content at synonymous third codon positions
ITR: Inverted terminal repeat
LCA: last common ancestor
LTR: Long terminal repeat
MITE: Miniature inverted repeat transposable element
mlBP: maximum likelihood bootstrap percentage
*N*_*c*_: Effective number of codons
*N*_*e*_: Effective population size
ORF: Open reading frame
OTU: operational taxonomic unit
SRA: Sequence Read Archive
TE: transposable element
TSD: Target site duplication
UTR: Untranslated region
