# Supplementary material for: A genomic survey of transposable elements in the choanoflagellate Salpingoeca rosetta reveals selection on codon usage
Source: Mob DNA. 2019 Nov 23;10:44. doi: 10.1186/s13100-019-0189-9 (PMC6875170; doi:10.1186/s13100-019-0189-9)
Supplement: Supplementary file 1 — Additional file 1. Genomic organization of the 20 families of transposable element characterized in the S. rosetta genome. (A) gypsy-like LTR retrotransposons: Red boxes represent long terminal repeat sequences, dark green boxes represent gag open-reading frames (ORFs), dark blue boxes represent pol ORFs and light green boxes represent gag + pol polyprotein ORFs. Protein coding domains are indicated as follows: CCHC, RNA binding motif; CD, chromodomain; IN, Integrase; P, Protease; RT, Reverse Transcriptase. (B) copia-like LTR retrotransposons: The format follows that of Additional file 1A. (C) Transposons: Red boxes represent inverted terminal repeat sequences, green boxes represent tnpase exon sequences, and light blue boxes represent tnpase intron sequences. Protein coding domains are indicated as follows: D,D,E, aspartic acid and glutamic acid catalytic domain; HHLD, helix-turn-helix like domain; MULE, Mutator-like element Tnpase domain. [file 13100_2019_189_MOESM1_ESM.pdf]

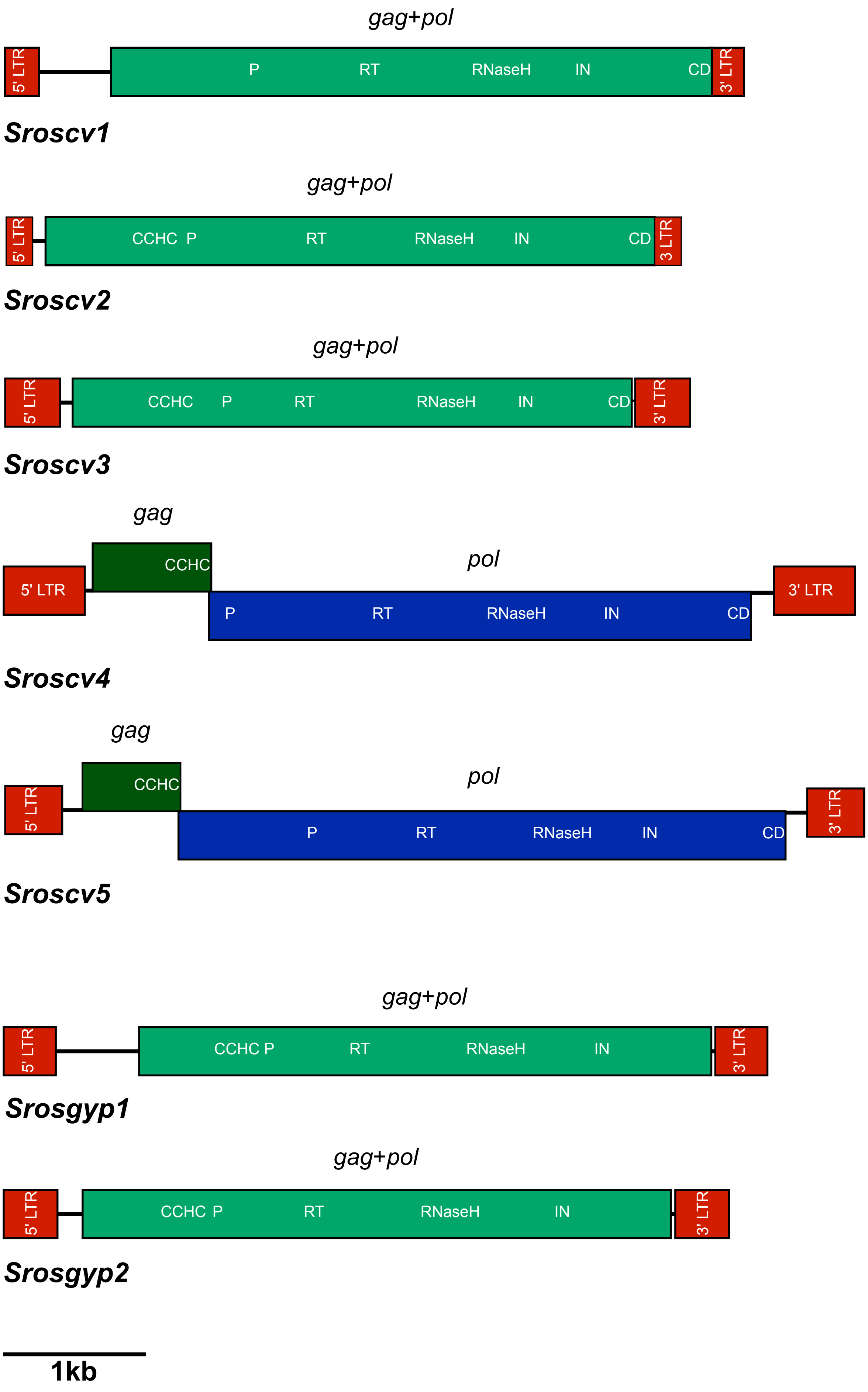

(A) *gypsy*-like families

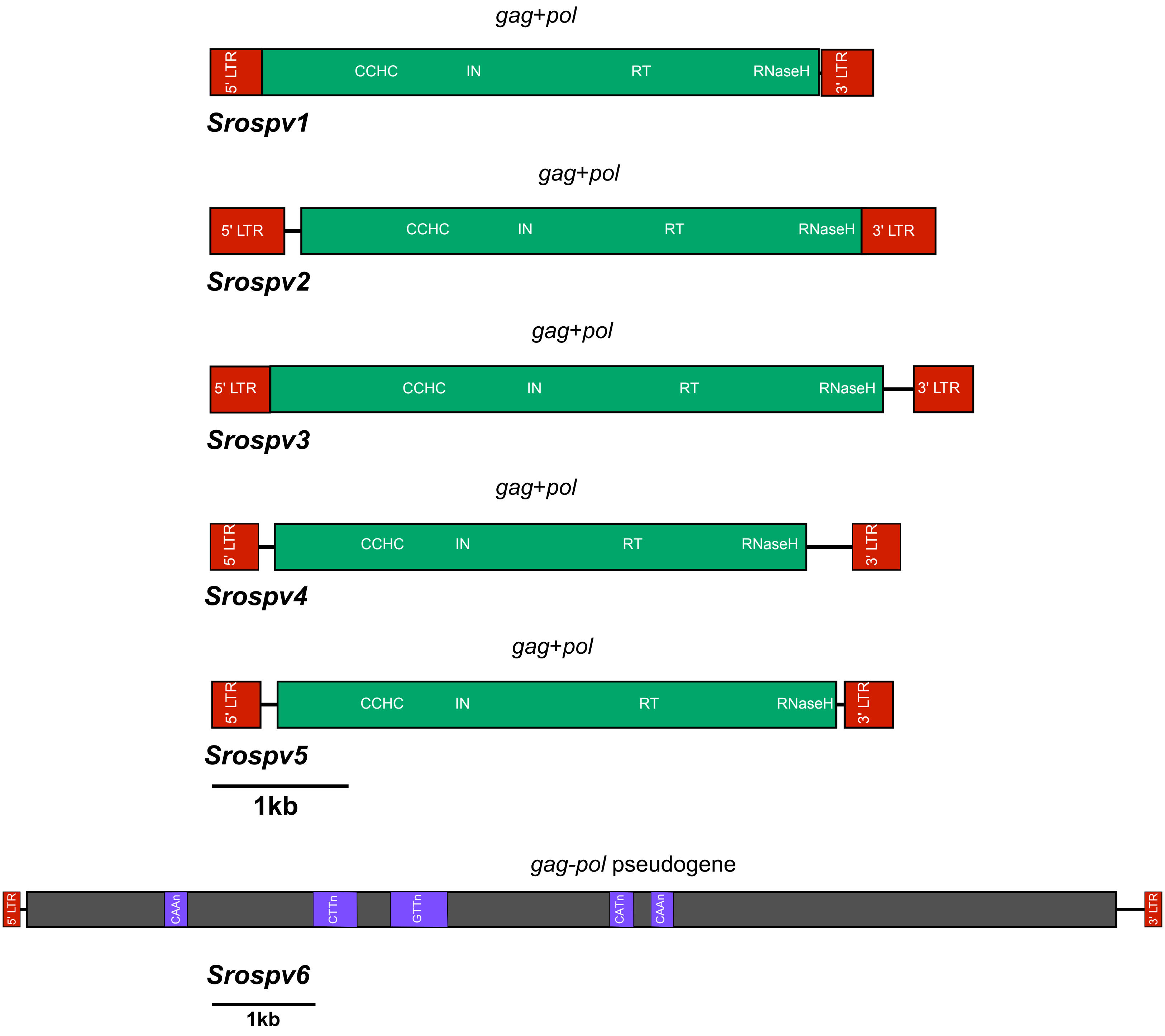

(B) *copia*-like families

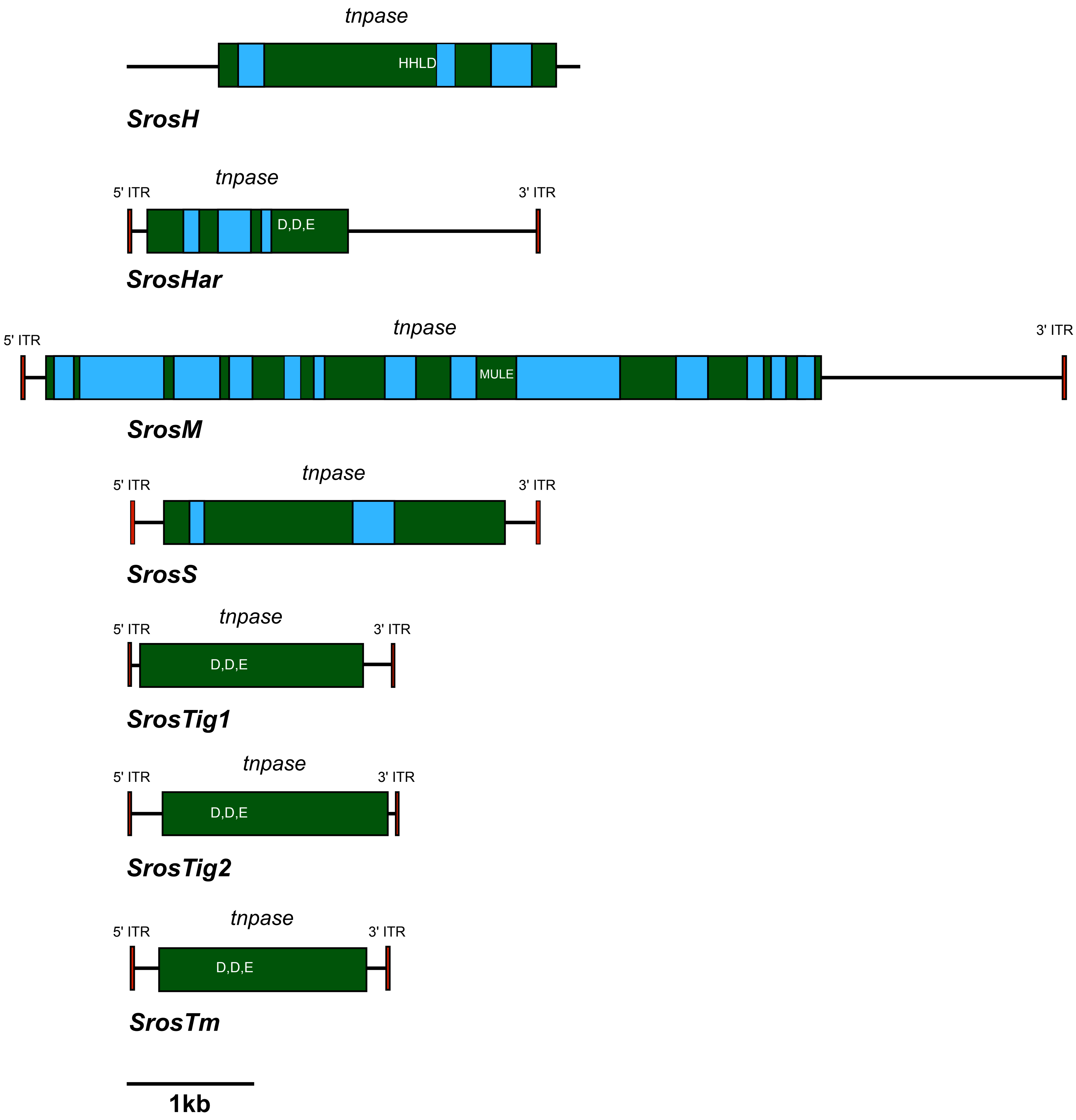

(C) Transposon families
