# Supplementary figures and images for: A genomic survey of transposable elements in the choanoflagellate Salpingoeca rosetta reveals selection on codon usage
Source: Mob DNA. 2019 Nov 23;10:44. doi: 10.1186/s13100-019-0189-9 (PMC6875170; doi:10.1186/s13100-019-0189-9)

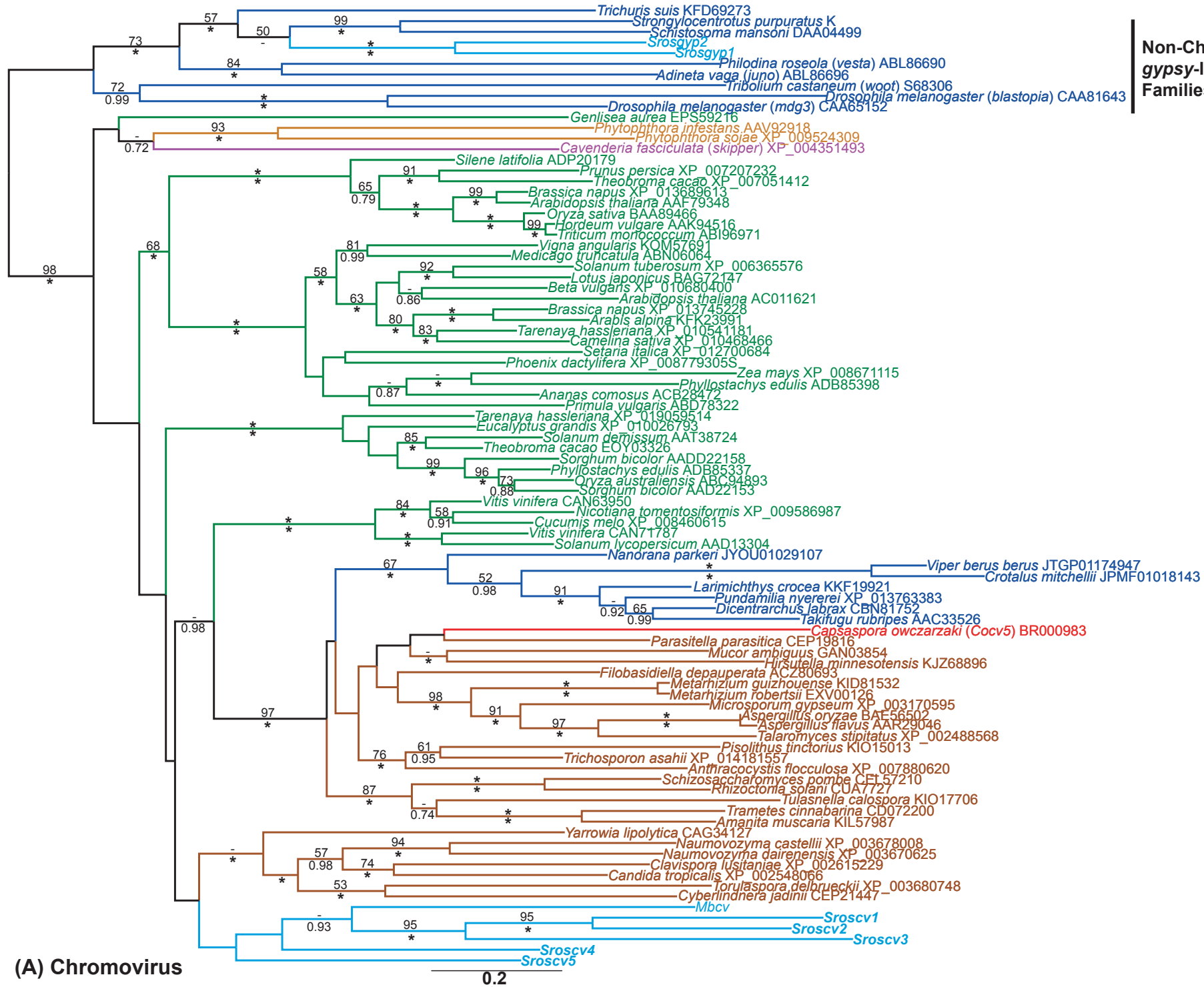

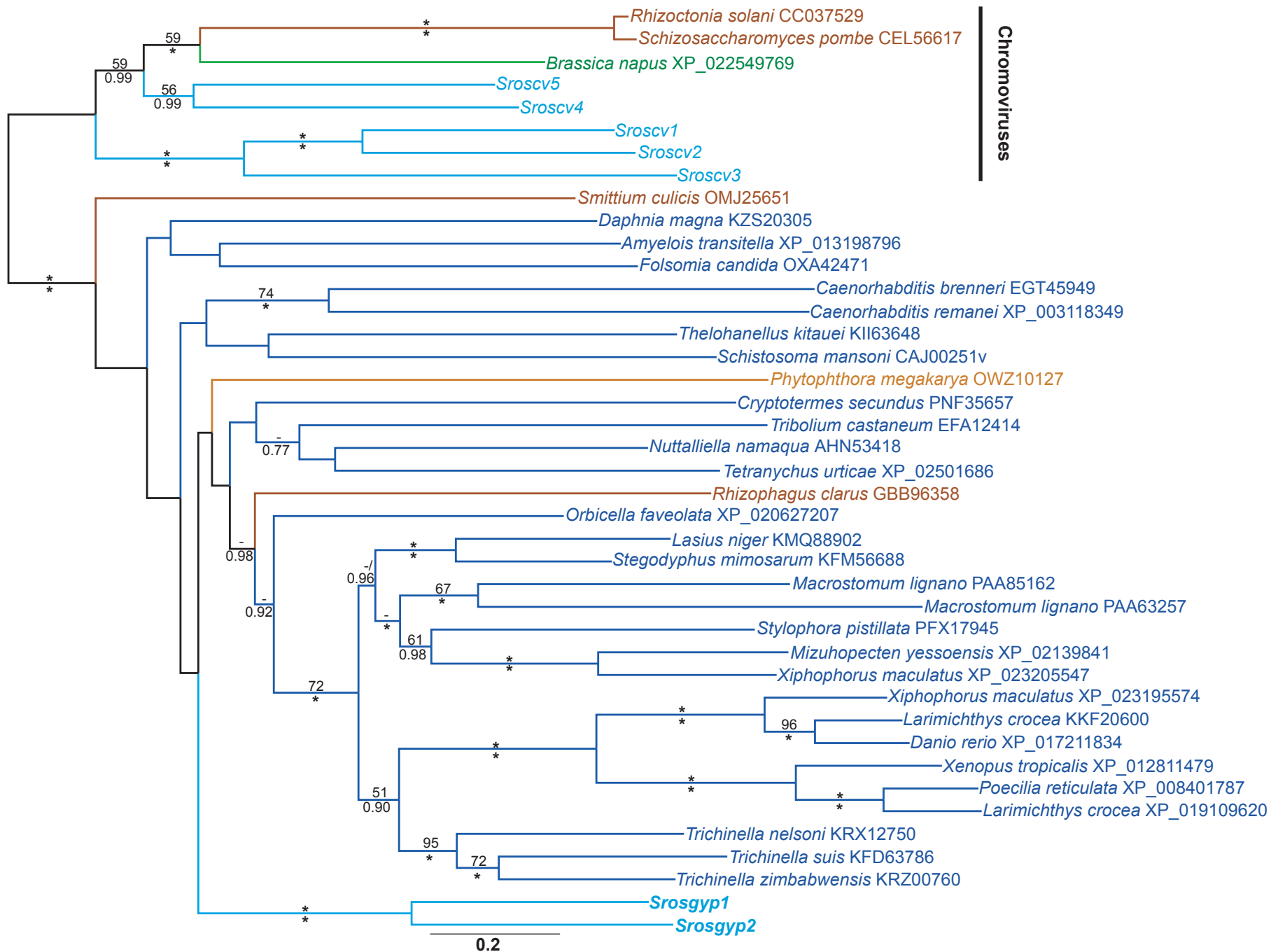

(B) Non-chromoviral gypsy-like families

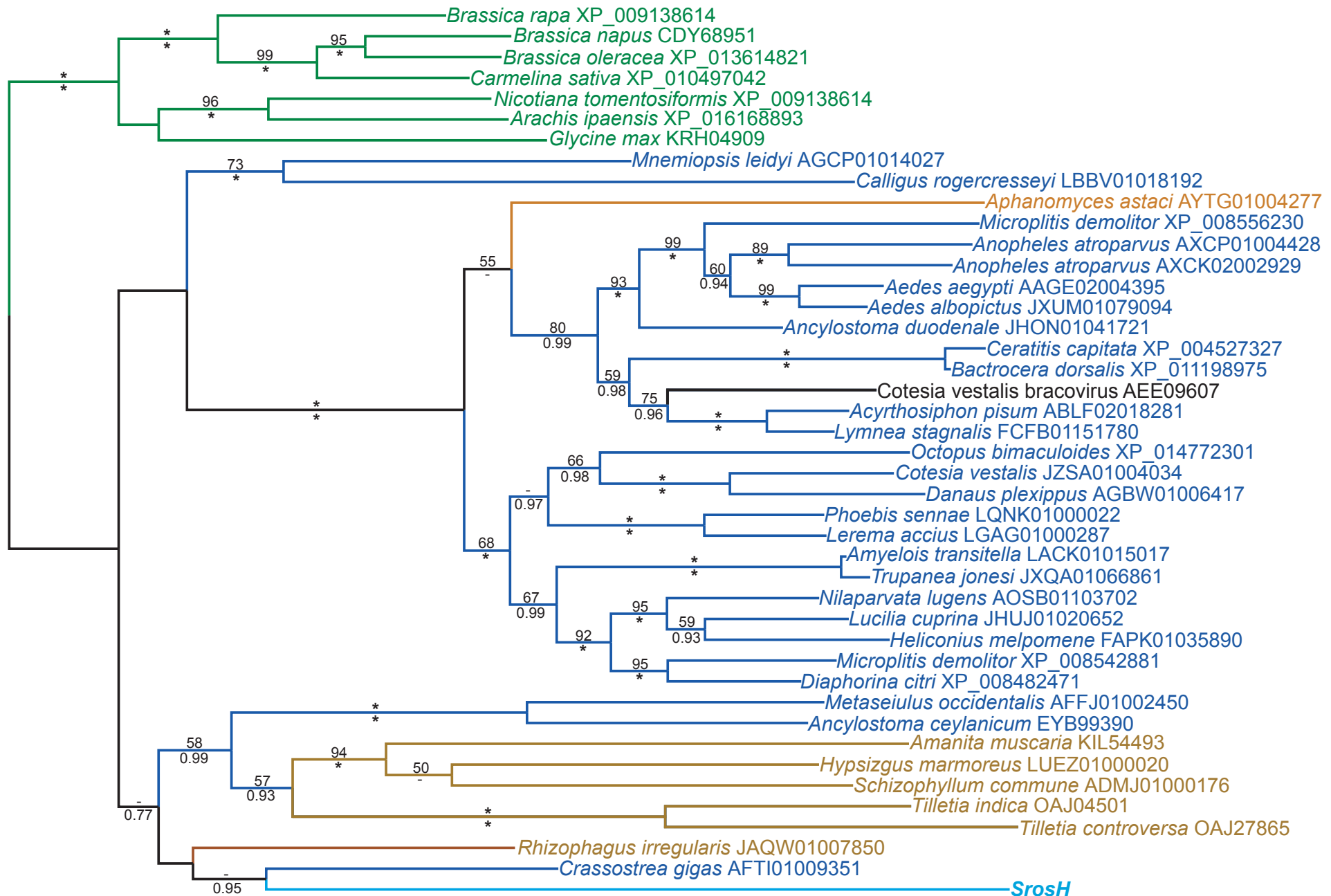

(C) *SrosH*

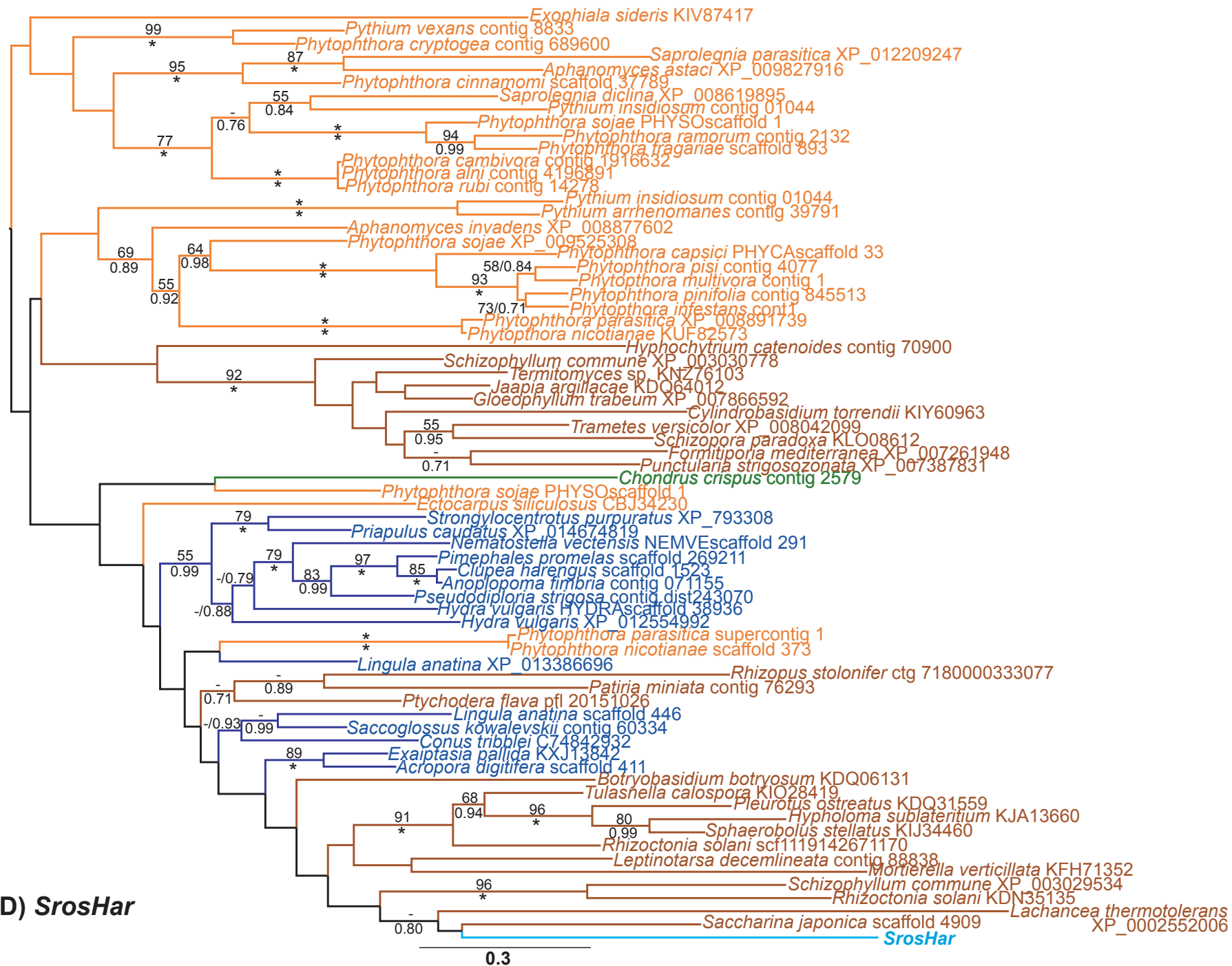

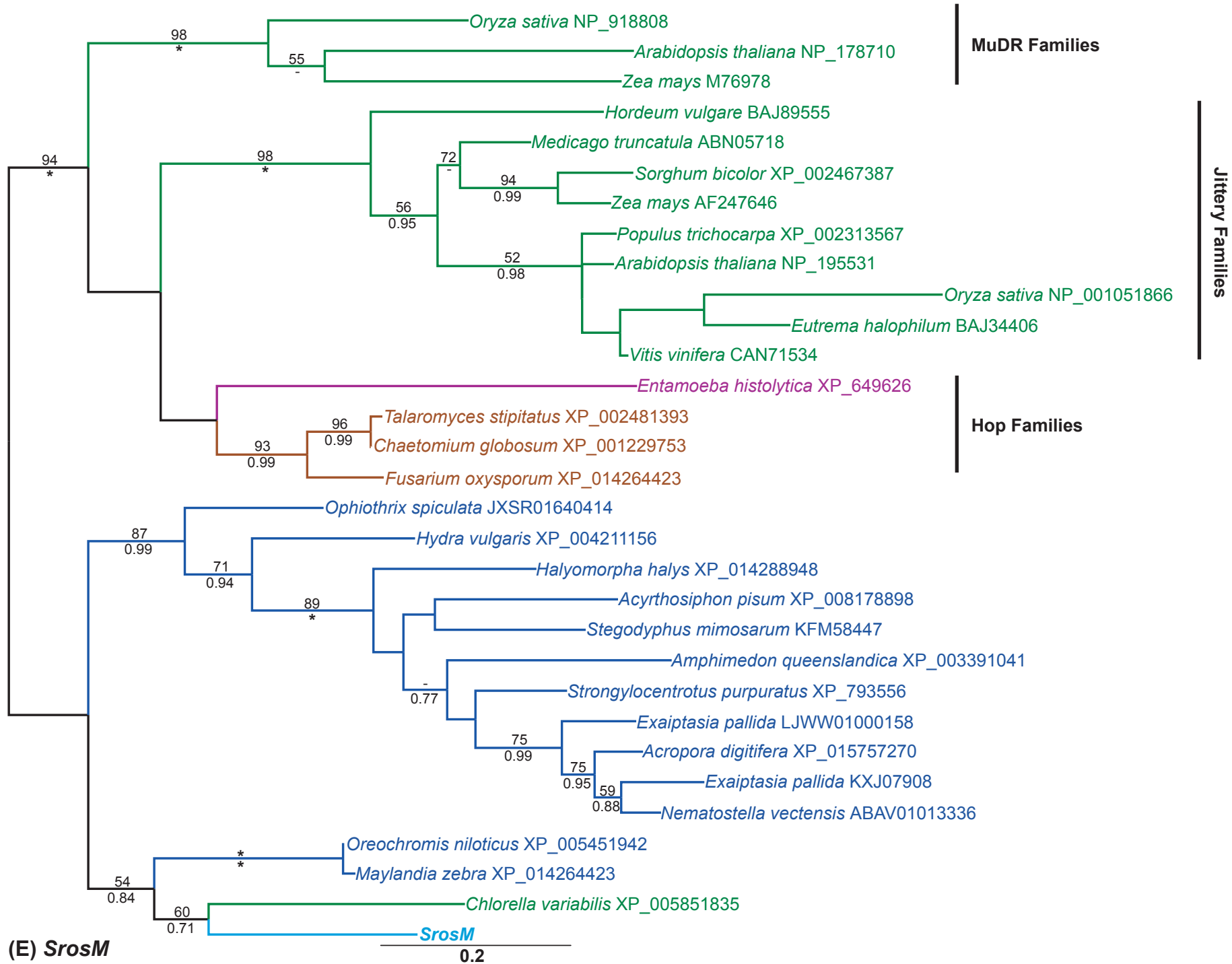

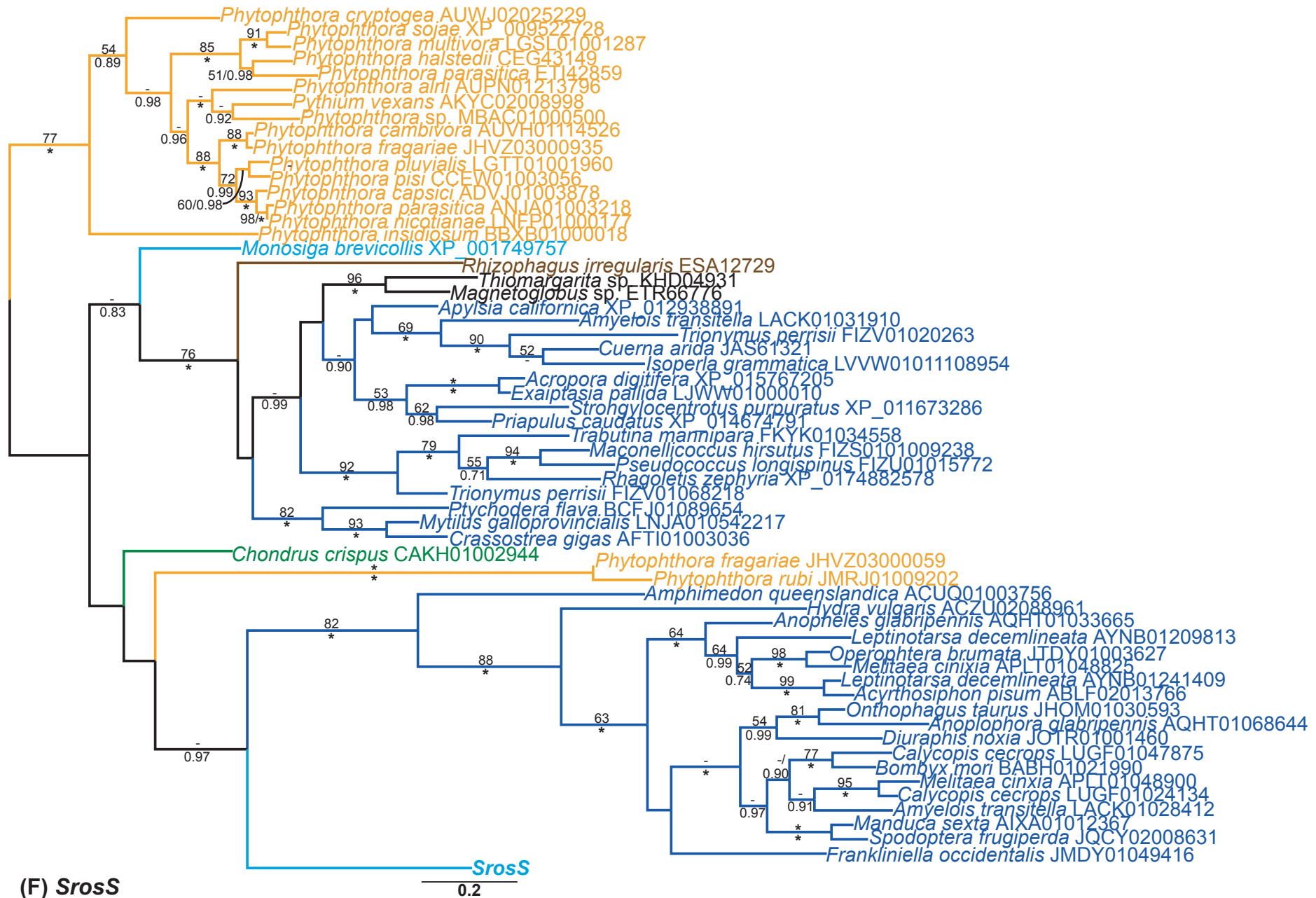

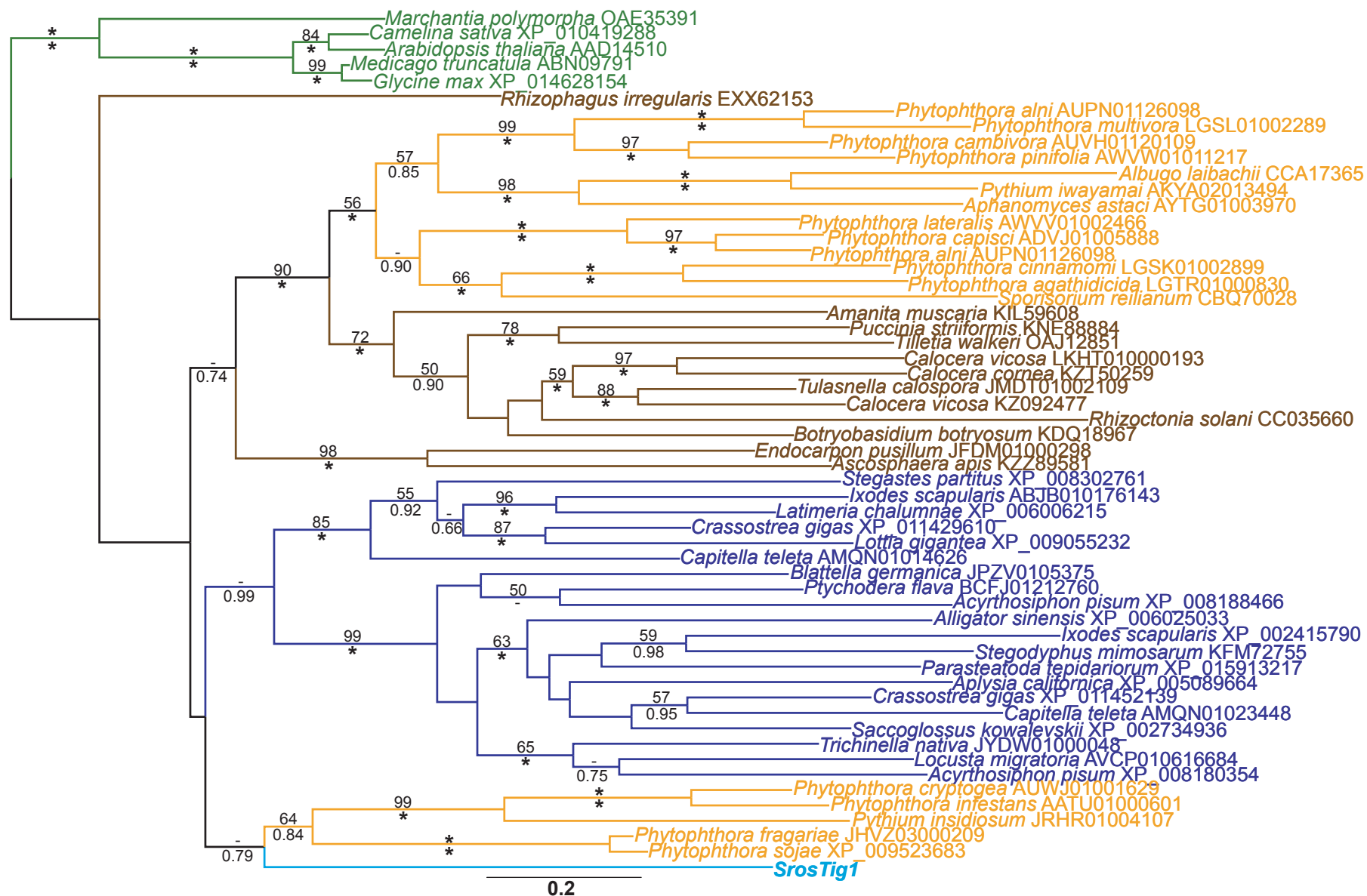

(G) *SrosTig1*

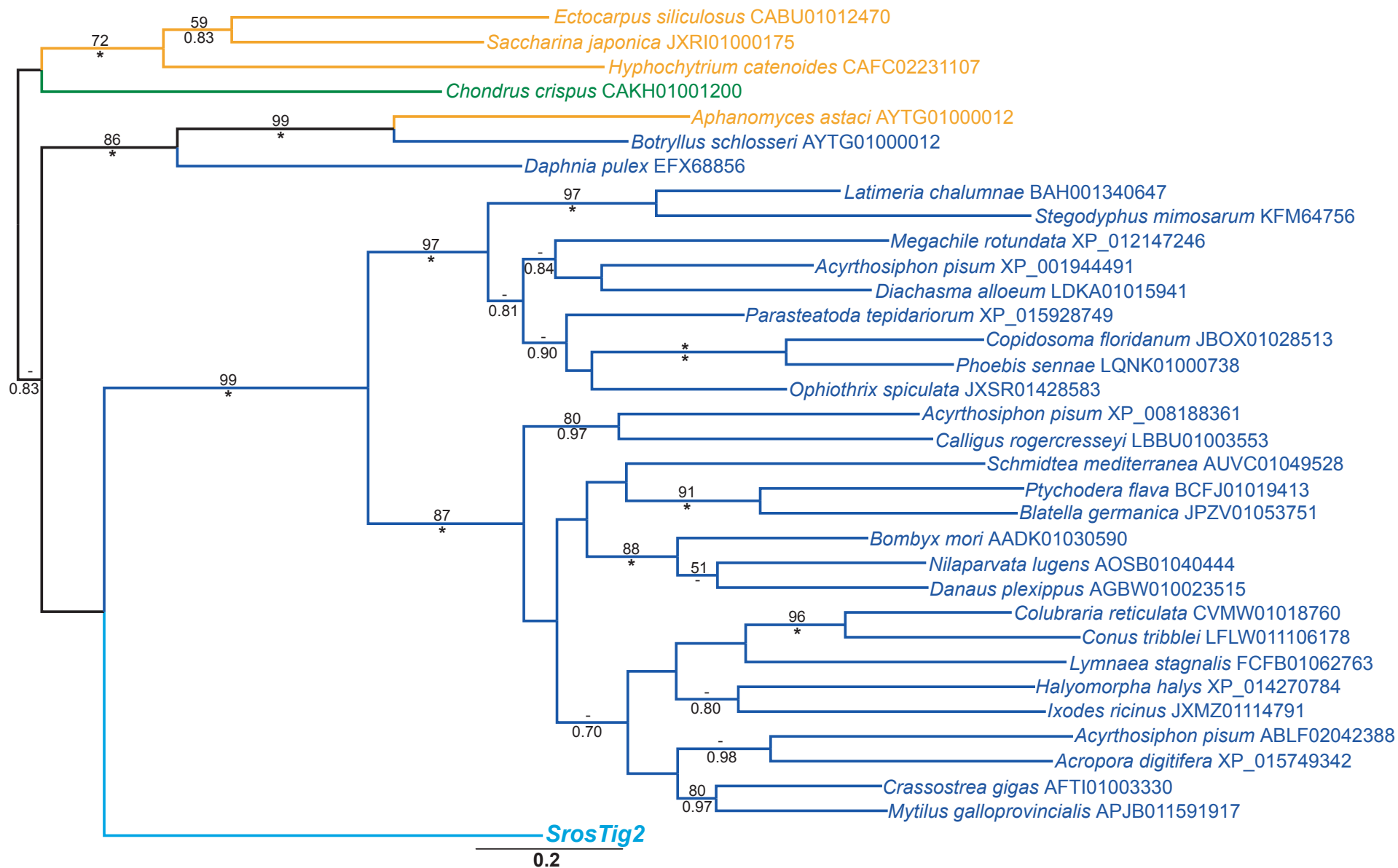

(H) SrosTig2

Supplement: Supplementary file 3 — Additional file 3. TE superfamily maximum likelihood protein phylogenies. All trees were constructed with RAxML using the PROTCAT model and estimated amino acid frequencies. Unless specified the WAG substitution matrix was used to generate the trees. A) Chromovirus phylogeny constructed with the RTREV substitution matrix, B) Non-chromoviral gypsy-like phylogeny, C) SrosH phylogeny constructed with the RTREV substitution matrix, D) SrosHar, E) SrosM phylogeny constructed with the substitution Blosum matrix, F) SrosS phylogeny, G) SrosTig1 phylogeny, H) SrosTig2 phylogeny, I) SrosTm phylogeny, including the conceptual translation of the M. brevicollis putative tnpase pseudogene. Values for mlBP and biPP are shown above and below the branches respectively. 100% mlBP and 1.00 biPP are both denoted by “*”. Values < 50% mlBP and < 0.70 biPP are denoted by “-”. Choanoflagellate proteins are written in light blue font. Metazoan proteins are written in dark blue, fungal proteins in brown, stramenopile proteins in orange, archaeplastid proteins in green, amoebozoan proteins in purple, bacterial proteins in grey and viral proteins in black font. The alignment used to create each phylogeny is presented in Additional file 6. [file 13100_2019_189_MOESM3_ESM.pdf]

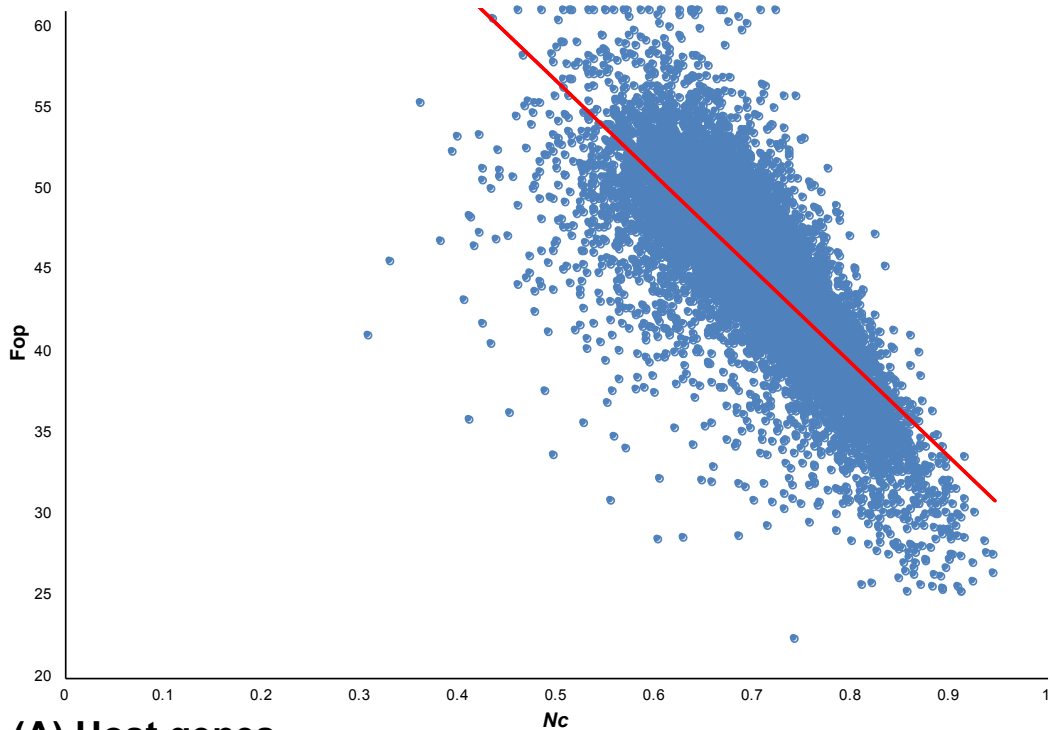

**(A) Host genes**

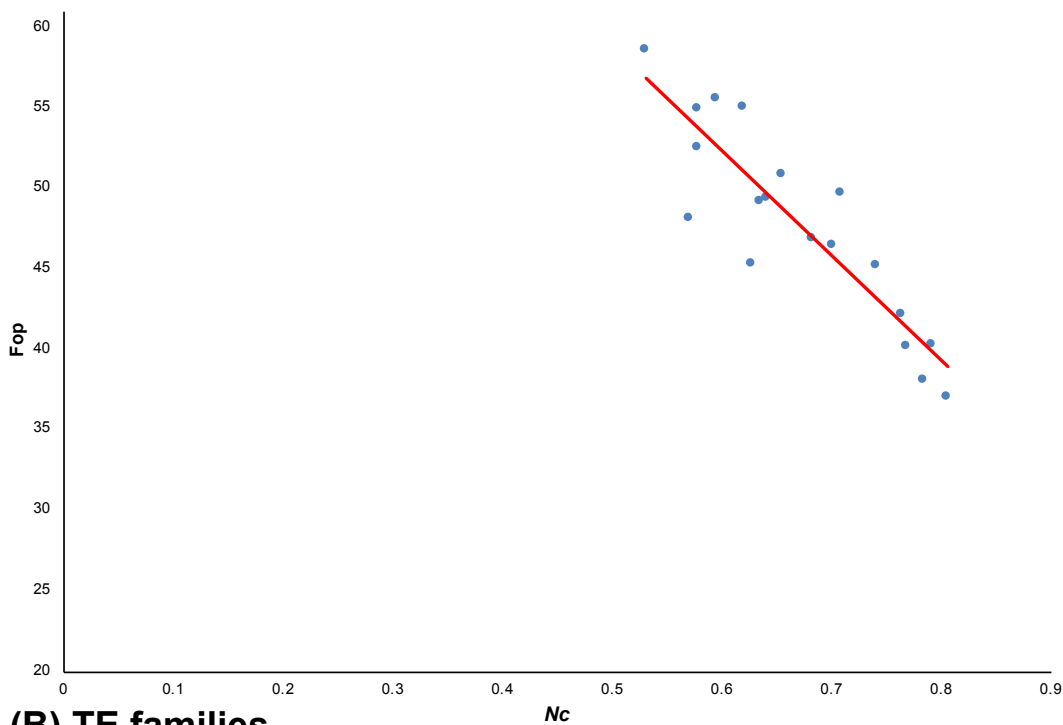

**(B) TE families**

Supplement: Supplementary file 7 — Additional file 7. Nc plots for S. rosetta host genes and TE genes. A) S. rosetta host genes. B) TE genes. The linear line of best fit is shown in red. [file 13100_2019_189_MOESM7_ESM.pdf]
