# Supplementary material for: A genomic survey of transposable elements in the choanoflagellate Salpingoeca rosetta reveals selection on codon usage
Source: Mob DNA. 2019 Nov 23;10:44. doi: 10.1186/s13100-019-0189-9 (PMC6875170; doi:10.1186/s13100-019-0189-9)
Supplement: Supplementary file 4 — Additional file 4. Alignment of the SrosTm Tnpase with a putative Tnpase encoded in the M. brevicollis genome. MAFFT alignment of the SrosTm Tnpase and M. brevicollis XP_001743358 with the M. brevicollis predicted in-frame first intron is translated. “*” conserved amino acids, “:” conservative substitution, “.” semiconservative substitution, “” non-conservative substitution. [file 13100_2019_189_MOESM4_ESM.pdf]

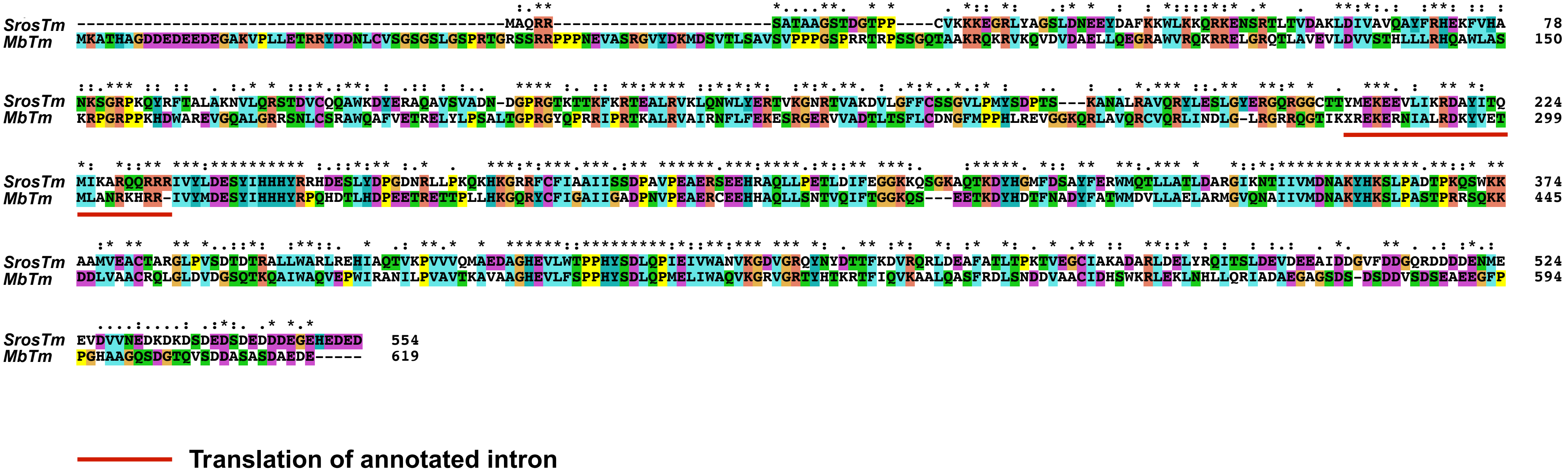
SrosTm
MbTm
78
150
224
299
374
445
524
594
554
619
Translation of annotated intron
