# Supplementary material for: A genomic survey of transposable elements in the choanoflagellate Salpingoeca rosetta reveals selection on codon usage
Source: Mob DNA. 2019 Nov 23;10:44. doi: 10.1186/s13100-019-0189-9 (PMC6875170; doi:10.1186/s13100-019-0189-9)
Supplement: Supplementary file 5 — Additional file 5. Maximum likelihood nucleotide phylogenies of TE copies identified in the S. rosetta genome. A) Sroscv1 LTR phylogeny, B) Sroscv2 LTR phylogeny, C) Sroscv3 LTR phylogeny, D) Sroscv4 LTR phylogeny, E) Sroscv5 LTR phylogeny, F) Srosgyp1 LTR phylogeny, G) Srosgyp2 LTR phylogeny, H) Srospv1 LTR phylogeny, I) Srospv2 LTR phylogeny, J) Srospv3 LTR phylogeny, K) Srospv4 LTR phylogeny, L) Srospv5 LTR phylogeny, M) SrosM ITR/UTR phylogeny, N) SrosTig1 ITR/UTR phylogeny, O) SrosTig2 ITR/UTR phylogeny and P) SrosTm ITR/UTR phylogeny. OTU labels are the immediate flanking DNA of the insertion. In the retrotransposon phylogenies LTR sequences from putatively intact insertions are shown in blue and sequences from non-functional, truncated insertions are shown in red. All families were created with the GTRCAT model, using empirical base frequencies. The alignment used to create each phylogeny is presented in Additional file 6. [file 13100_2019_189_MOESM5_ESM.pdf]

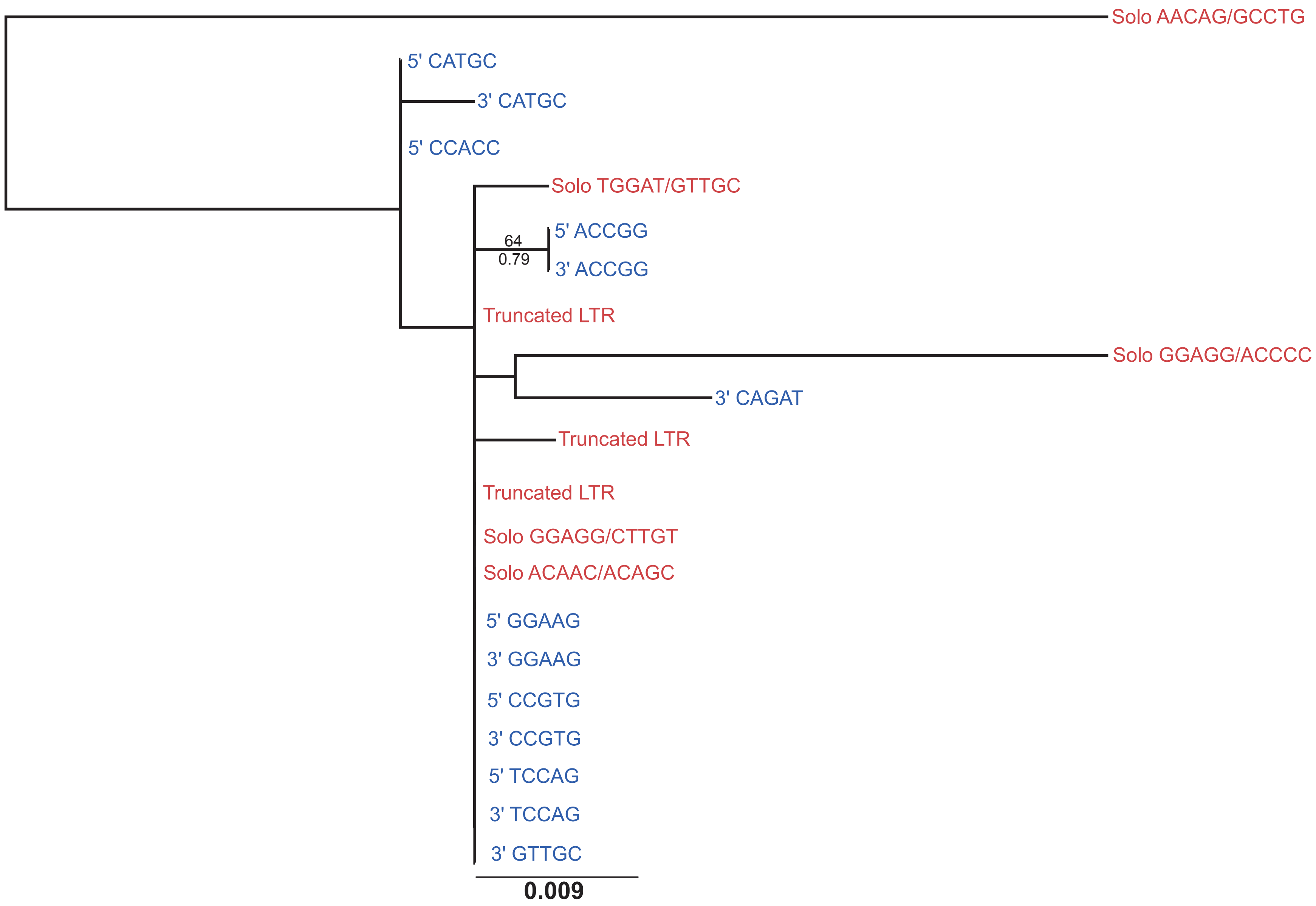

Solo AACAG/GCCTG
5' CATGC
3' CATGC
5' CCACC
Solo TGGAT/GTTGC
64
0.79
5' ACCGG
3' ACCGG
Truncated LTR
Solo GGAGG/ACCCC
3' CAGAT
Truncated LTR
Truncated LTR
Solo GGAGG/CTTGT
Solo ACAAC/ACAGC
5' GGAAG
3' GGAAG
5' CCGTG
3' CCGTG
5' TCCAG
3' TCCAG
3' GTTGC
0.009

(A) ***Sroscv1***

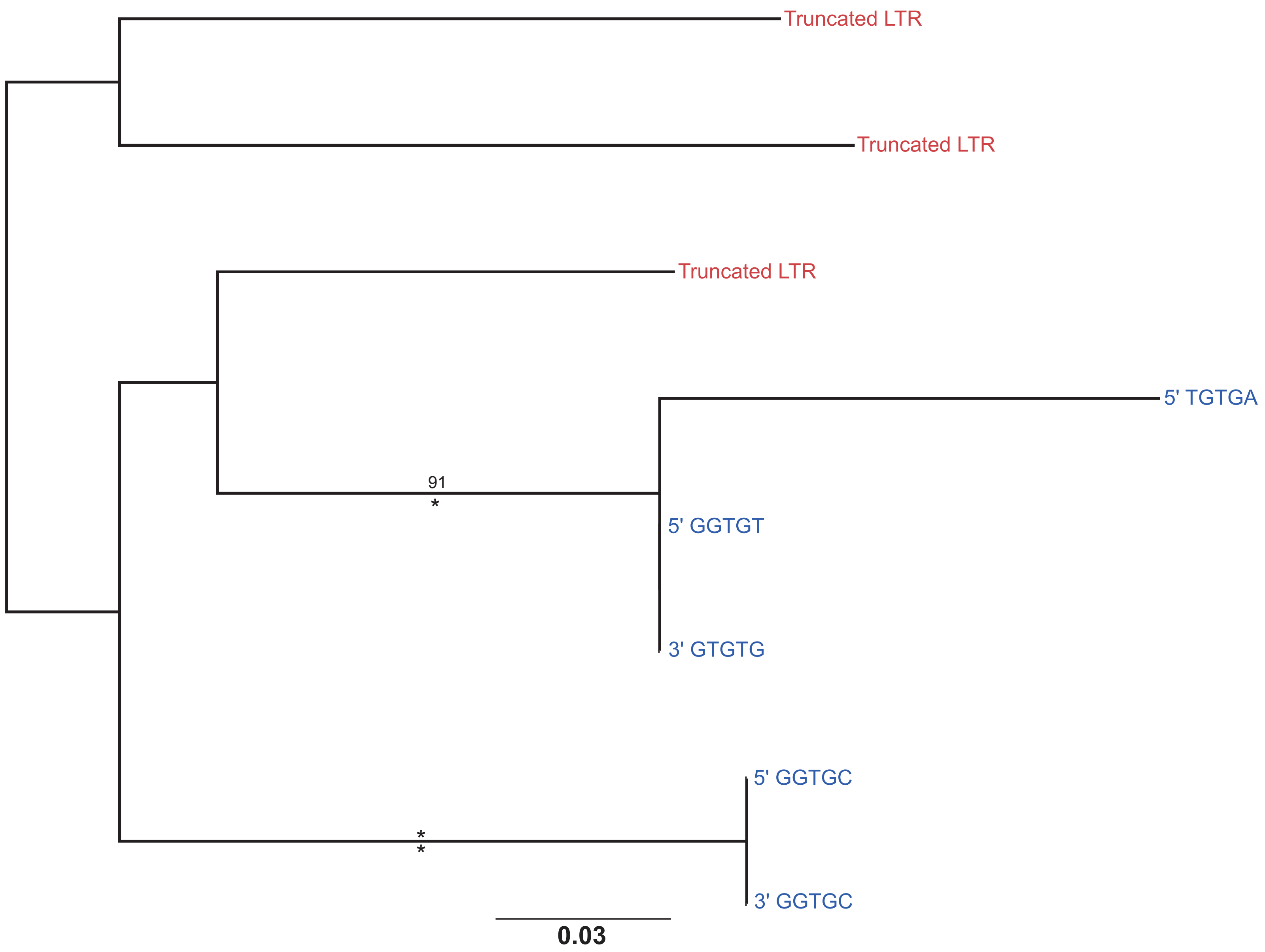
Truncated LTR
Truncated LTR
Truncated LTR
5' TGTGA
91
*
5' GGTGT
3' GTGTG
5' GGTGC
*
*
3' GGTGC
0.03

(B) ***Sroscv2***

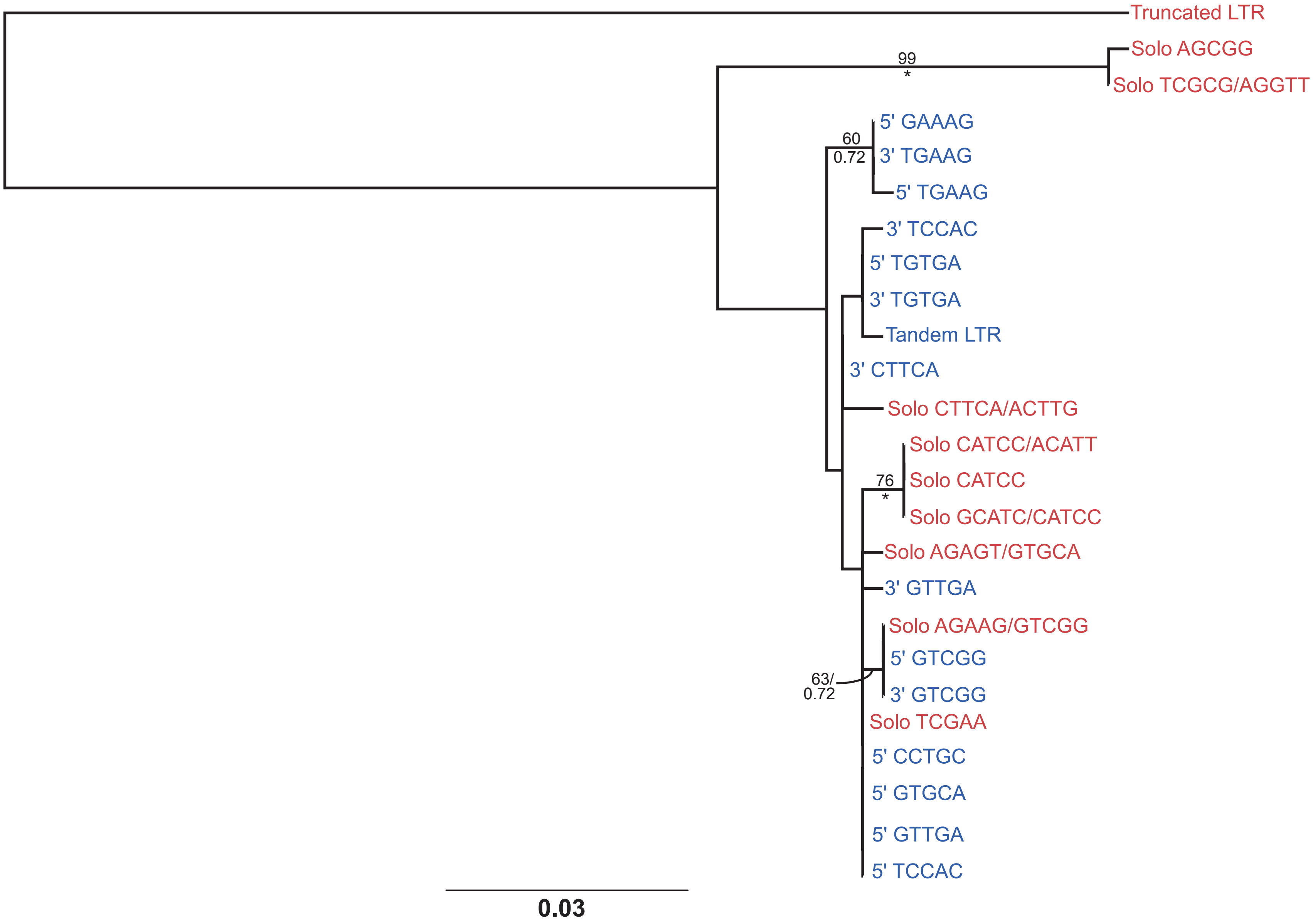
Truncated LTR
Solo AGCGG
99
*
Solo TCGCG/AGGTT
5' GAAAG
60
0.72
3' TGAAG
5' TGAAG
3' TCCAC
5' TGTGA
3' TGTGA
Tandem LTR
3' CTTCA
Solo CTTCA/ACTTG
Solo CATCC/ACATT
76
*
Solo CATCC
Solo GCATC/CATCC
Solo AGAGT/GTGCA
3' GTTGA
Solo AGAAG/GTCGG
5' GTCGG
63/
0.72
3' GTCGG
Solo TCGAA
5' CCTGC
5' GTGCA
5' GTTGA
5' TCCAC
0.03

(C) *Sroscv3*

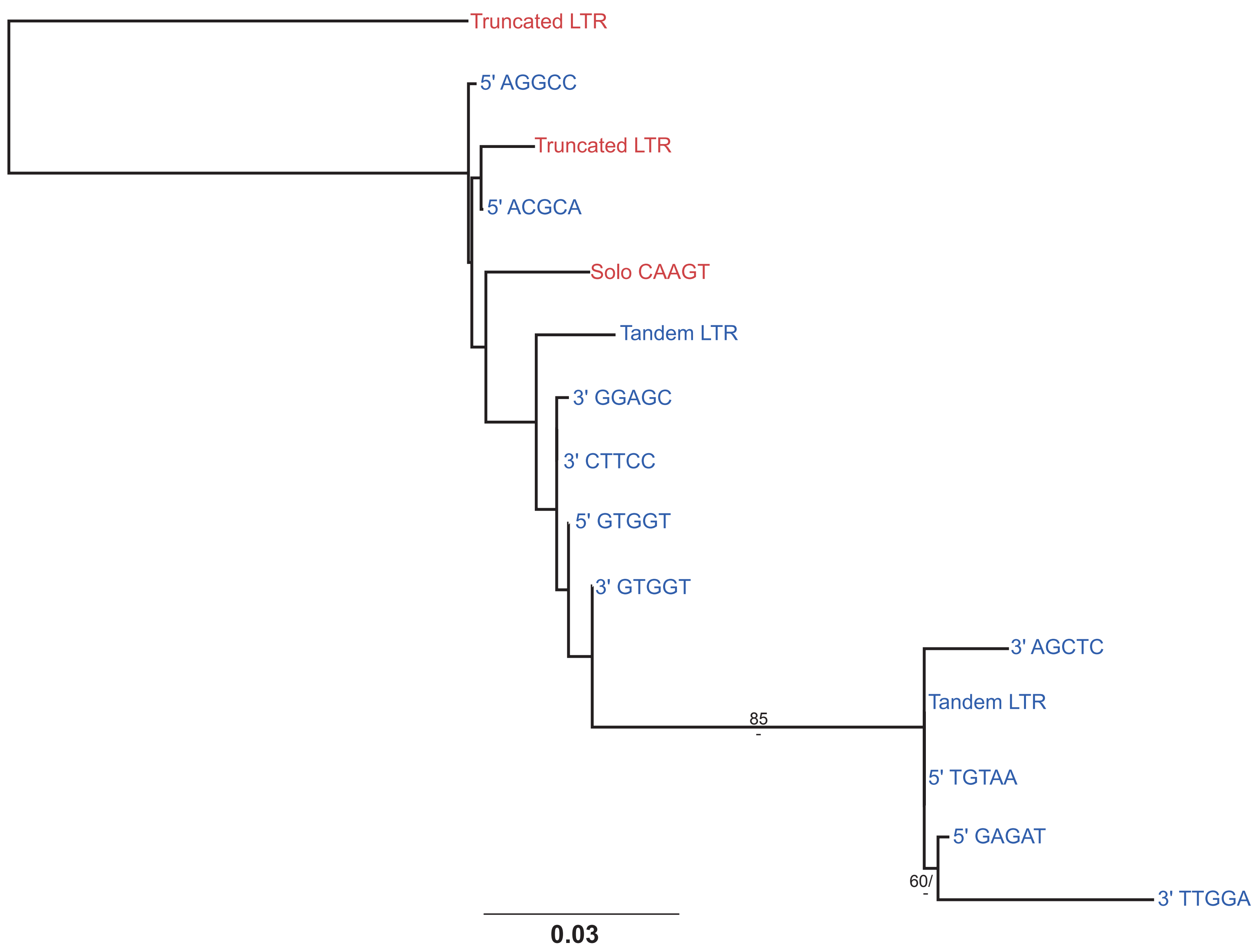
Truncated LTR
5' AGGCC
Truncated LTR
5' ACGCA
Solo CAAGT
Tandem LTR
3' GGAGC
3' CTTCC
5' GTGGT
3' GTGGT
3' AGCTC
85
-
Tandem LTR
5' TGTAA
5' GAGAT
60/
-
3' TTGGA
0.03

(D) ***Sroscv4***

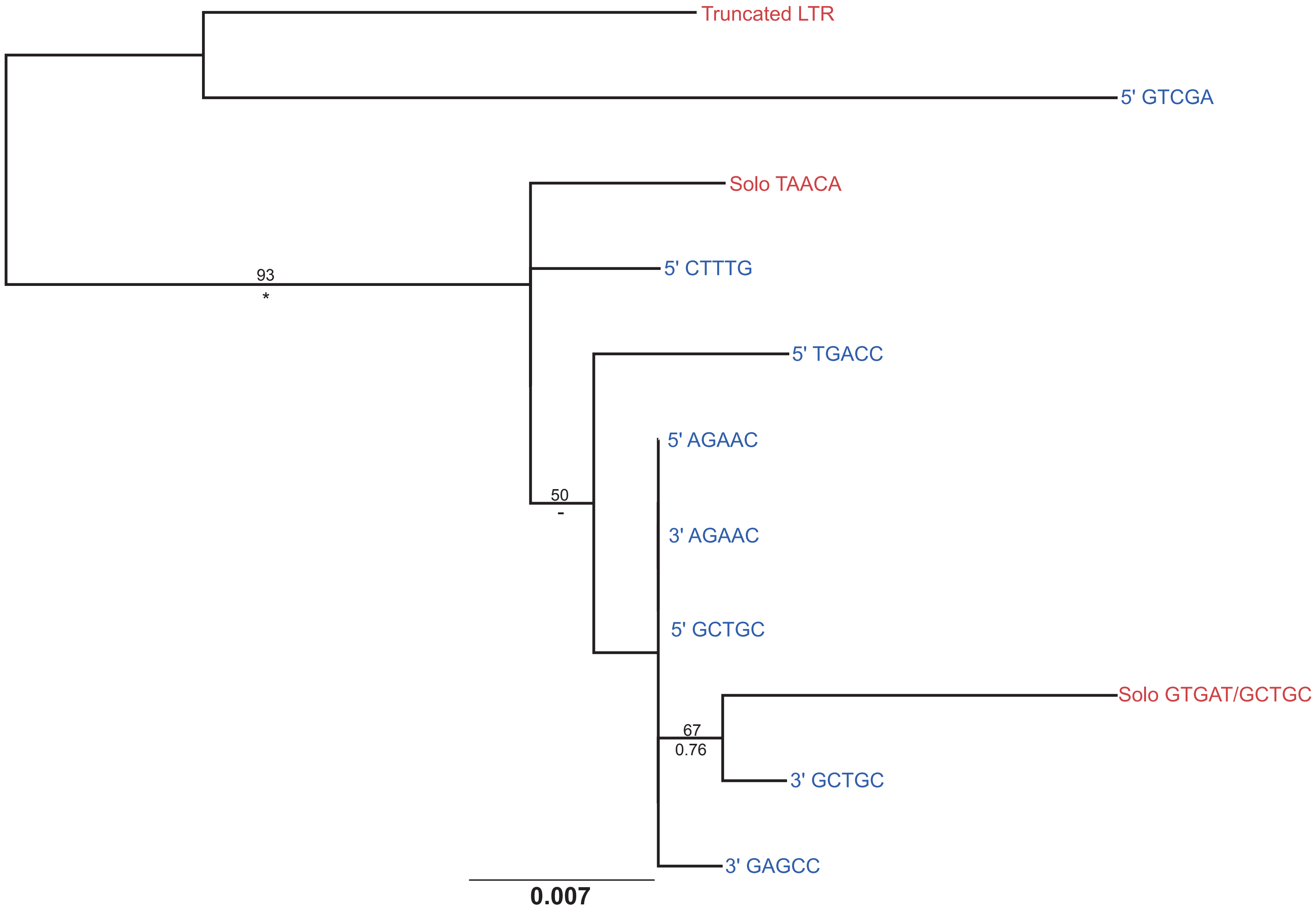
Truncated LTR
5' GTCGA
Solo TAACA
5' CTTTG
93
*
5' TGACC
5' AGAAC
50
-
3' AGAAC
5' GCTGC
Solo GTGAT/GCTGC
67
0.76
3' GCTGC
3' GAGCC
0.007

(E) ***Sroscv5***

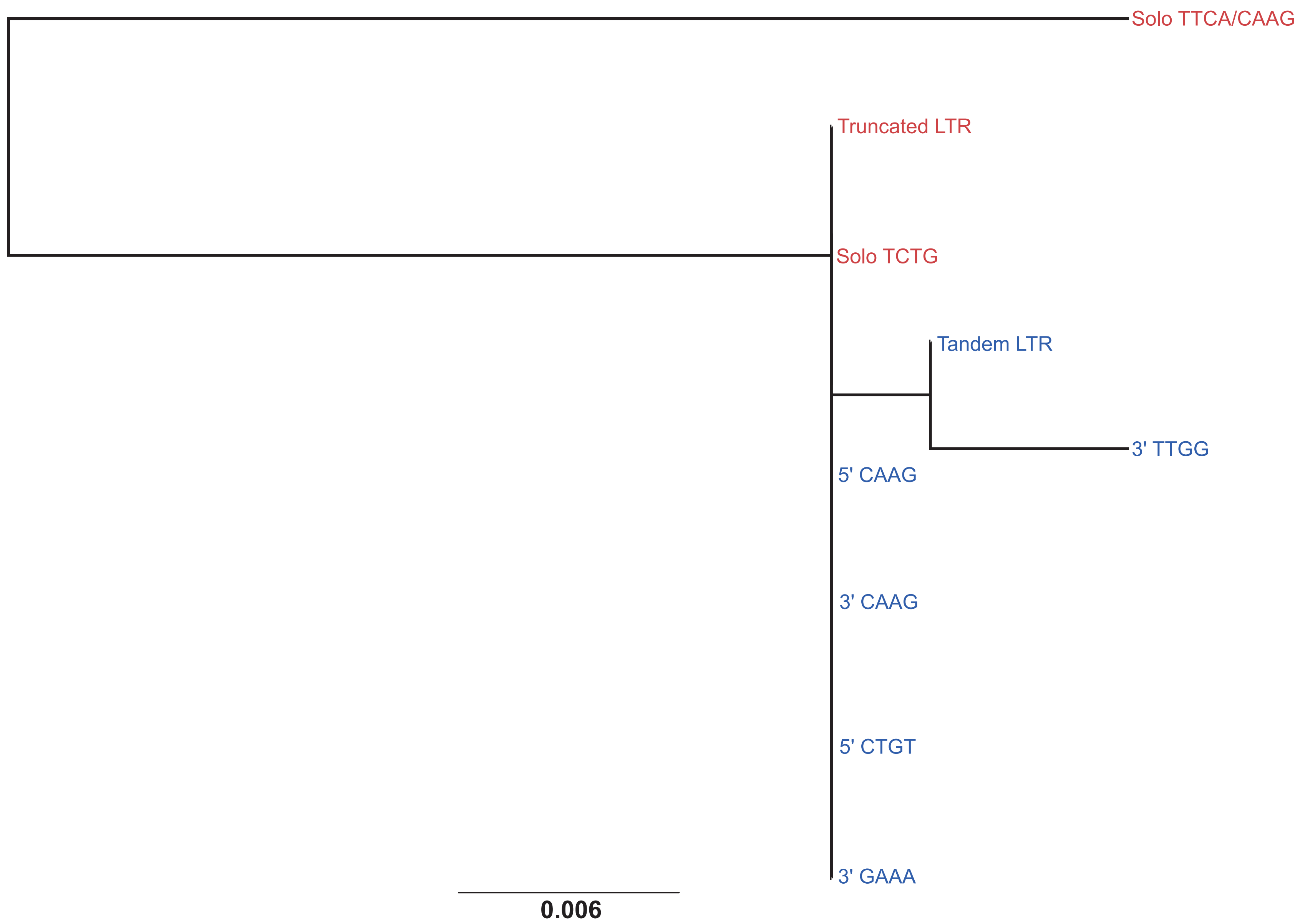
Solo TTCA/CAAG
Truncated LTR
Solo TCTG
Tandem LTR
3' TTGG
5' CAAG
3' CAAG
5' CTGT
3' GAAA
0.006

(F) ***Srosgyp1***

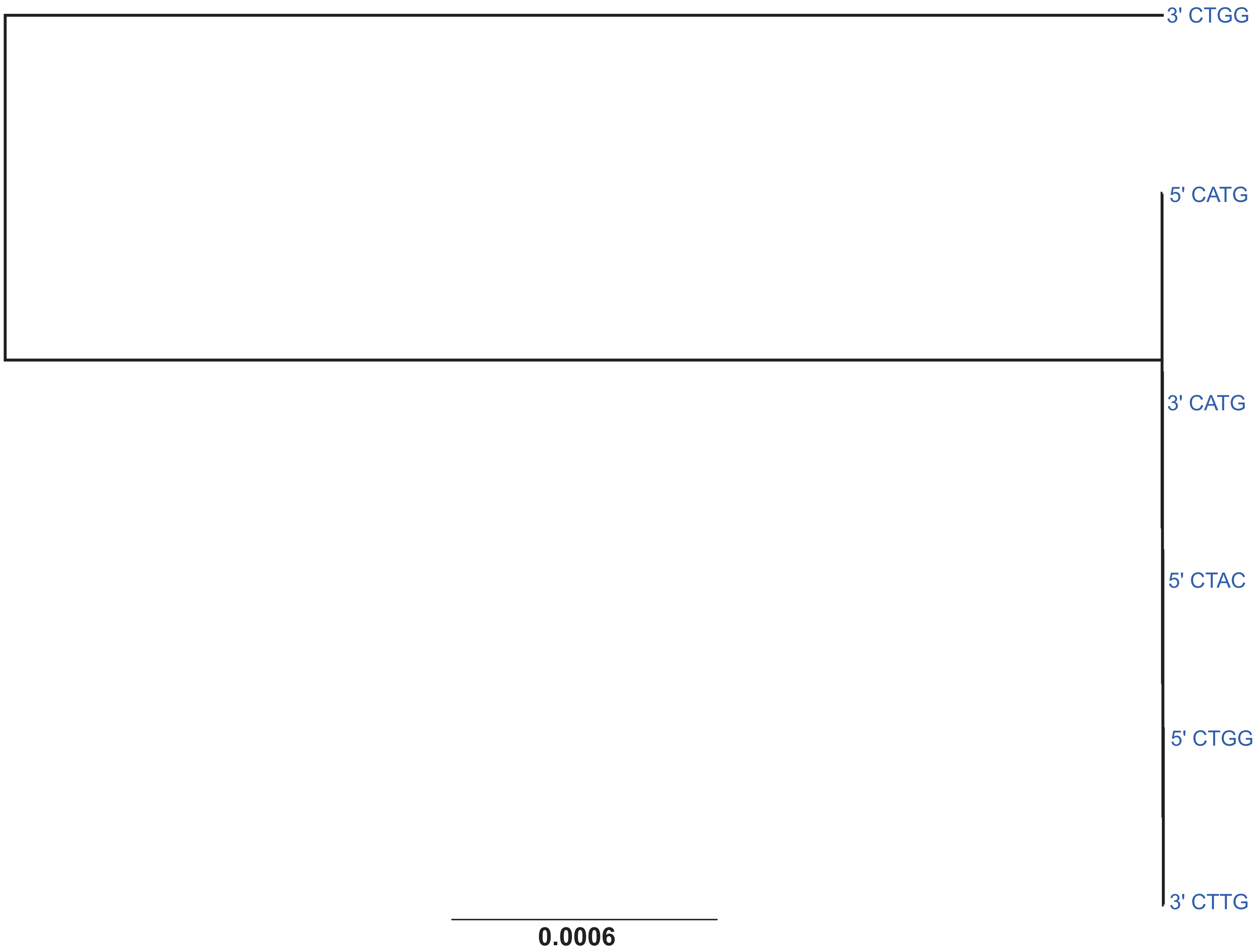

(G) *Srosgyp2*

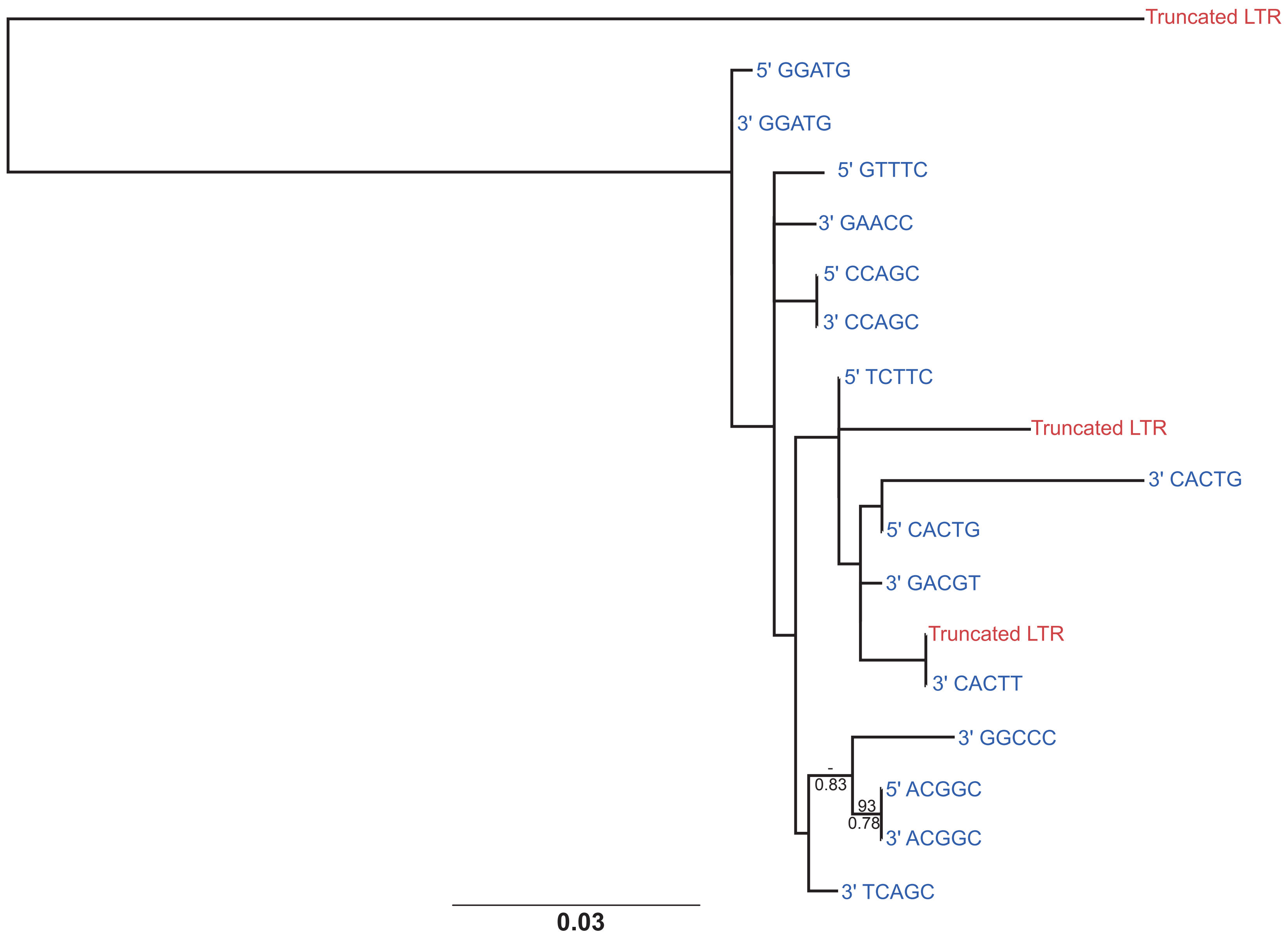
Truncated LTR
5' GGATG
3' GGATG
5' GTTTC
3' GAACC
5' CCAGC
3' CCAGC
5' TCTTC
Truncated LTR
3' CACTG
5' CACTG
3' GACGT
Truncated LTR
3' CACTT
3' GGCCC
-
0.83
5' ACGGC
93
0.78
3' ACGGC
3' TCAGC
0.03

(H) *Srospv1*

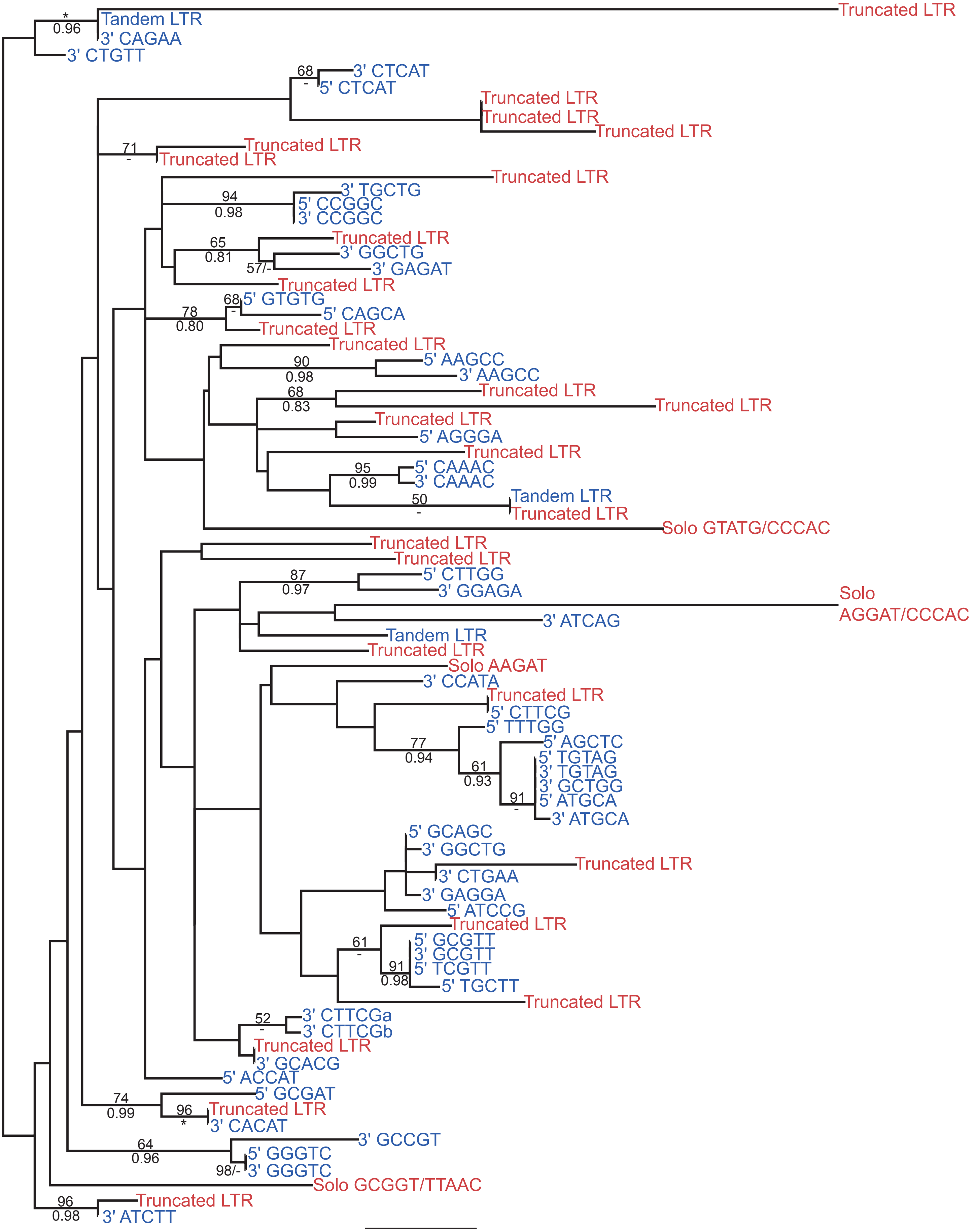
Truncated LTR
Tandem LTR
3' CAGAA
3' CTGTT
3' CTCAT
5' CTCAT
Truncated LTR
3' TGCTG
5' CCGGC
3' CCGGC
3' GGCTG
3' GAGAT
5' GTGTG
5' CAGCA
5' AAGCC
3' AAGCC
5' AGGGA
5' CAAAC
3' CAAAC
Solo GTATG/CCCAC
5' CTTGG
3' GGAGA
Solo
AGGAT/CCCAC
3' ATCAG
Solo AAGAT
3' CCATA
5' CTTCG
5' TTTGG
5' AGCTC
5' TGTAG
3' TGTAG
3' GCTGG
5' ATGCA
3' ATGCA
5' GCAGC
3' GGCTG
3' CTGAA
3' GAGGA
5' ATCCG
5' GCGTT
3' GCGTT
5' TCGTT
5' TGCTT
3' CTTCGa
3' CTTCGb
3' GCACG
5' ACCAT
5' GCGAT
3' CACAT
3' GCCGT
5' GGGTC
3' GGGTC
Solo GCGGT/TTAAC
3' ATCTT
0.02

(I) *Srospv2*

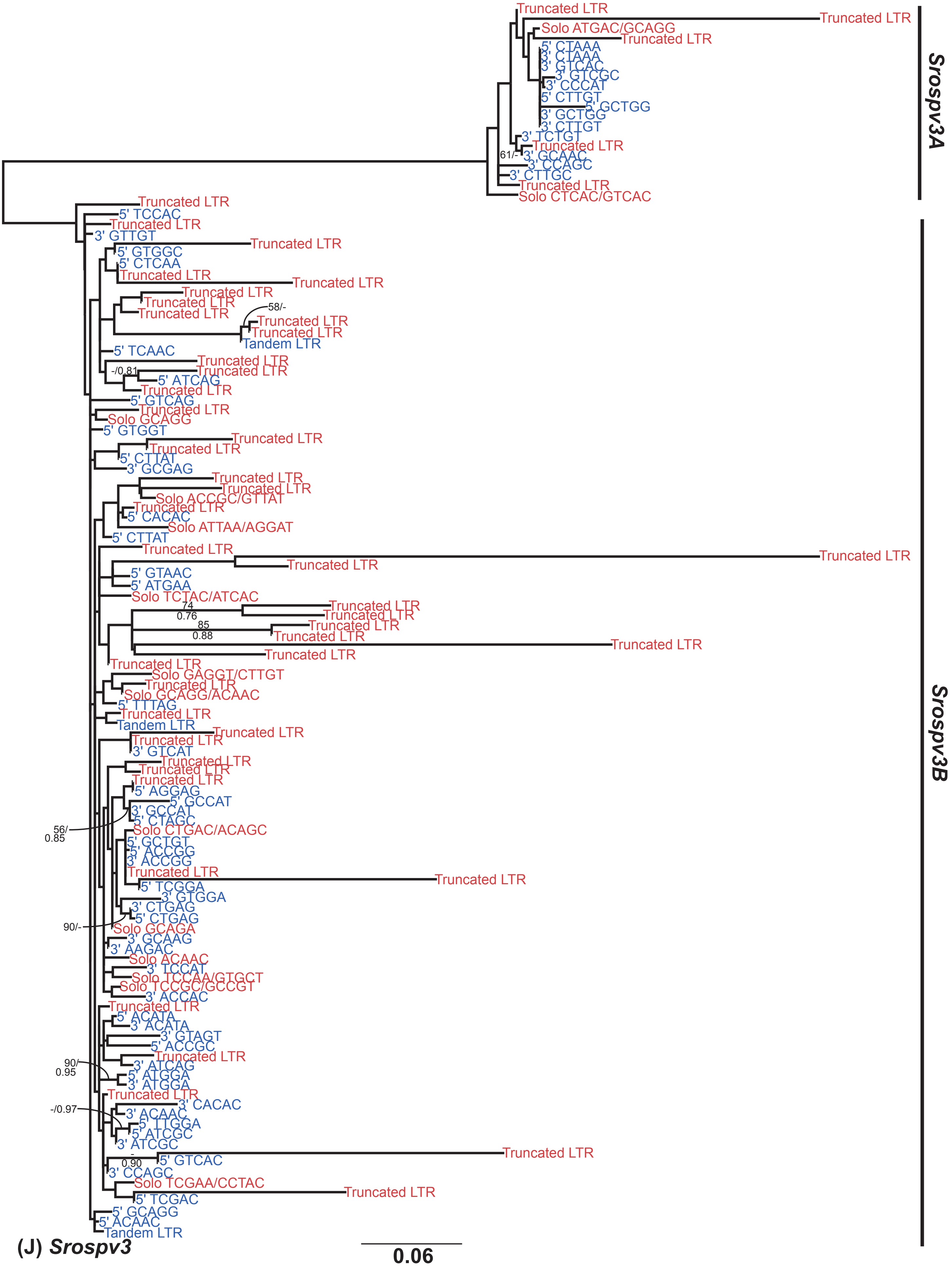
Truncated LTR
Truncated LTR
Solo ATGAC/GCAGG
Truncated LTR
5' CTAAA
3' CTAAA
3' GTCAC
3' GTCGC
3' CCCAT
5' CTTGT
5' GCTGG
3' GCTGG
3' CTTGT
3' TCTGT
Truncated LTR
61/-
3' GCAAC
3' CCAGC
3' CTTGC
Truncated LTR
Solo CTCAC/GTCAC
Srospv3A
Truncated LTR
5' TCCAC
Truncated LTR
3' GTTGT
5' GTGGC
Truncated LTR
5' CTCAA
Truncated LTR
Truncated LTR
Truncated LTR
Truncated LTR
Truncated LTR
58/-
Truncated LTR
Truncated LTR
Tandem LTR
5' TCAAC
Truncated LTR
-/0.81
Truncated LTR
5' ATCAG
Truncated LTR
5' GTCAG
Truncated LTR
Solo GCAGG
5' GTGGT
Truncated LTR
Truncated LTR
5' CTTAT
3' GCGAG
Truncated LTR
Truncated LTR
Solo ACCGC/GTTAT
Truncated LTR
5' CACAC
Solo ATTAA/AGGAT
5' CTTAT
Truncated LTR
Truncated LTR
Truncated LTR
5' GTAAC
5' ATGAA
Solo TCTAC/ATCAC
74
0.76
Truncated LTR
Truncated LTR
85
0.88
Truncated LTR
Truncated LTR
Truncated LTR
Truncated LTR
Truncated LTR
Solo GAGGT/CTTGT
Truncated LTR
Solo GCAGG/ACAAC
5' TTTAG
Truncated LTR
Tandem LTR
Truncated LTR
Truncated LTR
3' GTCAT
Truncated LTR
Truncated LTR
Truncated LTR
5' AGGAG
5' GCCAT
3' GCCAT
56/
0.85
5' CTAGC
Solo CTGAC/ACAGC
5' GCTGT
5' ACCGG
3' ACCGG
Truncated LTR
Truncated LTR
5' TCGGA
3' GTGGA
3' CTGAG
5' CTGAG
90/-
Solo GCAGA
3' GCAAG
3' AAGAC
Solo ACAAC
3' TCCAT
Solo TCCAA/GTGCT
Solo TCCGC/GCCGT
3' ACCAC
Truncated LTR
5' ACATA
3' ACATA
3' GTAGT
5' ACCGC
Truncated LTR
90/
0.95
3' ATCAG
5' ATGGA
3' ATGGA
Truncated LTR
-/0.97
3' CACAC
3' ACAAC
5' TTGGA
5' ATCGC
3' ATCGC
-
0.90
5' GTCAC
Truncated LTR
3' CCAGC
Solo TCGAA/CCTAC
Truncated LTR
5' TCGAC
5' GCAGG
5' ACAAC
Tandem LTR
Srospv3B
0.06

(J) *Srospv3*

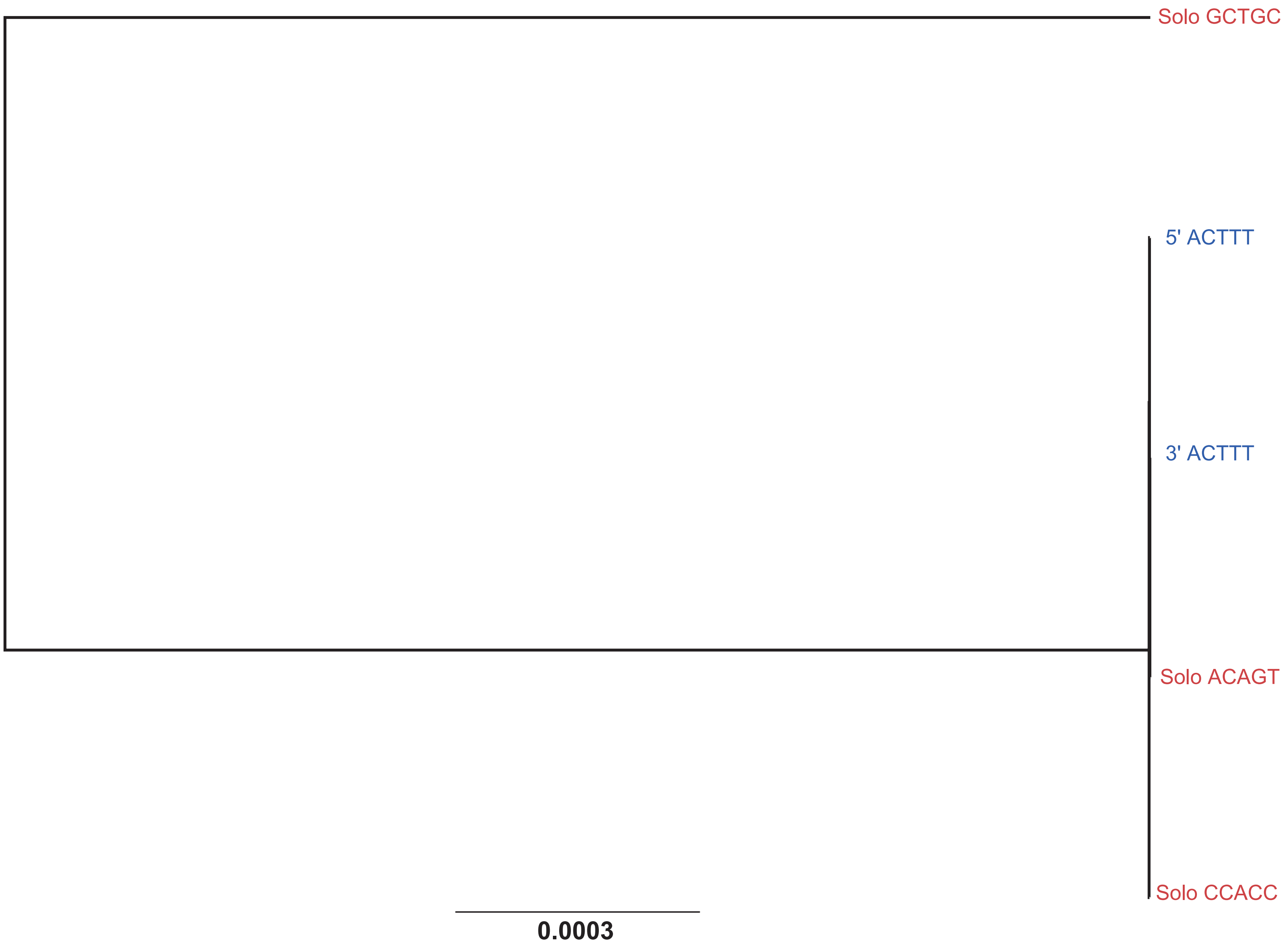

(K) *Srospv4*

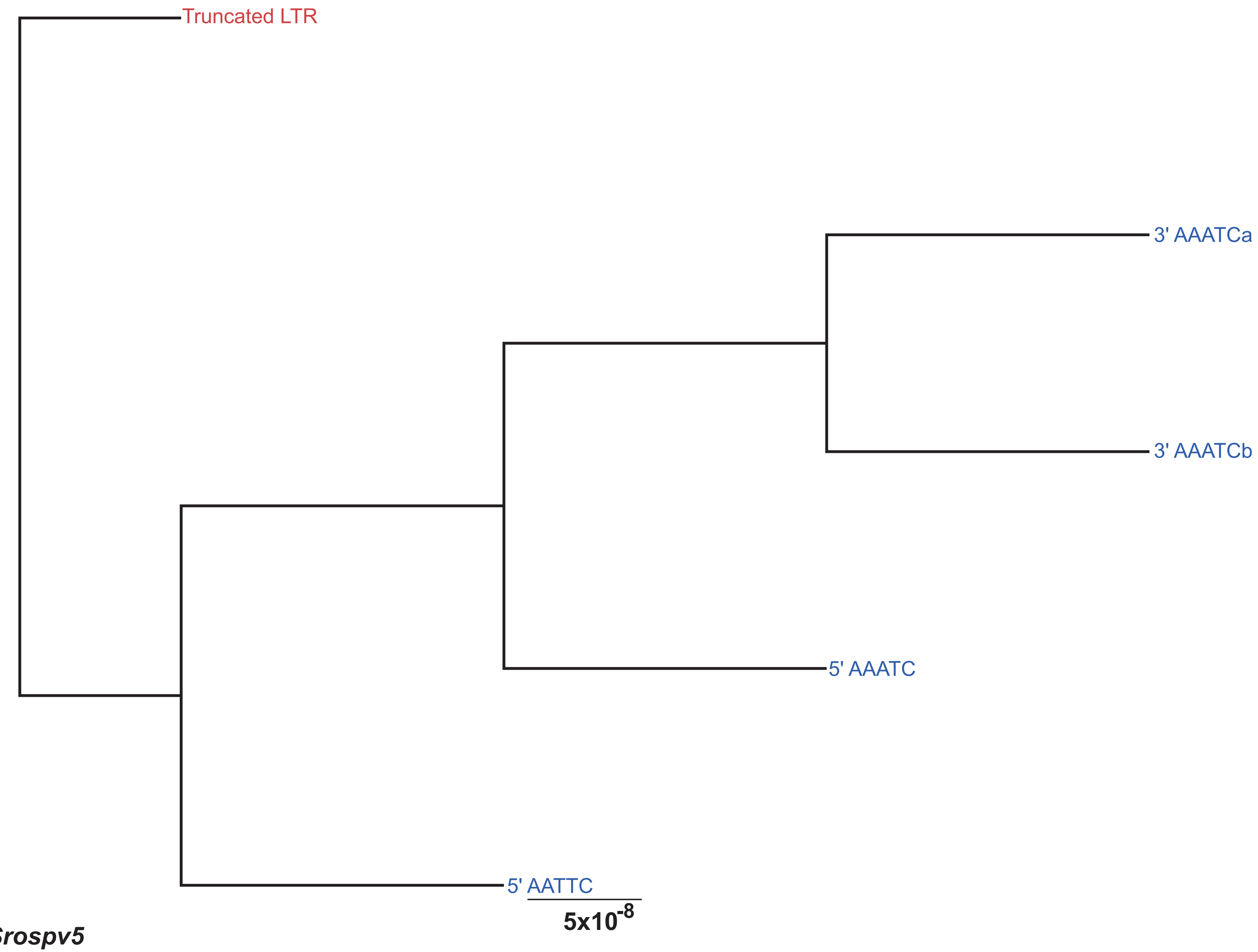

(L) *Srospv5*

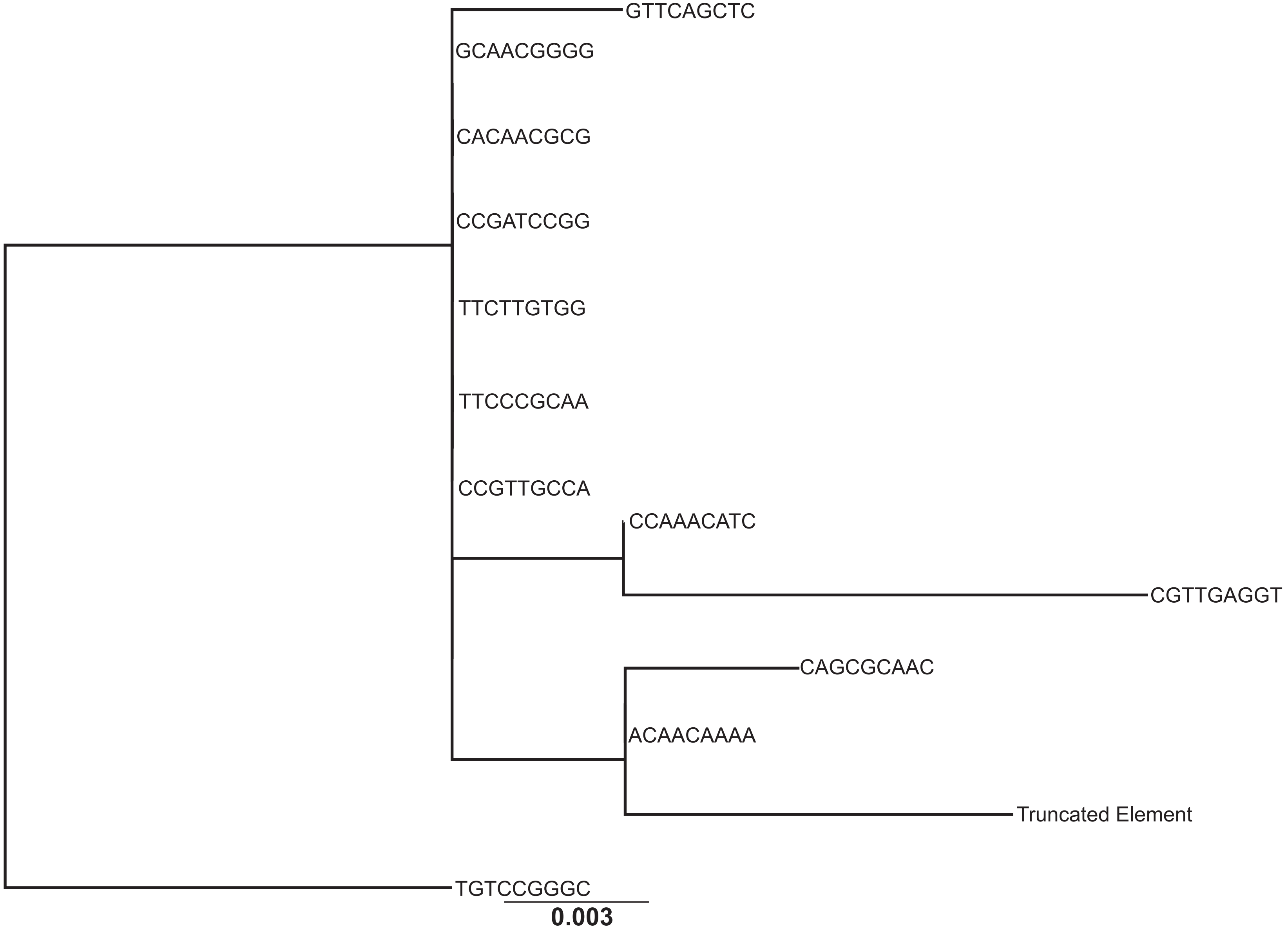
GTTCAGCTC
GCAACGGGG
CACAACGCG
CCGATCCGG
TTCTTGTGG
TTCCCGCAA
CCGTTGCCA
CCAAACATC
CGTTGAGGT
CAGCGCAAC
ACAACAAAA
Truncated Element
TGTCCGGGC
0.003

(M) ***SrosM***

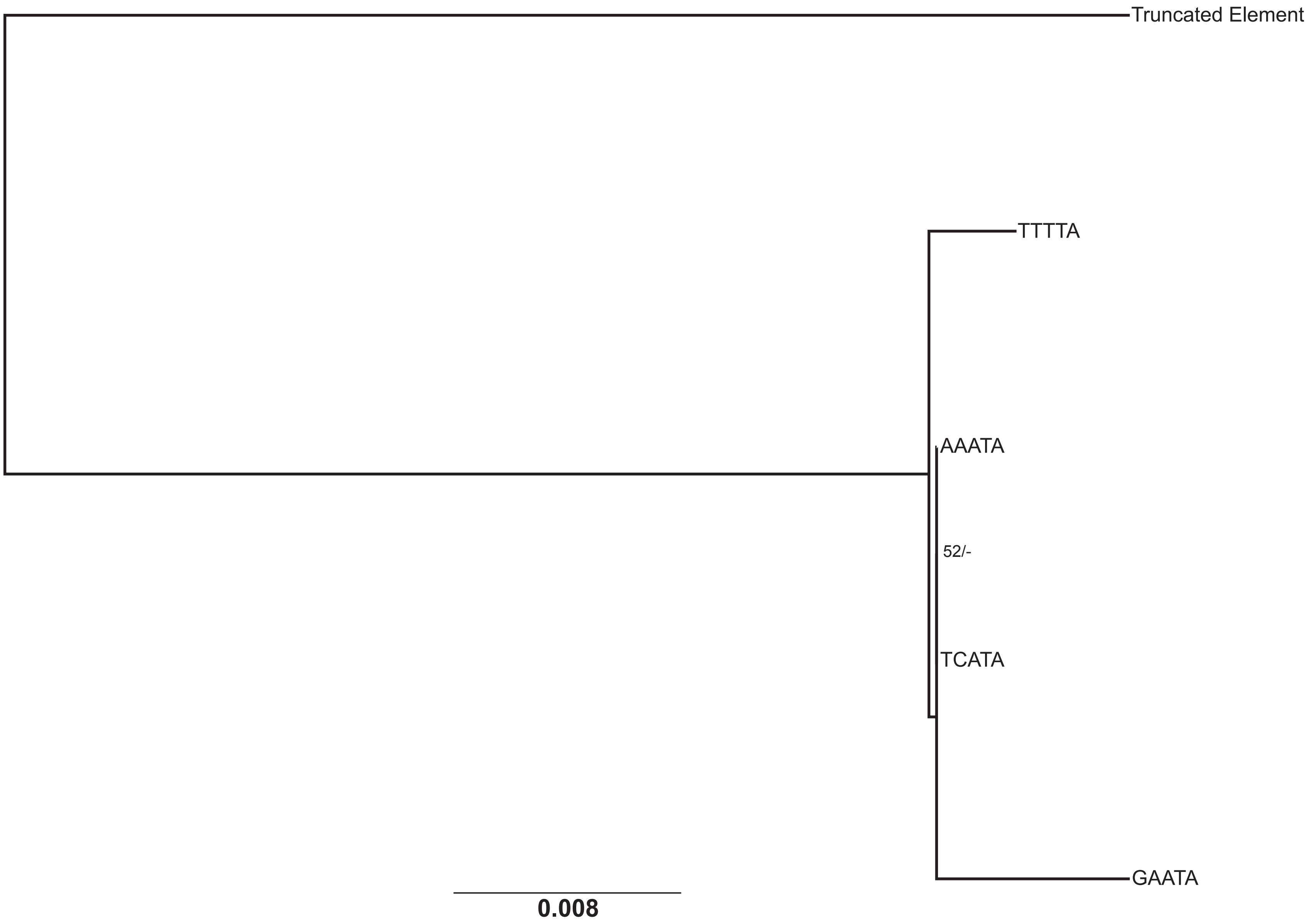

(N) ***SrosTig1***

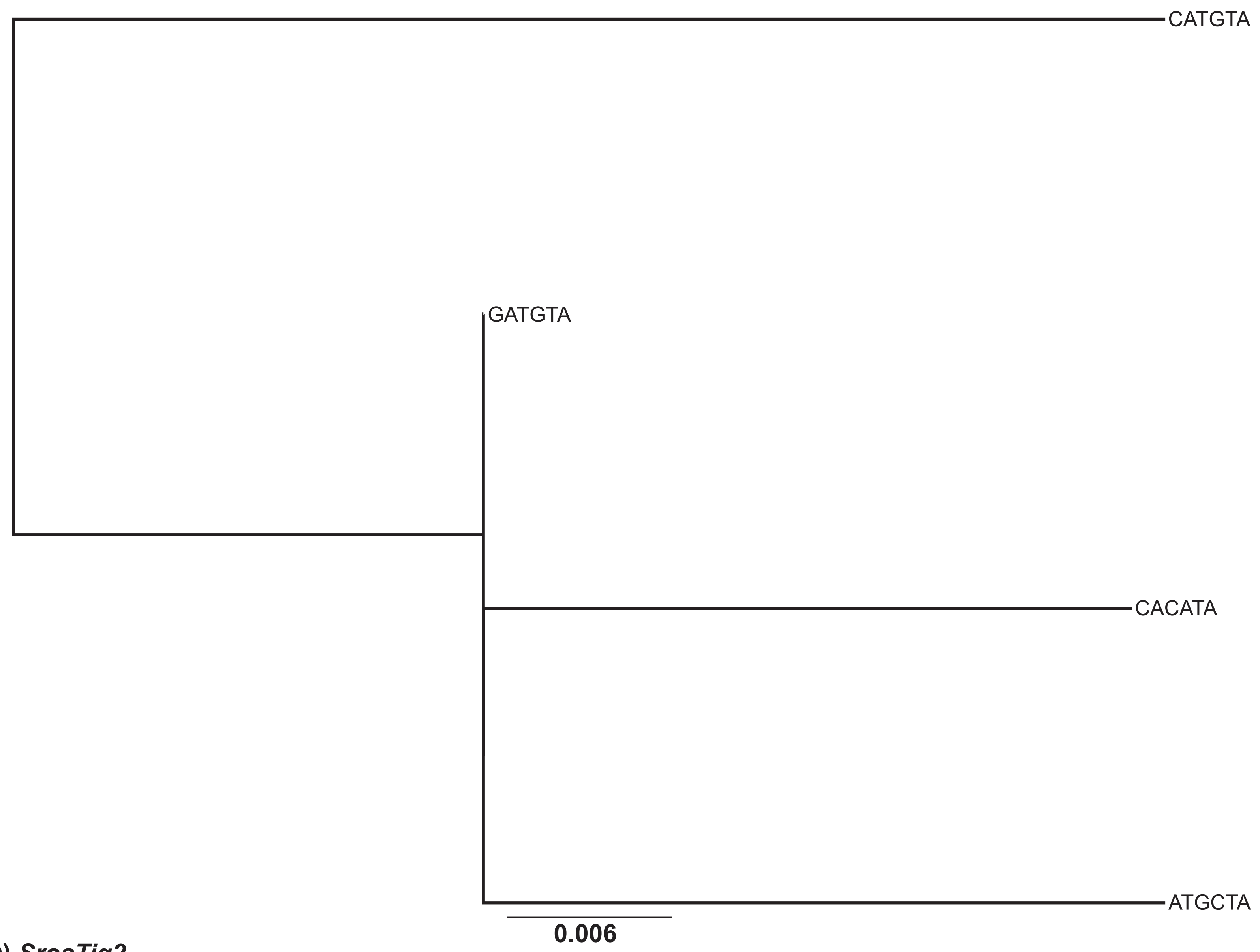

(O) ***SrosTig2***

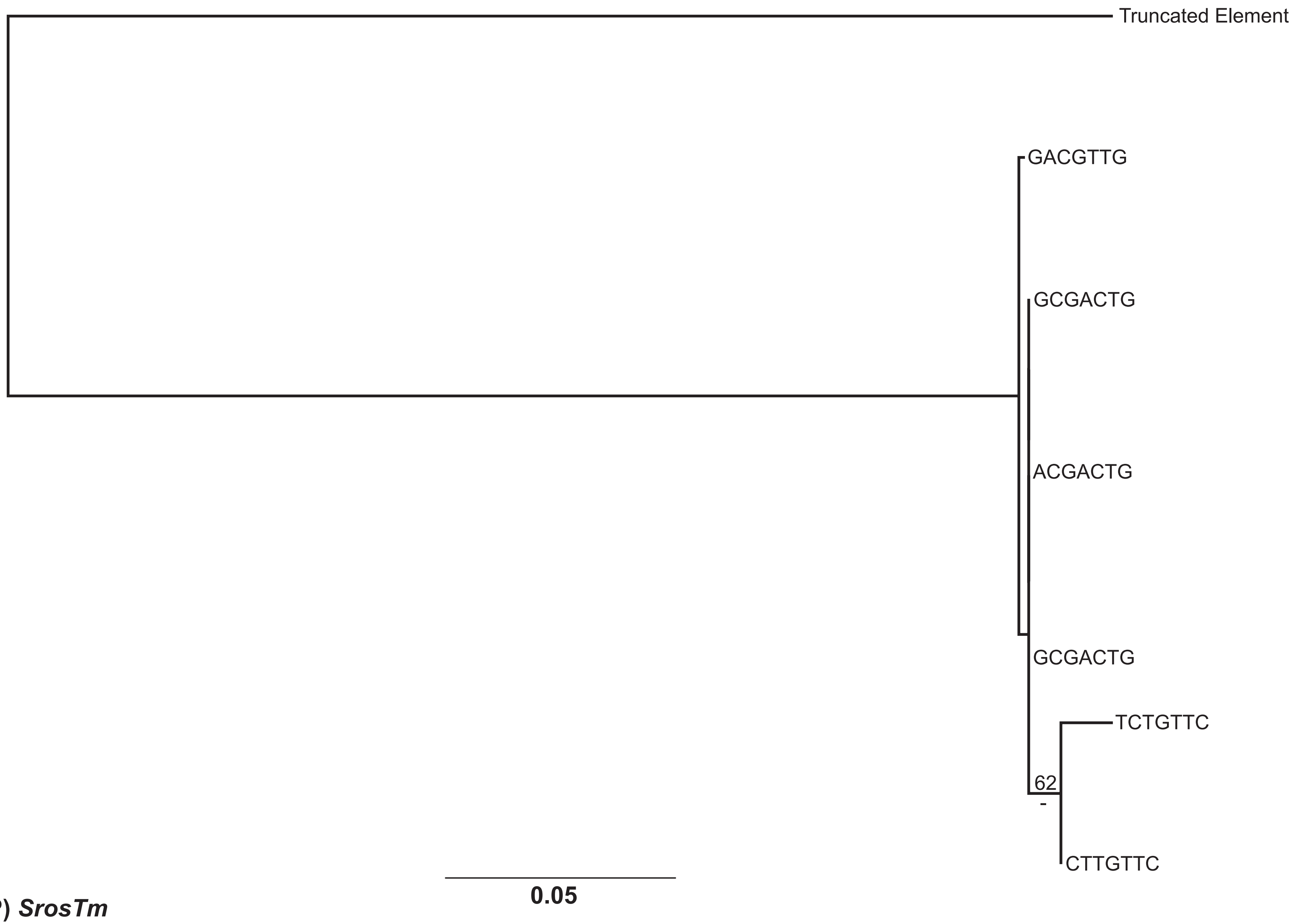
Truncated Element
GACGTTG
GCGACTG
ACGACTG
GCGACTG
TCTGTTC
62
-
CTTGTTC
0.05

(P) *SrosTm*
