# Supplementary material for: A genomic survey of transposable elements in the choanoflagellate Salpingoeca rosetta reveals selection on codon usage
Source: Mob DNA. 2019 Nov 23;10:44. doi: 10.1186/s13100-019-0189-9 (PMC6875170; doi:10.1186/s13100-019-0189-9)
Supplement: Supplementary file 8 — Additional file 8. Preferred codons for each amino acid in the S. rosetta TE families. Green font denotes a favoured codon which complements the product of the major tRNA gene for the amino acid [29]. Blue denotes a favoured codon which does not complements the product of the major tRNA gene for the amino acid, but is a host defined optimal codon [29]. Codons written in red do not complement major tRNA gene products and are not host optimal codons. Black font is used when there is no single favoured codon for the stated amino acid. [file 13100_2019_189_MOESM8_ESM.docx]

**Additional File 8**. Preferred codons for each amino acid in the *S. rosetta* TE families.

| **Amino**  **Acid** | ***Sroscv1*** | ***Sroscv2*** | ***Sroscv3*** | ***Sroscv4*** | ***Sroscv5*** | ***Srosgyp1*** | ***Srosgyp2*** | ***Srospv1*** | ***Srospv2*** | ***Srospv3*** | ***Srospv4*** | ***Srospv5*** |
| --- | --- | --- | --- | --- | --- | --- | --- | --- | --- | --- | --- | --- |
| **Phe** | UUC | UUC | UUC | UUC | UUC | UUC | UUC | UUC | UUC | UUC | UUC | UUC |
| **Leu** | CUG | CUC | CUC | CUC | CUG | CUG | CUG | CUG | CUC | CUG | CUG | CUG |
| **Ile** | AUC | AUC | AUC | AUC | AUC | AUC | AUC | AUC | AUC | AUC | AUC | AUC |
| **Val** | GUG | GUC | GUG | GUG | GUG | GUG | GUG | GUG | GUC | GUC | GUG | GUG |
| **Ser** | UCC | UCA | UCC | AGC | UCG | UCC | AGC | AGC | AGC | AGC | AGC | AGC |
| **Pro** | CCA | CCA | CCA | CCC | CCA | CCC | CCG | CCA | CCC | CCA | CCA | CCA |
| **Thr** | ACG | ACC | ACG/ACA | ACC | ACA | ACC | ACC | ACG | ACG | ACG | ACA | ACA |
| **Ala** | GCC | GCA | GCC | GCC | GCC | GCC | GCC | GCA | GCA | GCC | GCA | GCA |
| **Tyr** | UAC | UAC | UAC | UAC | UAC | UAC | UAC | UAC | UAC | UAC | UAC | UAC |
| **His** | CAC | CAC | CAC | CAC | CAC | CAC | CAC | CAC | CAC | CAC | CAC | CAC |
| **Gln** | CAG | CAG | CAG | CAG | CAG | CAG | CAG | CAG | CAG | CAG | CAG | CAG |
| **Asn** | AAC | AAC | AAC | AAC | AAC | AAC | AAC | AAC | AAC | AAC | AAC | AAC |
| **Lys** | AAG | AAG | AAG | AAG | AAG | AAG | AAG | AAG | AAG | AAG | AAG | AAG |
| **Asp** | GAC | GAC | GAC | GAC | GAC | GAC | GAC | GAC | GAC | GAC | GAC | GAC |
| **Glu** | GAG | GAG | GAG | GAG | GAG | GAG | GAG | GAG | GAG | GAG | GAG | GAG |
| **Cys** | UGC | UGC | UGC | UGC | UGC | UGC | UGC | UGC | UGC | UGC | UGC | UGC |
| **Arg** | CGC | CGC | CGC | CGC | CGC | CGC | CGC | CGC | CGC | CGC | AGA | CGC |
| **Gly** | GGC | GGA | GGC | GGC | GGA | GGC | GGC | GGC | GGC | GGC | GGA | GGA |
| **tRNA** | 14/18 | 13/18 | 15/18 | 15/18 | 13/18 | 15/18 | 13/18 | 12/18 | 15/18 | 14/18 | 11/18 | 12/18 |
| **tRNA &**  **Optimal Codons** | 16/18 | 14/18 | 15/18 | 17/18 | 15/18 | 17/18 | 17/18 | 15/18 | 17/18 | 17/18 | 13/18 | 14/18 |

| **Amino**  **Acid** | ***SrosH*** | ***SrosHar*** | ***SrosM*** | ***SrosS*** | ***SrosTig1*** | ***SrosTig2*** | ***SrosTm*** |
| --- | --- | --- | --- | --- | --- | --- | --- |
| **Phe** | UUC | UUU | UUC | UUC | UUC | UUC | UUC |
| **Leu** | CUG | CUG | CUC | CUC/CUG | CUG | CUC | CUG |
| **Ile** | AUC | AUC | AUC | AUC | AUU/AUC | AUC | AUC |
| **Val** | GUG | GUG | GUC/GUG | GUG | GUG | GUG | GUG |
| **Ser** | UCG | UCC | UCA/AGC | UCG | UCC | AGC | UCC/UCA/AGC |
| **Pro** | CCC | CCU | CCA | CCA | CCA | CCA | CCC |
| **Thr** | ACC | ACC/ACA | ACG | ACC/ACA | ACC | ACA | ACG |
| **Ala** | GCC | GCU | GCC | GCC | GCA | GCA | GCA |
| **Tyr** | UAC | UAC | UAC | UAC | UAC | UAC | UAC |
| **His** | CAC | CAC | CAC | CAC | CAC | CAC | CAC |
| **Gln** | CAG | CAA | CAG | CAG | CAG | CAG | CAG |
| **Asn** | AAC | AAC | AAC | AAC | AAC | AAC | AAC |
| **Lys** | AAG | AAG | AAG | AAG | AAG | AAG | AAG |
| **Asp** | GAC | GAC | GAC | GAC | GAC | GAC | GAC |
| **Glu** | GAG | GAG | GAG | GAG | GAG | GAG | GAG |
| **Cys** | UGC | UGU/UGC | UGC | UGC | UGC | UGC | UGC |
| **Arg** | CGC | CGU | CGC | CGC | CGC | CGC | CGC |
| **Gly** | GGC | GGG | GGC | GGC | GGC | GGC/GGA | GGC |
| **tRNA** | 14/18 | 11/18 | 14/18 | 13/18 | 12/18 | 13/18 | 13/18 |
| **tRNA & Optimal Codons** | 17/18 | 12/18 | 15/18 | 16/18 | 14/18 | 14/18 | 15/18 |

Note: Codons which complement the amino acid’s major tRNA (Southworth et al. 2018) are in green font, while blue font highlights preferred codons which are host optimal codons, but do not complement the major tRNA genes. Codons which do not recognise the major tRNA and are not host optimal codons are in red font. Black font highlights amino acids which do not have a single preferred codon for a given amino acid.
