# Supplementary material for: A genomic survey of transposable elements in the choanoflagellate Salpingoeca rosetta reveals selection on codon usage
Source: Mob DNA. 2019 Nov 23;10:44. doi: 10.1186/s13100-019-0189-9 (PMC6875170; doi:10.1186/s13100-019-0189-9)
Supplement: Supplementary file 9 — Additional file 9. Values of Fop plotted against copy number for TE families in the S. rosetta genome. A) All TE families, B) copia-like families, C) chromoviral families and D) DNA transposon families. The linear line of best fit is shown in red. [file 13100_2019_189_MOESM9_ESM.pdf]

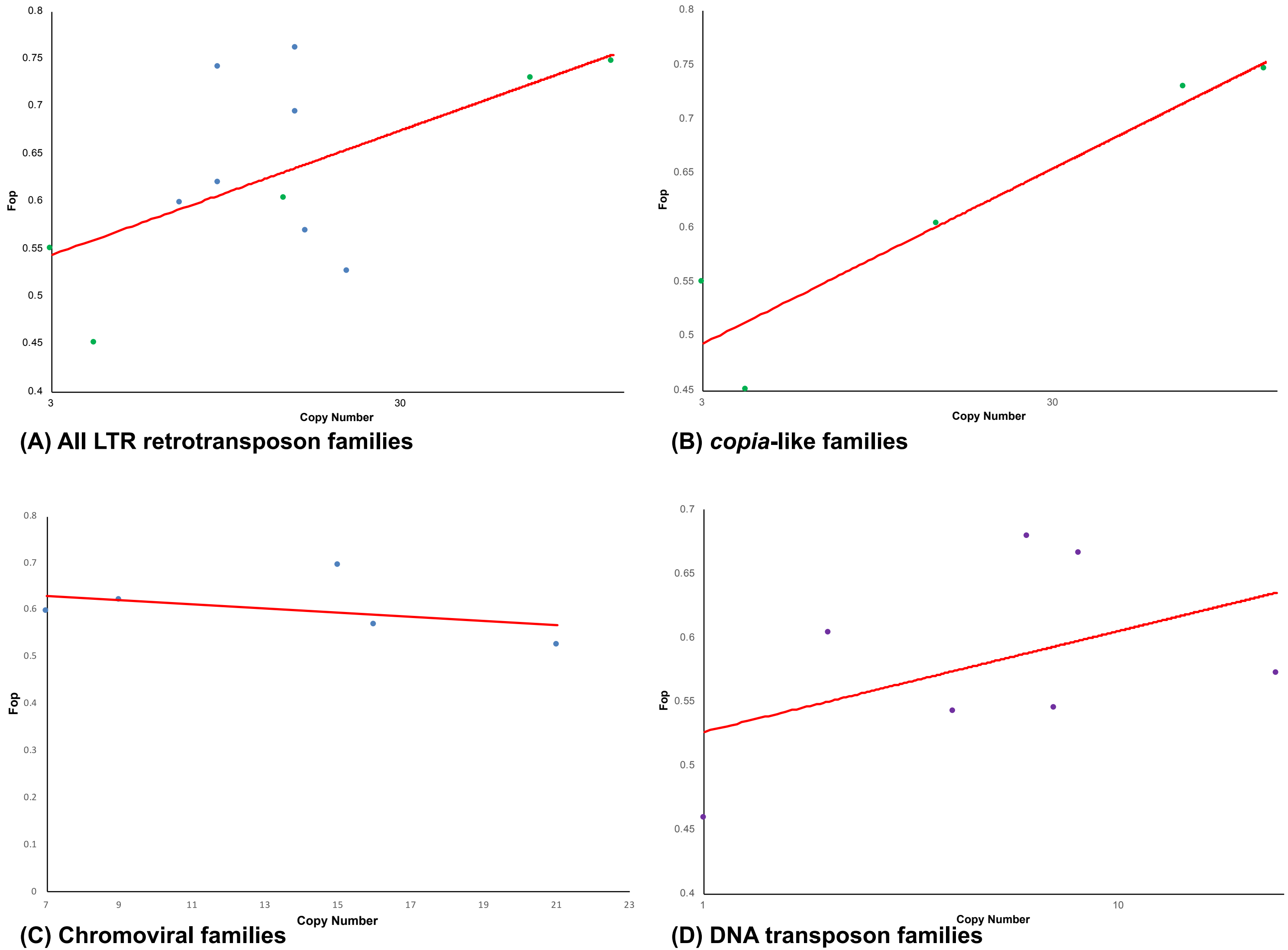
Fop
0.8
0.75
0.7
0.65
0.6
0.55
0.5
0.45
0.4
3
30
Copy Number
(A) All LTR retrotransposon families
Fop
0.8
0.75
0.7
0.65
0.6
0.55
0.5
0.45
3
30
Copy Number
(B) copia-like families
Fop
0.8
0.7
0.6
0.5
0.4
0.3
0.2
0.1
0
7
9
11
13
15
17
19
21
23
Copy Number
(C) Chromoviral families
Fop
0.7
0.65
0.6
0.55
0.5
0.45
0.4
1
10
Copy Number
(D) DNA transposon families
