# Supplementary material for: A genomic survey of transposable elements in the choanoflagellate Salpingoeca rosetta reveals selection on codon usage
Source: Mob DNA. 2019 Nov 23;10:44. doi: 10.1186/s13100-019-0189-9 (PMC6875170; doi:10.1186/s13100-019-0189-9)
Supplement: Supplementary file 10 — Additional file 10. Monosiga brevicollis TE codon usage data. Table 1. Preferred codons for each amino acid in the M. brevicollis TE families. Table 2. Frequency of optimal codons (Fop) in non-domain and domain regions of M. brevicollis TE ORFs. Chart 1. Copy number plotted against Fop for the three M. brevicollis LTR retrotransposons families (copy numbers taken from Carr et al. [10]). Chart 2. GC3s plotted against non-coding GC-content for the three M. brevicollis LTR retrotransposons families. The linear lines of best fit are shown in red. [file 13100_2019_189_MOESM10_ESM.pdf]

Additional File 10. *Monosiga brevicollis* TE codon usage data

Table 1. Preferred codons for each amino acid in the *M. brevicollis* TE families.

**Amino Acid**	***Mbcv1***	***Mbpv1***	***Mbpv2***
**Phe**	UUU	UUU	UUU
**Leu**	CUC	CUG	CUC
**Ile**	AUC	AUC	AUC
**Val**	GUG	GUG	GUC
**Ser**	UCG	ACG	UCU
**Pro**	CCU	CCG	CCC
**Thr**	ACC	ACC	ACC
**Ala**	GCC	GCC	GCC
**Tyr**	UAC	UAC	UAC/UAU
**His**	CAC	CAU	CAC
**Gln**	CAG	CAG	CAG
**Asn**	AAC	AAC	AAC
**Lys**	AAG	AAG	AAG
**Asp**	GAC	GAU/GAC	GAC
**Glu**	GAG	GAG	GAG
**Cys**	UGC	UGC	UGC
**Arg**	CGC	CGC	CGC
**Gly**	GGC	GGC	GGC
**tRNA**	12/18	9/18	13/18
**tRNA & Optimal Codons**	15/18	13/18	15/18

Note: The colour code is the same as Additional File 8.

Table 2. Frequency of optimal codons (F_{op}) in non-domain and domain regions of *M. brevicollis* TE ORFs.

**Family**	**Non-domain F_{op}**	**Domain F_{op}**	**Significance (Fisher's Exact Test)**
Mbcv	0.572	0.608	NS
Mbpv1	0.531	0.603	***P*<0.01**
Mbpv2	0.565	0.618	NS

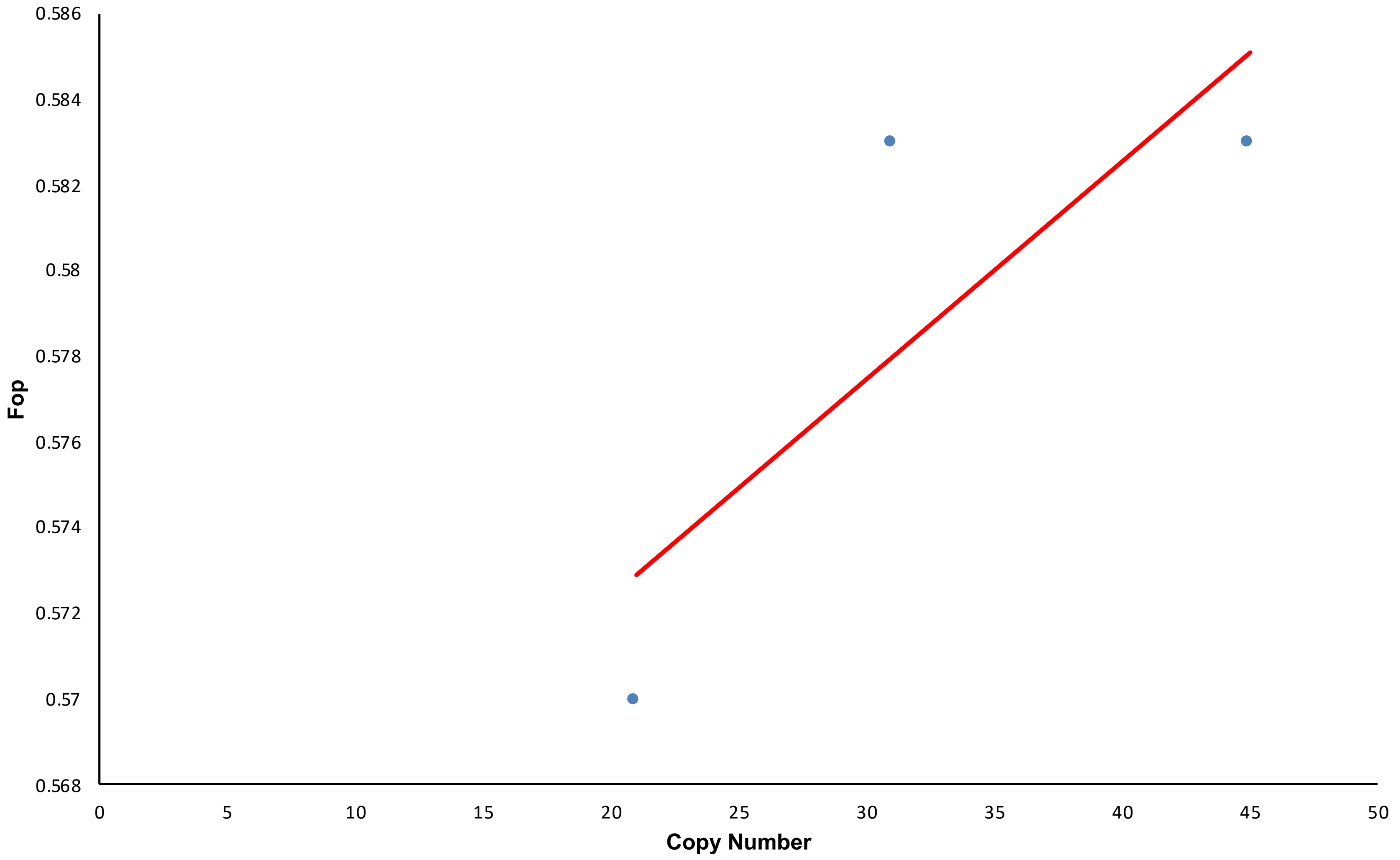

0.586
0.584
0.582
0.58
0.578
0.576
0.574
0.572
0.57
0.568
Fop
0
5
10
15
20
25
30
35
40
45
50
Copy Number

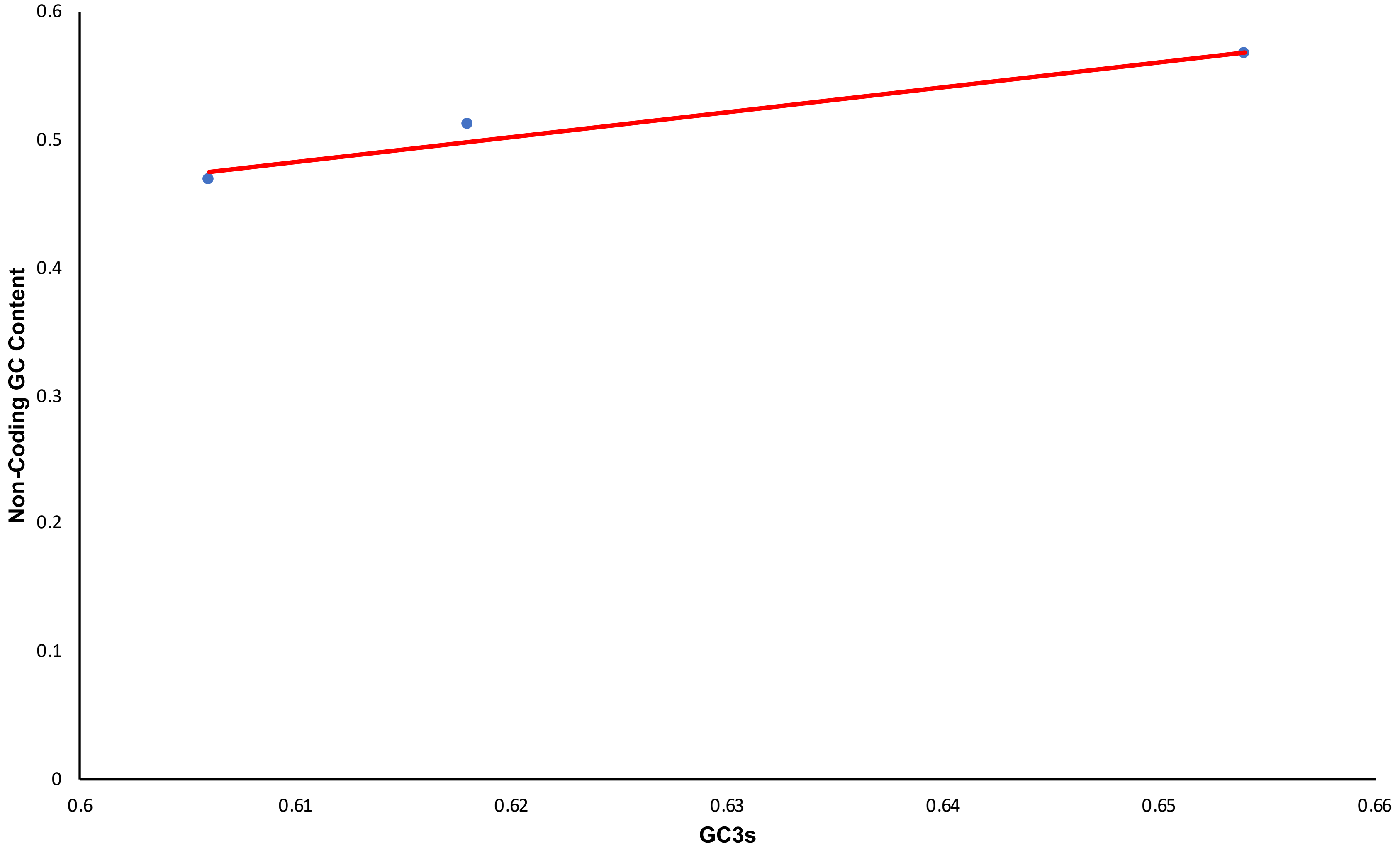

Non-Coding GC Content
0.6
0.5
0.4
0.3
0.2
0.1
0
0.6
0.61
0.62
0.63
0.64
0.65
0.66
GC3s
