# Supplementary material for: A genomic survey of transposable elements in the choanoflagellate Salpingoeca rosetta reveals selection on codon usage
Source: Mob DNA. 2019 Nov 23;10:44. doi: 10.1186/s13100-019-0189-9 (PMC6875170; doi:10.1186/s13100-019-0189-9)
Supplement: Supplementary file 11 — Additional file 11. Codon usage statistics for LTR retrotransposons ORFs in Drosophila melanogaster and Saccharomyces cerevisiae. Values of Nc, GC3s and Fop (determined using host-specific optimal codons) for 26 families of LTR retrotransposons from D. melanogaster and four families from S. cerevisiae. [file 13100_2019_189_MOESM11_ESM.docx]

**Additional File 11**. **Codon usage statistics for LTR retrotransposons in *Drosophila melanogaster* and *Saccharomyces cerevisiae*.**

| **Family^a^** | **GC3s** | ***Nc*** | **F_op_^b^** |
| --- | --- | --- | --- |
| ***D. melanogaster*** |  |  |  |
| *17.6* (X01472) | 0.325 | 48.39 | 0.269 |
| *297* (X03431) | 0.261 | 46.25 | 0.219 |
| *1731* (X07656) | 0.501 | 56.94 | 0.373 |
| *3S18* (U23420) | 0.448 | 55.94 | 0.338 |
| *Blood* (FBgn0000199) | 0.254 | 45.11 | 0.203 |
| *Burdock* (U89994) | 0.379 | 55.73 | 0.286 |
| *copia* (X02599) | 0.279 | 47.92 | 0.218 |
| *DM88* (FBgn0062343) | 0.414 | 56.98 | 0.321 |
| *flea* (Z27119) | 0.403 | 56.06 | 0.333 |
| *GATE* (AJ010298) | 0.488 | 59.89 | 0.362 |
| *Gypsy* (M12927) | 0.544 | 57.33 | 0.436 |
| *HMS-Beagle* (AF365402) | 0.411 | 53.00 | 0.307 |
| *Idefix* (AJ009736) | 0.302 | 48.45 | 0.254 |
| *Max* (AJ487856) | 0.543 | 58.68 | 0.393 |
| *McClintock* (AF541948) | 0.333 | 47.08 | 0.282 |
| *mdg3* (X95908) | 0.333 | 52.12 | 0.251 |
| *micropia* (X14037,X15066) | 0.394 | 57.42 | 0.301 |
| *opus* (AY180918) | 0.455 | 61.00 | 0.348 |
| *Quasimodo* (AF364550) | 0.261 | 45.52 | 0.212 |
| *roo* (AY180917) | 0.277 | 48.89 | 0.201 |
| *Springer* (AF364549) | 0.550 | 58.09 | 0.419 |
| *Tabor* (AC007146) | 0.292 | 46.64 | 0.231 |
| *Tinker* (AC004377) | 0.417 | 57.06 | 0.320 |
| *Tirant* (FBgn0004082) | 0.417 | 53.67 | 0.336 |
| *Transpac* (AF222049) | 0.285 | 50.00 | 0.239 |
| *ZAM* (AJ000387) | 0.365 | 47.67 | 0.297 |
| **Mean** | **0.382± 0.093** | **52.76±5.07** | **0.298± 0.067** |
| ***S. cerevisiae*** |  |  |  |
| *Ty1* (NM_001180048) | 0.303 | 49.96 | 0.443 |
| *Ty2* (CP036478) | 0.293 | 48.53 | 0.436 |
| *Ty3* (CP033477) | 0.274 | 47.22 | 0.432 |
| *Ty4* (M94164) | 0.377 | 51.33 | 0.464 |
| **Mean** | **0.312± 0.045** | **49.26±1.78** | **0.444± 0.014** |

a: GenBank accession numbers or FlyBase identifiers for each family are written in brackets after the family name.

b: F_op_ values are determined using species specific optimal codon in CodonW 1.4.4.
